# Modulation of the intestinal mucosal and cell-mediated response against natural helminth infection in the African catfish *Clarias gariepinus*

**DOI:** 10.1186/s12917-024-04153-1

**Published:** 2024-07-27

**Authors:** Sara Salah Abdel-Hakeem, Yousef Abdal Jalil Fadladdin, Mohsen A. Khormi, Hanan H. Abd-El-Hafeez

**Affiliations:** 1 Parasitology Laboratory, Zoology and Entomology Department, Faculty of Science, Assiut University, Assiut, 71526 Egypt; 2 Faculty of Sciences, Department of Biological Sciences, King Abdulaziz University, Jeddah, Saudi Arabia; 3 Department of Biology, College of Science, Jazan University, Saudi Arabia, P.O. Box. 114, Jazan, 45142 Kingdom of Saudi Arabia; 4 Department of Cell and Tissues, Faculty of Veterinary Medicine, Assiut University, Assiut, 71526 Egypt

**Keywords:** Catfish, Intestinal immune cells, PCNA, VEGF, CD68, Histochemical

## Abstract

Fish gut is a versatile organ serving as the primary pathway for invasion by pathogens, particularly parasites, playing a crucial role in modulating the intestinal adaptive immune response. This study aimed to investigate the cellular-mediated reaction, mucosal acidity, and the expression of proliferating cell nuclear antigen (PCNA), vascular endothelial growth factor (VEGF), and CD68 in the intestines of catfish, *Clarias gariepinus*, naturally infected with helminths. Forty catfish were collected from the Nile River and examined for intestinal parasites. The intestinal tissues of the control and infected fish were fixed for histochemical and immunohistochemical studies. Two groups of helminths were found: cestodes *Tetracampos ciliotheca* and *Polyonchobothrium clarias*, and nematodes *Paracamallanus cyathopharynx*, with a prevalence rate of 63.63%, 18.0%, and 18.0%, respectively. Our results showed that the infected fish had a statistically significant rise in the activity of immune cells, including mast cells, eosinophil granular cells, and dendritic cells. This correlated with upregulation in the expressions of PCNA, VEGF, and CD68. Histochemical analyses demonstrated a marked increase in acidic mucus production, Sudan black B, and bromophenol mercury blue. This study enriches our understanding of the evolution of vertebrate immunity in combating intestinal parasitic infections and the host’s adaptive responses.

**Supplementary Information:**

The online version contains supplementary material available at 10.1186/s12917-024-04153-1.

## Introduction

Fish comprise around 50% of all vertebrates on Earth, making them the most ancient group of vertebrates from a phylogenetic standpoint [1]. Consequently, understanding the immune system of fish would significantly enhanced our knowledge of the evolution of vertebrate immunity [2]. The fish gut is a multifunctional organ, responsible for the processes of digestion, nutrition absorption, and osmoregulation [3]. Moreover, the digestive system of fish closely resembles that of higher vertebrates, which constitute a major site for invasion by ingested pathogens, and immune surveillance. The local and mucosal immune systems of the organism have been extensively investigated by several researchers [4–6]. In contrast to protozoan parasites, helminths elicit a robust adaptive immune response, initiated by excretory and secretory products. These products alter the structure, function, and pattern of the immunological response [7]. Helminths typically induce a long-term infection, by migrating through the host’s tissues, often associated with severe and persistent illness. Hence, parasite infection can cause two types of tissue damage in the host; direct damage caused by the pathogen itself and resulting injury from the immune response (immunopathology). Consequently, the host could develop two types of tolerance mechanisms to reduce immunopathology and pathogen-induced damage [8].

Neutrophils, fibroblasts, macrophages, melano-macrophage centers, and mast cells influence the chronic inflammatory lesions caused by helminth infections in fish. The specific involvement of these cells depends on the type of infection and the species of fish. Similarly, neutrophils, macrophages, and mast cells are the most vigorous categories of immune cells involved in the innate immune response [9]. Moreover, the pathogen plays a crucial role in directing the adaptive immune response of the host through antigen-presenting cells, such as dendritic cells [10].

The mucosal surfaces of all teleosts are protected by adaptive immunity, which depends on T and B cells [11]. These cells possess specific biological traits, that permits them to adjust to the antigenic conditions of the mucosa [12]. Mast cells, often referred to as eosinophilic granular cells, are present in most teleost species, present in various tissues such as the gastrointestinal tract, skin, and gills [13–15]. These cells participate in the regulation of the immune system, healing of tissues, and the formation of new blood vessels [16–18]. Variations in the growth rate of regular cells within the digestive tract could indicate an early malfunction. Proliferating cell nuclear antigen (PCNA) is an ancient protein that plays a direct role in DNA synthesis and cell proliferation [19], which can be identify by immunohistochemical staining techniques [20]. These techniques has determined the localization of nuclear positivity in various organs of mammals [21, 22] and fish [23, 24]. However, altering vascular endothelial growth factor (VEGF), has significant impacts on both the blood vessels in the intestines and the stem cells that make up the intestinal crypt [25]. In fish, CD68-expressing macrophages and acetylcholine (Ach)-expressing neurons in the muscular layer of the intestinal wall [26].

The parasites’ pathogen-associated molecular pattern, binds to the dendritic cell pattern, resulting in the production of surface and secreted proteins, that in conjunction with the antigen, trigger a suitable and well-regulated immune response [7]. In 2022, Alesci et al. observed intestinal dendritic cells in three types of fish: *Clarias batrachus* (Osteichthyes, Teleostei), *Lepisosteus oculatus* (Osteichthyes, Holostei), and *Polypterus senegalus* (Osteichthyes, Brachiopterygii).

This study aimed to evaluate the deficiencies in fish’s intestinal cellular immunity, the distribution of specific immune cells, the acidity of mucus, and the expression levels of PCNA, VEGF, and CD68 in freshwater catfish, *C. gariepinus*, naturally infected with helminth parasites. The objective was to acquire or obtain further information about the host’s response.

## Materials and methods

### Specimen’s collection

A total of 40 catfish with sharp teeth (weighing 320–500 g, total length 35–42 centimeters) were collected from the Nile River by the Assiut Government. The location’s coordinates are 12^0^ degrees 4 min 229 s North latitude, and 10^0^ degrees 48 min 639 s East longitude. Live fish samples were randomly purchased from commercial fishermen at the study site, on a biweekly basis from February to April 2018. The specimens were transferred to the parasitology Laboratory, Zoology and Entomology Department, Faculty of Science, Assiut University, Egypt, in a plastic aquarium for further analysis.

### Parasitological examination

The fish were anesthetized with benzocaine, and the whole alimentary canal was removed via a ventral incision from the anus to the pectoral fin [27]. The gastric and intestinal contents were rinsed on separate petri plates. The intestinal tissues were immersed in a 10% formalin fixative solution for subsequent histological and immunohistochemical investigations.

Live parasites were gently washed in 0.89% cold saline solution to induce relaxation and then preserved in a 4% formalin for morphological identification. The specimens were treated with an aqueous solution of acetocarmine, adhering to (65) method. Nematodes were immersed in a mixture of ethanol (70%) and glycerin (5%) for relaxation, and then mounted on slides using lactopherol as the cleaning agent [28]. The isolated parasites were examined under a microscope (OPTICA microscope) supported with a digital-colored video camera (Optica 4083.B9 Digital Camera, Italy) for the identification of the parasites morphology. The parasites were identified using the identification criteria provided by Jones et al. [29, 30].

### Light microscopy examination

The present work was carried out on the small intestines of eight catfish from control and infected groups. Four samples were used for a light microscopic investigation, whereas the remaining four samples were prepared for semi-thin sections.

### Paraffin processing technique

The samples were immediately fixed in 10% neutral buffered formalin and were further dehydrated in ascending grades of ethanol alcohol (70%, 80%, 90%, and 100%). Afterwards, the samples were cleared with methyl benzoate. Dehydrated samples were then impregnated and embedded in paraplast (Millipore Sigma, St. Louis, MO, USA). Serial 5-µm transverse sections and longitudinal sections were cut with a Leica RM2125 microtome (Leica Microsystems, Wetzlar, Germany), and sections were kept in an incubator at 40 °C to maintain dryness [31, 32]. For general histological examination, the sections were stained with hematoxylin and eosin, PAS techniques, Alcian blue (pH 2.5) combined with PAS, Alcian blue (pH 2.5) combined with safranin O, silver impregnation [33], and mercury bromophenol blue for protein stain.

### Sudan black B for frozen sections

Half-cm^3^ fresh samples of intestine were subjected to freezing sections. After fixation in formal calcium, samples were soaked overnight in optimal cutting temperature compound (OCT) in the fridge, at 4 °C and then stored at -20 °C for further use in cryosection. The tissues were stained with Sudan black B stain, as indicated by Suvarnan et al. (2013).

### Preparations of resin embedding samples for semi-thin sections

The resin-embedding process was employed using Karnovsky’s fixative. The fixative was prepared according to [27, 34]. [10 mL of 25% paraformaldehyde, 10 mL of 50% glutaraldehyde, 50 mL of phosphate buffer, and 30 mL of distilled water (DW)]. The tissue samples were carefully extracted and trimmed to a precise length of 2.0–3.0 mm. Karnovsky fixative was applied at a temperature of 4 °C overnight. The samples were post fixed with osmium tetroxide, followed by washing, drying, and impregnation with a combination of pure resin/alcohol, embedded in resin, and crystallization at 60 °C oven. Propylene oxide (Merck in Darmstadt, Germany) was utilized for a 30-minute process of embedding the resin. The aforementioned process was followed by a 1:1 combination of epoxy resin and propylene oxide for approximately 30 min. Finally, the epoxy resin mixture was used for a duration of 3 h. The epoxy resin mixture was prepared by combining 12 mL of dodecenylsuccinic anhydride (DSAA) with 5 mL of Araldite (Huntsman Advanced Materials, The Woodlands, TX, USA) and 5 mL of EMbed 812 (Polysciences Europe GmbH, Eppelheim, Germany). The samples were immersed in epoxy resin mixture and further subjected to a temperature of 60 °C. An accelerator (2,4,6-Tris[dimethylaminomethyl]phenol; 1.5%) was added to the mixture to initiate the polymerization of the samples. The blocks were incubated for three days, at a temperature of 60 °C, 70 °C and 75 °C for the first, second and third blocks respectively. An ultramicrotome (Reichert-Jung Ultracut E; Leica Microsystems) was used to slice semi-thin sections, with a thickness of 1 μm. Subsequently, toluidine blue dye was applied to the sections [33]. The stained sections were examined using a Leitz Dialux 20 microscope, equipped with a Canon PowerShot A95 digital camera.

### Immunohistochemistry staining procedures for CD68

The Lab Ultra Vision Detection System, a product of Thermo Fisher Scientific, was utilized to localize antigens, using the avidin-biotin complex technique. This system includes an anti-polyvalent, horseradish peroxidase/3,3´-diaminobenzidine (DAB) reagent, which was ready-to-use, as per the manufacturer’s instructions [35]. In brief, according to Soliman et al. [34]; the 5-µm-thick paraffin slices were rinsed three times (5 min/each) in phosphate-buffered solution (PBS) with a pH 7.4. The specimens were dewaxed with xylene and rehydrated progressively with higher concentrations of ethanol. The slices were placed in hydrogen peroxide blocks at room temperature, to prevent the activity of naturally occurring peroxidase. Subsequently, the specimens underwent an additional washing for 10 min under a continuous flow of tap water. The slides underwent antigen retrieval by being exposed to a 10-mmol sodium citrate buffer (pH 6.0; Table 1) for 20 min, at a temperature range of 95 °C to 98 °C in a water bath. The slides were allowed to cool at an ambient temperature for 20 min and were washed in PBS (pH 7.4) three times (5 min/each). The Ultra V Block from Thermo Fisher Scientific was employed to block nonspecific background staining for 5 min at room temperature. After incubating overnight at 4 °C, the primary antibody (Table 1) was applied. The sections were then washed three times with PBS (pH 7.4) for 5 min/each. The sections were incubated with the secondary antibody (Table 1) for 10 min at room temperature. Afterwards, the slides were washed three times for 5 min/each with PBS (pH 7.4) and incubated at room temperature for 10 min with a streptavidin-peroxidase combination (Thermo Fisher Scientific UK and Lab Vision Corporation). A mixture of a single droplet of DAB plus chromogen was combined with 2 mL of DAB plus substrate, which was applied to the sections and incubated for 5 min at room temperature. The Harris haematoxylin counterstain was applied for 30 s. The sections were subjected to xylene for cleaning, followed by two rounds of dehydration in 100% ethanol for 5 min/each. Finally, they were covered with DPX (dibutylphthalate polystyrene xylene) mounting solution. The sections were examined using a Leitz Dialux 20 microscope (Leitz GmbH, Oberkochen, Germany) supported by a Canon PowerShot A95 digital camera (Canon Inc., Tokyo, Japan).

**Table 1:** Components of the Fixative

Fixative	Components	Amount
Bouin’s solution	Picric acid saturated aqueous solution	750 mL
	40% formaldehyde	250 mL
	Glacial acetic acid	50 mL
Karnovsky fixative	Paraformaldehyde, 25% freshly prepared	10 mL
	Glutaraldehyde 50%	10 mL
	Na-phosphate buffer (0.1 M, pH 7.4)	50 mL
	Distilled water	30 mL
Na-phosphate buffer (0.1 M, pH 7.4)	Solution A	
	Na_2_HPO_4_·2H_2_O	17.02 g
	Distilled water	600 mL
	Solution B	
	NaH_2_PO_4_·H_2_	6 g
	Distilled water	200 mL
	Using solution	
	Solution A	580 mL
	Solution B	219 mL
Citrate buffer (pH 6.0)	Solution A	
	Citrate C_6_H_8_O_7_·H_2_O	21 g
	Distilled water	1 L
	Solution B	
	Sodium citrate Na_3_C_6_H_5_O_7_·2H_2_O	29.41 g
	Distilled water	1 L
	Using solution	
	Solution A	9 mL
	Solution B	41 mL
	Distilled water	Add 500 mL

### Immunohistochemical procedures for PCNA and VEGF

A Dako EN Vision + Single Reagent (HRP. Mouse: Agilent Technologies, Inc., Santa Clara, CA, USA) was applied for a two-step immunohistochemical staining of VEGF and PCNA. According to Abdo et al., [36], 5 μm thick sections embedded in paraffin were subjected to dewaxing, rehydration, and washed three times with PBS (pH 7.4) for 5 min/each. A mixture of methanol and drops of 3% hydrogen peroxide was applied to the slides and allowed them to dry at room temperature for 20 min. The slides were washed under running water for an additional 10 min to reduce the activity of endogenous peroxidase. The slides were placed in a sodium citrate buffer with a pH of 6.0 (Table 1) and heated in a tap water bath for 20 min at a temperature of 95–98 °C to extract the antigen. Further, the slides were allowed to cool at room temperature for 20 min, and subsequently rinsed three times with PBS (pH 7.4) for 5 min/ each. A drop of Dako Protein Block (Agilent Technologies, Inc.) was applied and left at room temperature for 5 min to avoid non-specific background staining. As shown in Table 1, the sections were treated with the primary antibody, followed by overnight incubation. Then a secondary antibody was added to the slides at room temperature for 30 min, followed by three to five -minute washing with PBS (pH 7.4), The slides were subjected to another treatment with DAB and substrate-chromogen for 5–10 min at room temperature. Harris hematoxylin was used to counterstain the sections for 30 s. The sections were cleanse with xylene and coated with DPX after two rounds of dehydration in 90% and 100% ethanol each lasting five minutes. The immunohistochemical staining was assessed using a Leitz Dialux 20 microscope and a Canon PowerShot A95 digital camera. A variant standard control that did not include the use of primary antibodies was employed to produce negative control samples.

### Quantitative analysis

Three pathologists independently scored the number of goblet cells and mast cell positivity, using PCNA and VEGF on three different slides per infected and control animals, using a high-power (400 ×) light microscope (OPTICA, Italy). The number of positive cells was determined across ten random fields, in each slide and expressed it as a mean [37].

We utilized Image J software in the following way for detection: we opened each image individually using Image J Fiji program. Choose “type” and then “8-bit” to convert the picture to an 8-bit image from the image column. Next, we select a measurement from the “analyze column” and determine the area as well as the area fraction. Choose “adjust” from the “image column” and then “threshold.” We will select default, red, and dark backgrounds from the pull-down choices, then threshold the image by dragging the top side until the whole foreground is red. Lastly, we try to keep the stained area as consistent as possible, click “Apply,” and the percentage of the area will be measured from the “analysis column.” [31].

### CMEIAS Segmentation (All negative figures in the supplementary file)

A negative image was created using CMEIAS Segmentation. This is a free, enhanced computational method that processes color photographs, by separating the foreground object of interest from the background. The steps entail: opening the image with CMEIAS Color Segmentation, then pick “process” from the menu, followed by “negative image.”

[36–40, 66].

### Statistical analysis

The data was meticulously loaded into a spreadsheet in Microsoft Excel 2010 and then analyzed using SPSS (version 27) for Windows 10. The number of cells were estimated using the mean and standard deviation (SD), and the unpaired t-test and post hoc Duncan multiple range were performed for group comparison (Fouad et al., 2024). Statistical significance was typically determined at a *P* value < 0.05.

## Results

### Prevalence of helminth infection

**Fig. 1:**
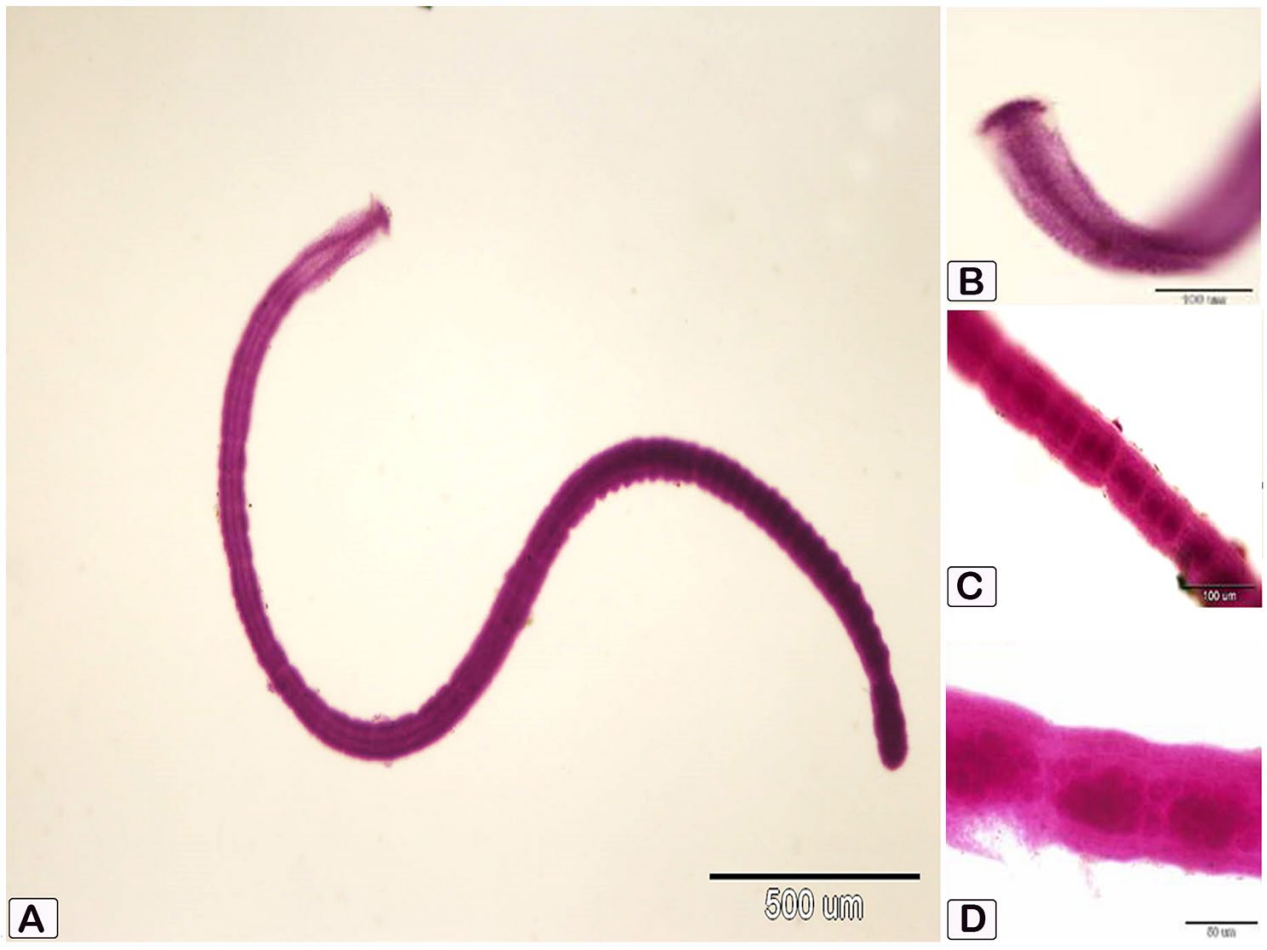
Photomicrograph shows the morphological characters of cestodes, *Tetracampos ciliotheca*, recovered from the intestine of African catfish *C. gariepinus*. (**A**) Whole mount of the full-length worm; (**B**) Higher magnification showing elongated scolex, armed with 25–40 hooks arranged in two lateral semicircles, having a narrower and tapering end during movement, and contracted into a mushroom shaped structure at rest; (**C**) High magnification showing mature proglottids with female genital system in the middle of proglottids; (**D**) High magnification of gravid proglottids showing uterus filled with eggs

**Fig. 2:**
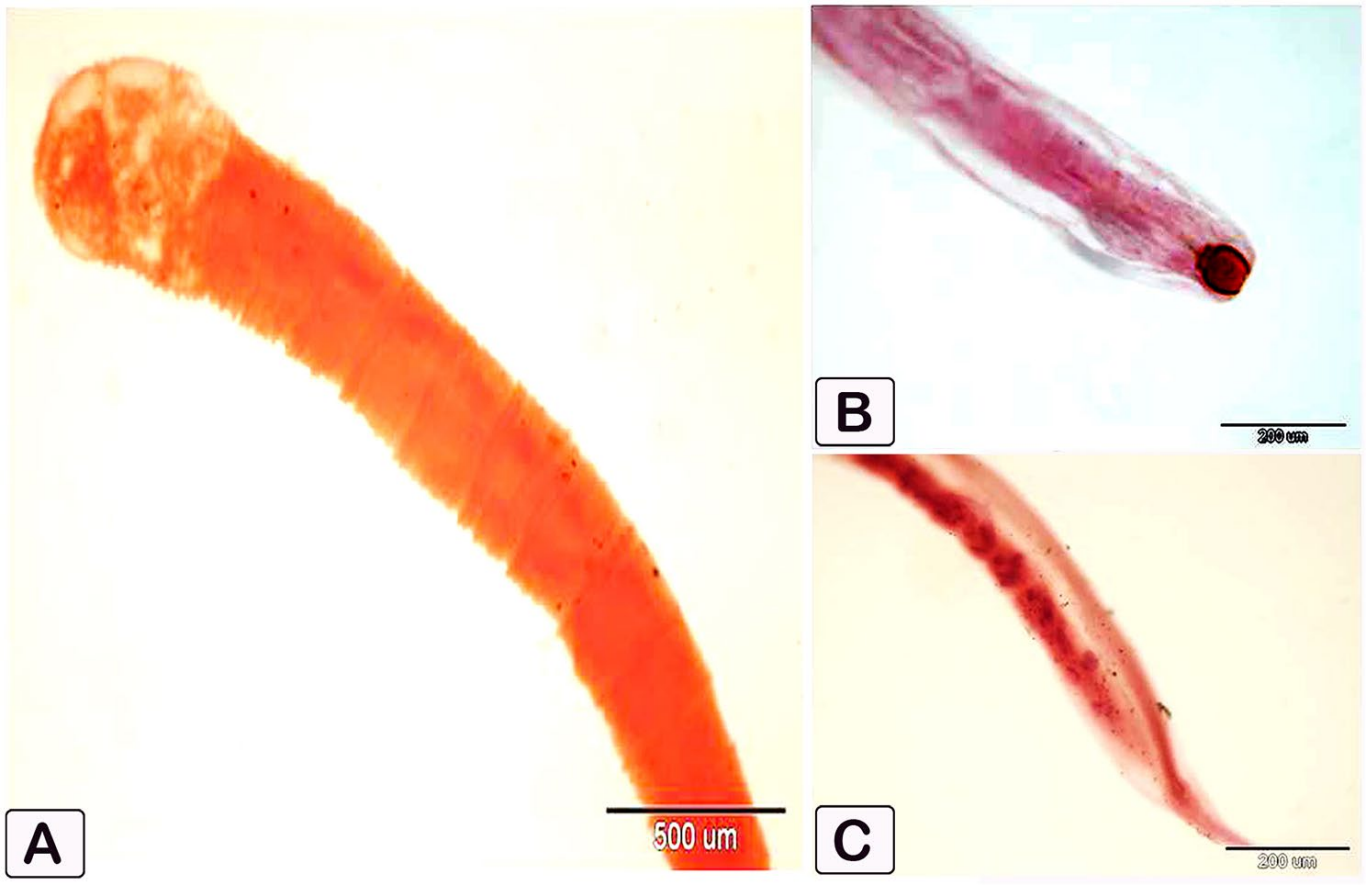
Photomicrograph shows helminth parasites recovered from the intestine of African catfish *C. gariepinus*. (**A**) Whole mount of *Polyonchobothrium clarias* showing rectangular scolex with a flat to slightly raised apex (rostellum) armed; (**B**) Anterior end of *Paracamallanus cyathopharynx* showing circular mouth and barrel shaped buccal capsule, with simple basal ring; (**C**) Tapered posterior end of *Paracamallanus cyathopharynx*

Intestinal parasites were found in 18 (45%) of the total fish examined. Two groups of helminths were found: cestodes *Tetracampos ciliotheca* (Fig. 1) and *Polyonchobothrium clarias* (Fig. 2a), and nematodes *Paracamallanus* spp. (Fig. 2b and c) with a prevalence rate of 63.63%, 18.0%, and 18.0%, respectively. The females showed high infection rate than the males (Table 2 and Table 3).

**Table 2:** Identity, sources, and working dilution of antibodies used in the present immunohistochemical analysis

Primary antibody	Supplier	Origin	Dilution	Incubation	Antigen retrieval	Biotinylated secondary antibody
CD68 (Macrophage Marker) Ab-3 (Clone KP1)	Mouse Anti-CD68 (Thermo Fisher Scientific Lab Vision Corporation, Fremont, USA)	Mouse Monoclonal Antibody Cat. #MS-397-R7	1:100	Over night	boiling in citrate buffer (pH 6.0), 20 min	Goat anti-Mouse IgG (H + L) Secondary Antibody (Catalog # 31,569) Dilution ; 1:100 One hour at room temperature
VEGF	Rabbit anti -VEGF (Thermo Fisher Scientific Waltham, MA, USA))	Rabbit VEGF Polyclonal Antibody (clone: RB-222- P0) (Cat.no PA1- 21,796)	1:100	Over night	boiling in citrate buffer (pH 6.0), 20 min	Goat anti-rabbit secondary antibody (Cat. no. K4003, EN Vision + TM System Horseradish Peroxidase Labelled Polymer; Dako). Ready to use (30 min at room temperature)
PCNA	Mouse Recombinant Monoclonal PCNA antibody (Abcam)	Monoclonal antibody (Cat.no ab264494)	1:200	Over night	boiling in citrate buffer (pH 6.0), 20 min	Goat anti-rabbit secondary antibody (Cat. no. K4003, EN Vision + TM System Horseradish Peroxidase Labelled Polymer; Dako). Ready to use (30 min at room temperature)

**Table 3:** Prevalence of intestinal parasites in relation to sex

	Males	Females	Total
Number of fish examined	24	16	40
Number of infected	8	10	18
Prevalence (%)	33.3%	62.5%	45%

### Light microscopy

**Immunological cells are associated with parasite infection.**

### Goblet cells

**Fig. 3:**
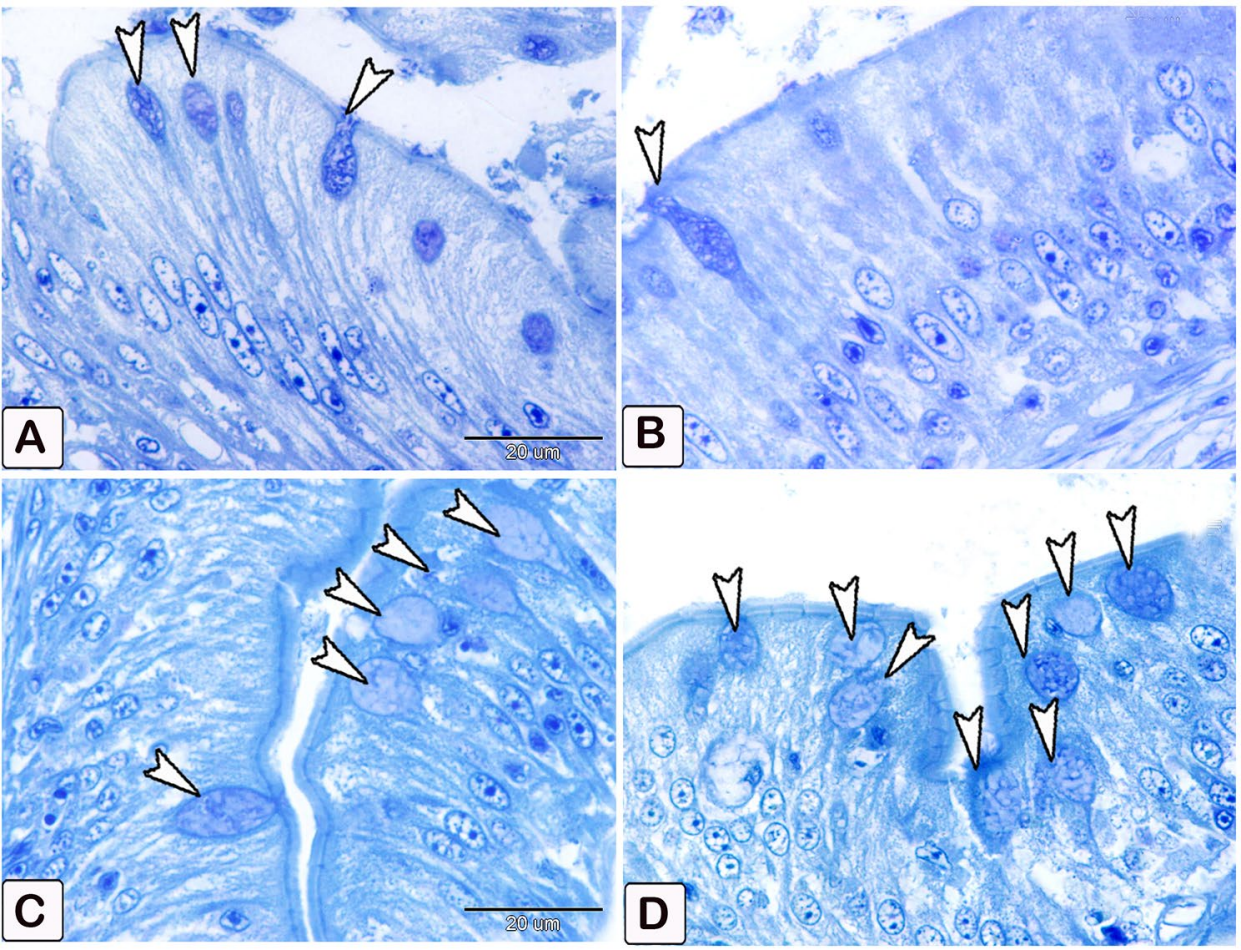
Semithin section of the intestines of African catfish *C. gariepinus* stained with toluidine blue showing difference in the number of goblet cells between the control and the infected fish. (**A** and **B**) Intestine of control fish exhibiting a limited number of goblet cells within the columnar epithelium (arrowhead). (**C** and **D**) Intestine of infected fish showing a greater number of goblet cells in the columnar epithelium compared to the control group (arrowheads)

**Fig. 4:**
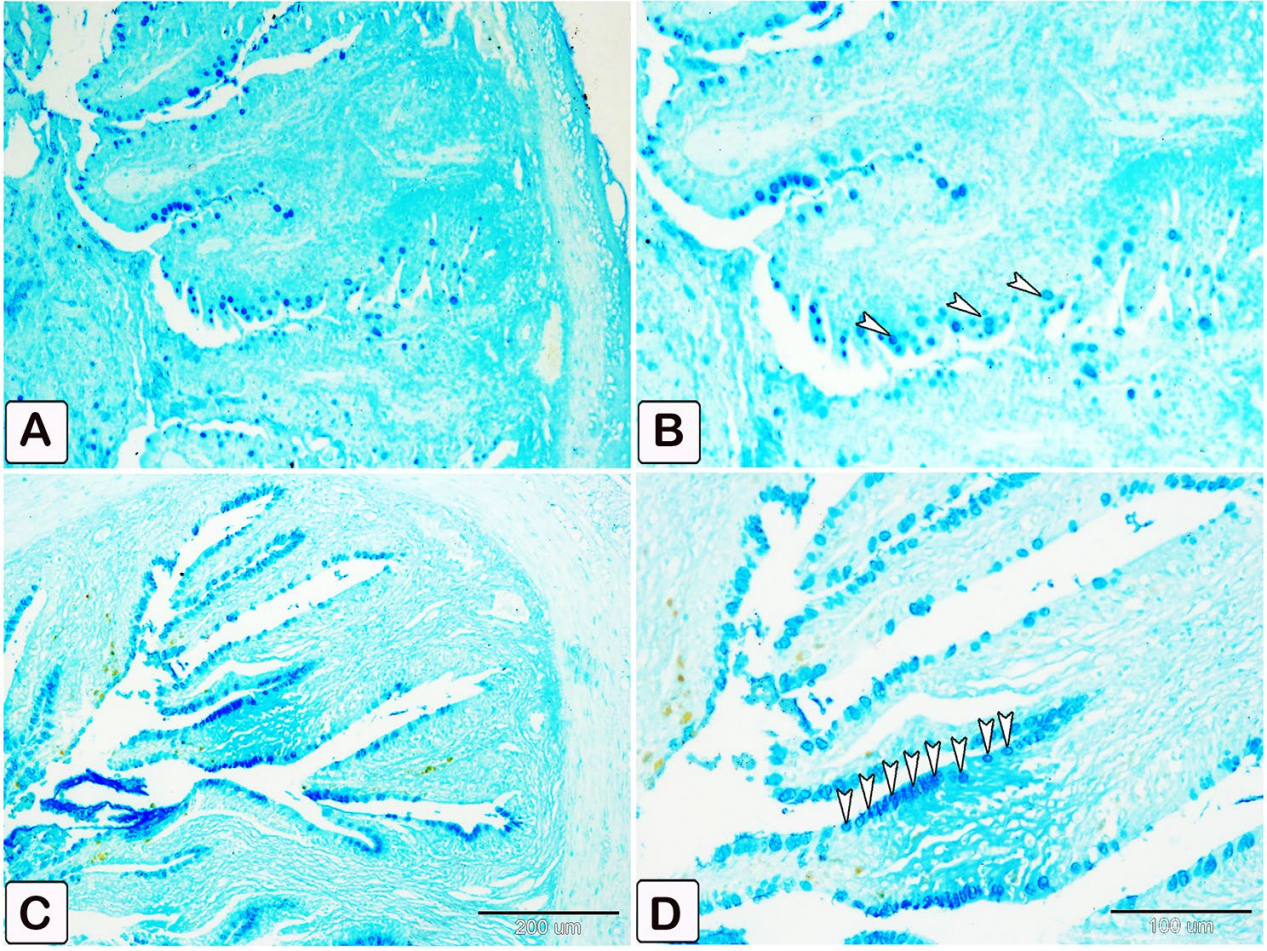
Transverse section of intestines stained with Alcian blue pH 2.5 to determine the difference of acidic secretion in the goblet cells between control and infected fish. (**A** and **B**) Paraffin section of negative control intestine showing only a few goblet cells with blue staining of acidic secretions (arrowheads). (**C** and **D**) Paraffin section of the intestine of infected fish shows a statistically significant increase (*P* = 0.016) in the number of goblet cells with extensive acidic secretion compared to the control (arrowheads)

**Fig. 5:**
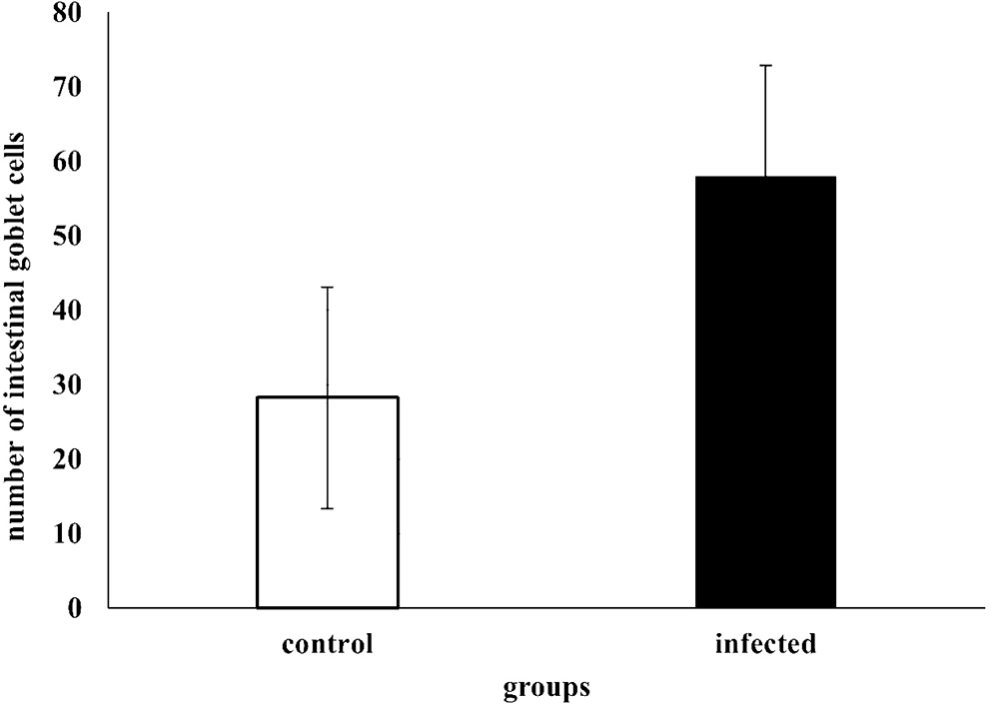
Quantitative analysis of the number of goblet cells in the mucosa of intestine. Results showing significant increase in the number of mucosal goblet cell in the infected fish (58 ± 27.4) compared to control (28.25 ± 14.2)

The number of goblet cells had increased /in the parasitic infected group, as shown in semithin intestinal mucosa stained with toluidine blue (Fig. 3A and B). Whereas many of the goblet cells in parasite infested group, presented acidic secretion as demonstrated by Alcian blue pH 2.5 (Fig. 4A-D). Quantitative analysis showed a statistically significant increase (*P* = 0.16) in the number of goblet cells in the intestinal mucosa of infected fish (58 ± 27.4) compared to control fish (28.25 ± 14.23) (Fig. 5).

### Mast cells

**Fig. 6:**
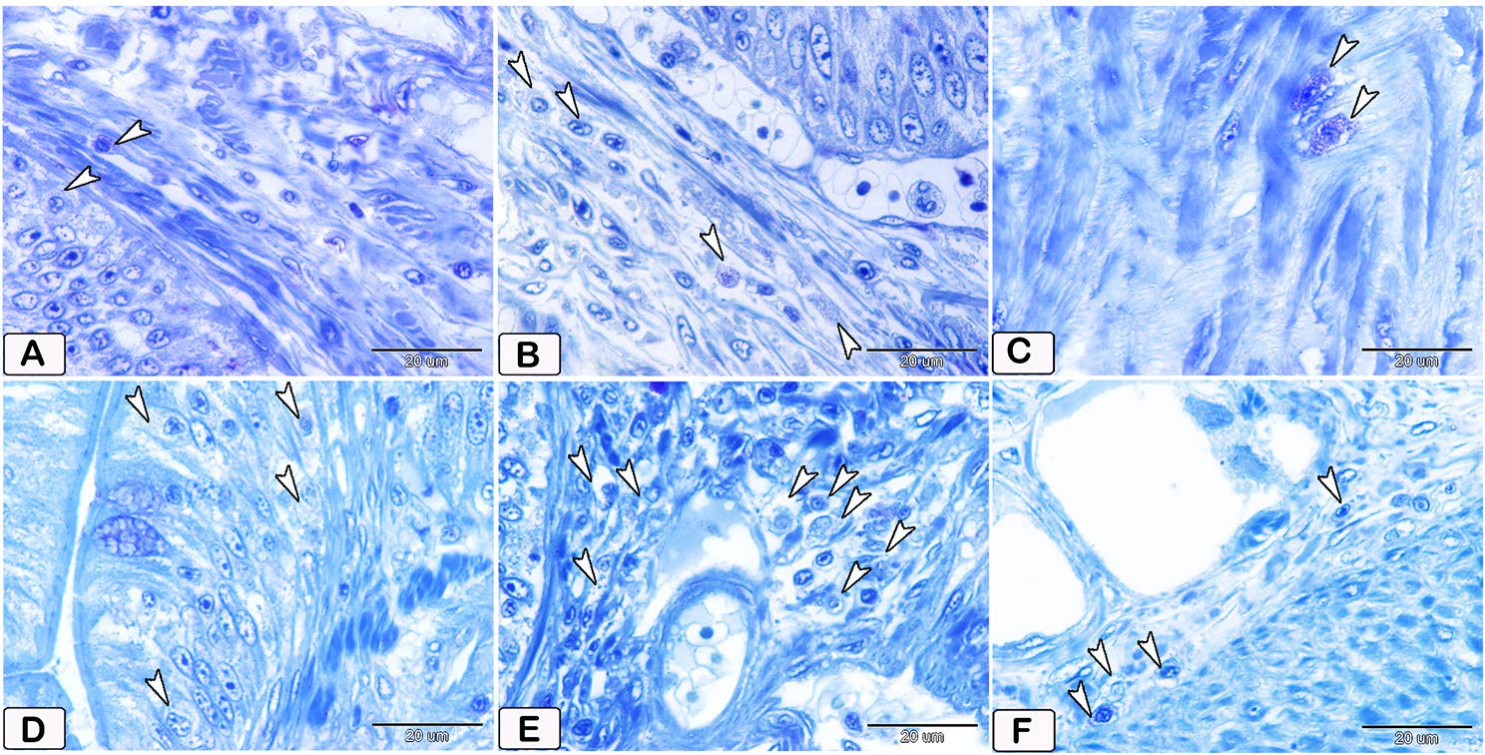
Semithin section of the intestines of African catfish *C. gariepinus* stained with toluidine blue reveals a notable disparity in the number of mast cells between the control and the infected fish. (**A**, **B**, and **C**) Intestine of control fish demonstrates a low number of mast cells in the columnar epithelium and connective tissue (arrowheads). In Figure B, it is evident that blood arteries are encompassed by elongated mast cells. Additionally, distinct metachromatic granules of mast cells were seen. (**D**, **E**, and **F**) Intestine of infected fish demonstrates a higher abundance of mast cells in the columnar epithelium and connective tissue (arrowheads). Cellular degranulation was observed

In the present study, mast cells (Fig. 6) are characterized by their mononuclear, oval, or irregular morphology. Mast cells, which are immune cells, are found on the surfaces of mucous membranes. There are two types of cells: one is situated between the epithelial cells(Fig. 6A), while the other is present in connective tissues(Fig. 6. B). Cells tend to aggregate near blood vessels, where they exhibit greater elongation. Mast cells possess highly powerful granule proteins (Fig. 6C).

Metachromatic staining with toluidine blue is commonly employed for the detection of mast cells (Fig. 6C). Under light microscopy, mast cells with granular cytoplasm were observed, exhibiting distinctive metachromatic granules (Fig. 6C). Our findings demonstrate an augmentation in the quantity of mast cells at the infection site, as well as the activation of these mast cells(Fig. 6D, E, F).

**Fig. 7:**
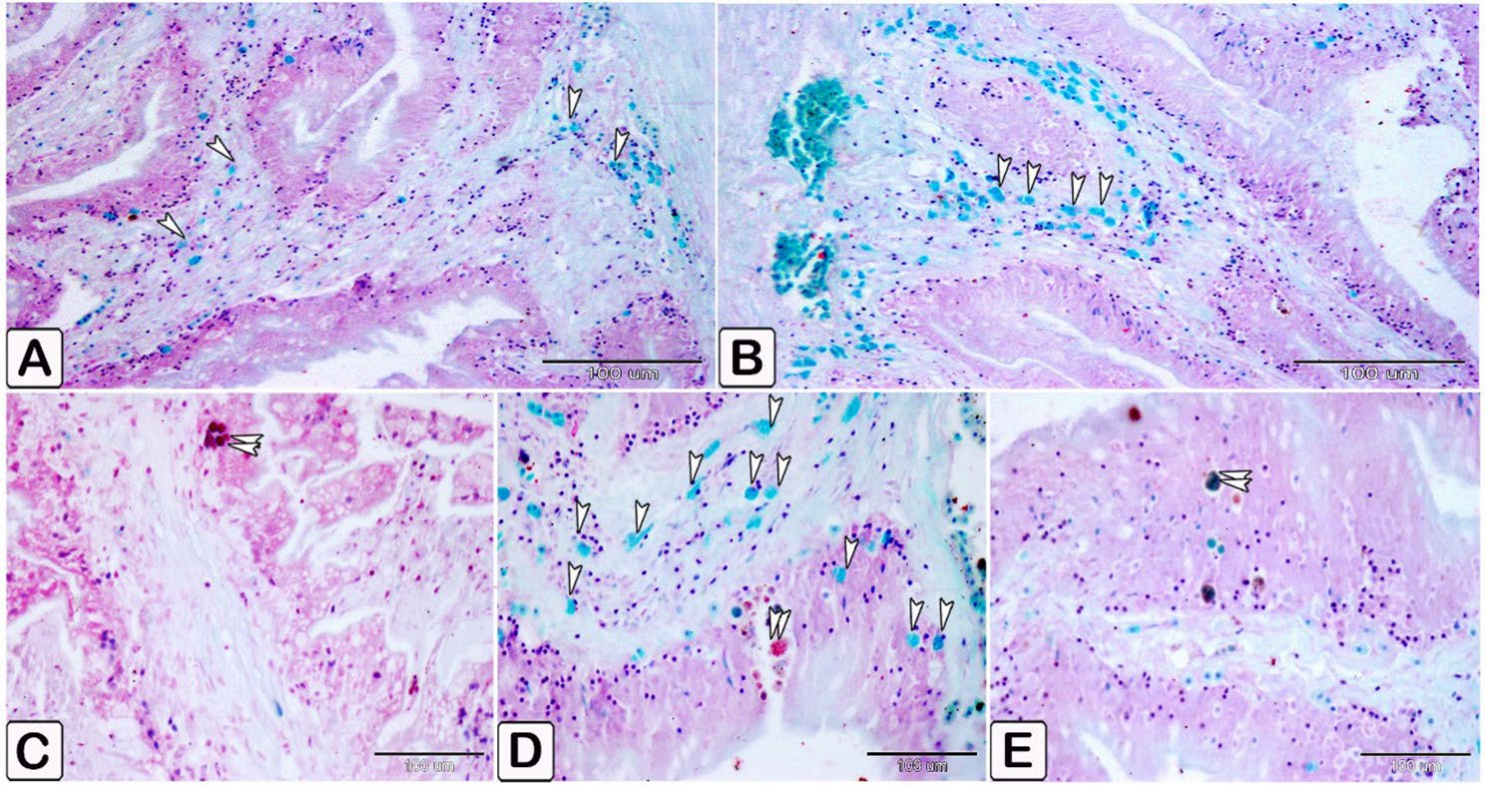
Paraffin sections illustrating distribution of intestinal mast cells in control fish, stained with alcian blue PH2.5/safranin O. Mast cells displayed as red and blue granules. (**A** and **B**) Distribution of mast cells within the blood vessels, with most cells exhibiting blue-colored granules. (**C** and **D**) Distribution of mast cells within the epithelium, characterized by red granules (double arrowheads). (**E**) mast cells containing a mixture of blue and red granules within its cells, characterized by their blue granules (arrowheads)

**Fig. 8:**
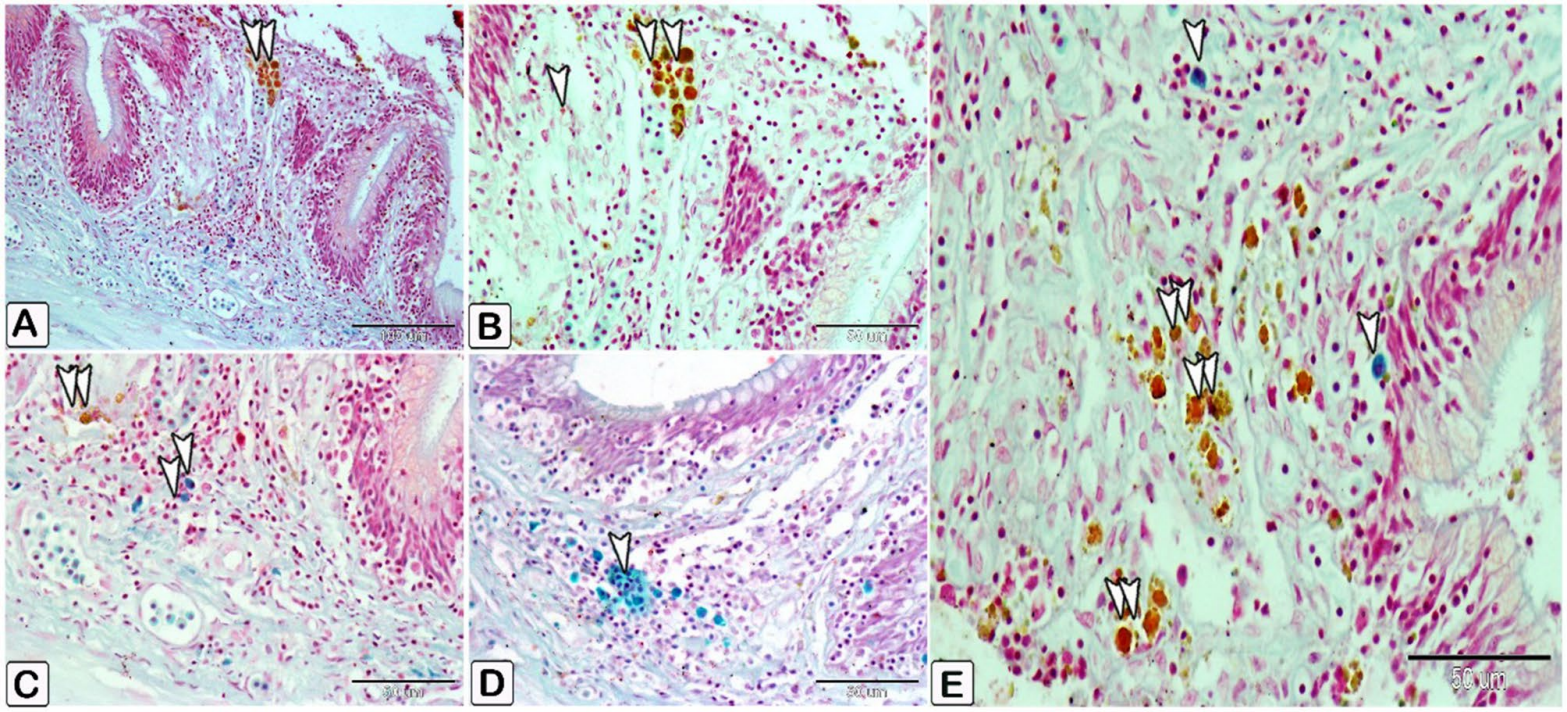
Paraffin sections illustrating distribution of intestinal mast cells in infected fish, stained with alcian blue PH2.5/safranin O. (**A-E**) Mast cells (double arrowheads) contain yellowish granules can be observed. The mast cells are distinguished by their blue granules (arrowheads)

**Fig. 9:**
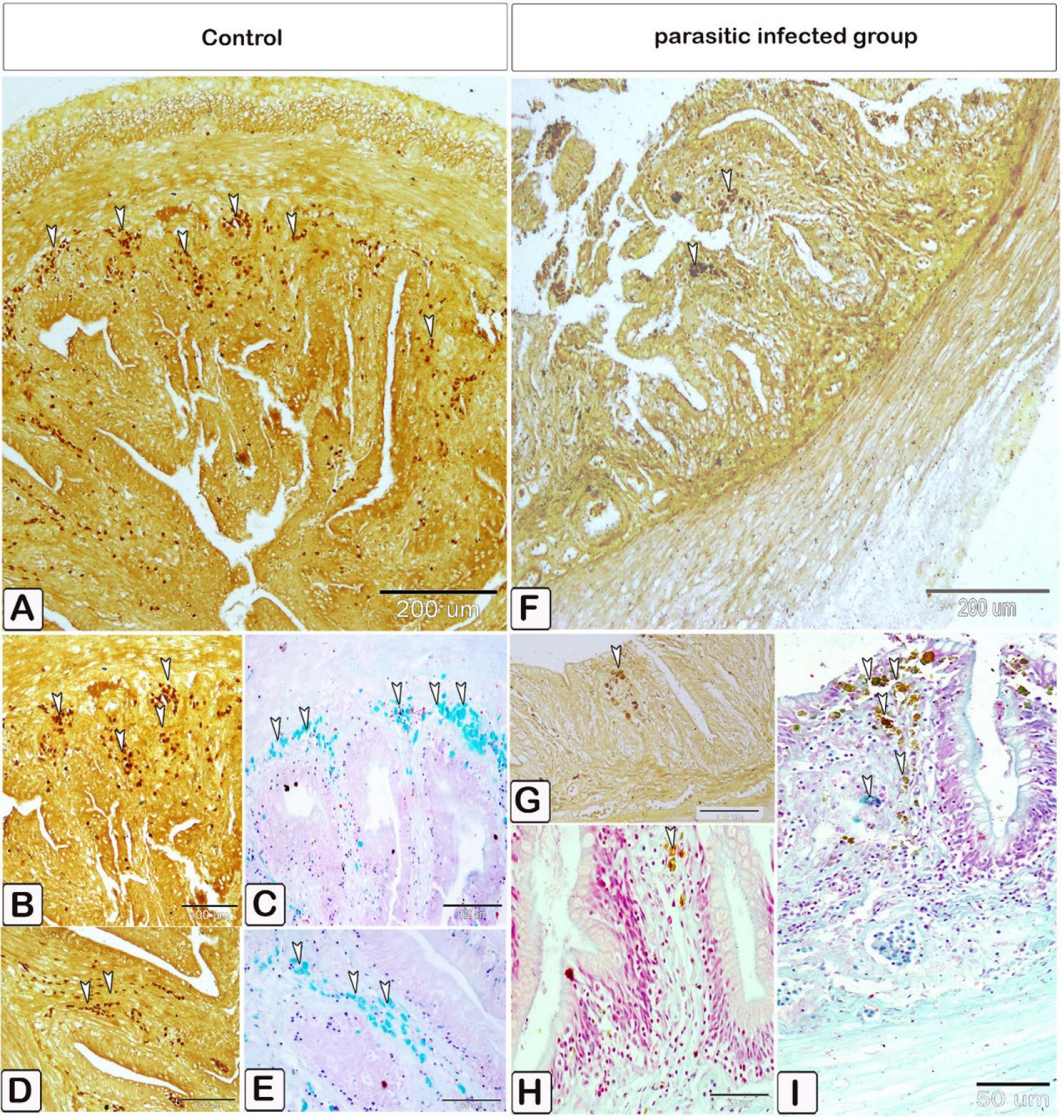
Paraffin sections illustrating distribution of intestinal mast cells in control and infected fish, stained with silver impregnation methods and alcian blue PH2.5/safranin O. The staining demonstrated the mast cells’ sensitivity to the silver dye in the control and infected fish. The stained figures using Alcian Blue pH 2.5 with Safranin were included to verify the distribution of positive cells stained with silver stain. The mast cells, characterized by their blue granules (arrowheads). (**A**-**E**) Transverse section of the intestine in control fish exhibited mast cells (arrowheads), had a brown coloration positive to silver dye (**A, B**, and **D**). (**C** and **E**) the comparative figures B and D confirm mast cells stained with alcian blue pH2.5 and safranin O, where they displayed with a blue stain. (**F-I**) Transverse section of the intestine of infected fish exhibited mast cells (arrowheads), having a positive brown coloration to silver dye (**F** and **G**), (**H** and **I**) the comparative figures B and D confirm mast cells stained with alcian blue pH2.5 and safranin O, where they displayed a blue coloration (arrowheads)

The differentiation of mast cells between the control and infected fish was studied by utilizing a specific stain; s Alcian blue pH 2.5 with safranin O, which allows for the distinction of variations in the affinity of stains for mast cell granules. Most of the mast cells in the control exhibited blue staining granules (Fig. 7A and B, with only a few cells showing red staining granules (Fig. 7C-E). Conversely, in the infected fish, most mast cells displayed yellowish granules, as shown in Fig. 8. Through the utilization of silver impregnation techniques, the labelling demonstrated that both groups of mast cells were responsive to the silver dye. To further evaluate that the cells show a positive response to silver stain (Fig. 8). This was confirmed with the results of Alcian Blue pH 2.5 and safranin O, where mast cells are distinguished by their blue granules, as shown in Fig. 9.

**Fig. 10:**
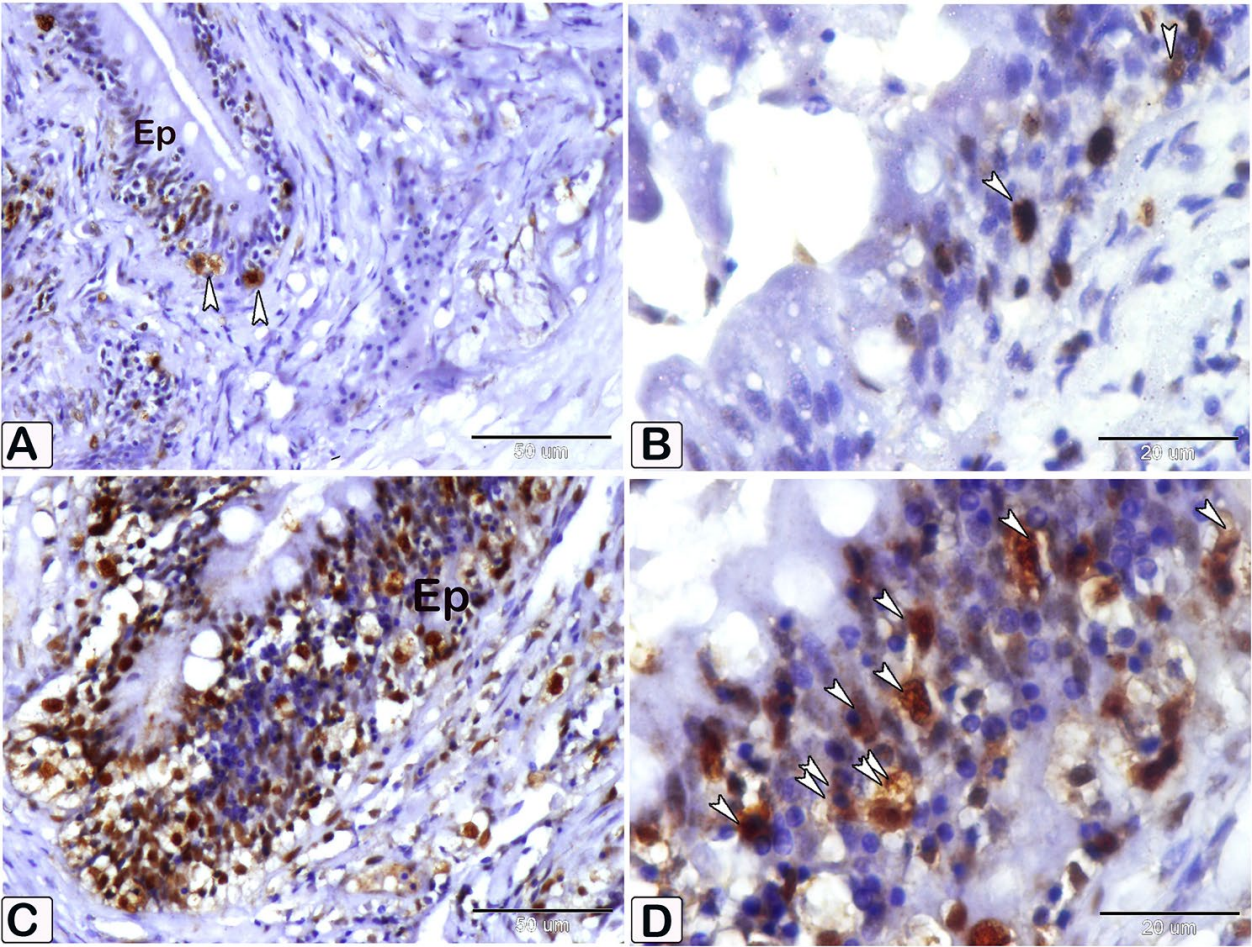
An immunohistochemical staining of anti-PCNA in the intestines of control and infected fish showing the significant difference in the number of mast cells within the epithelium (Ep) between the control and infected fish. (**A** and **B**) The intestine of control fish shows minimal immunoreactivity of PCNA (arrowheads). (**C** and **D**) The intestine of infected fish shows higher immunoreactivity of PCNA (arrowheads). The degranulation of mast cells is observed (double arrowheads)

**Fig. 11:**
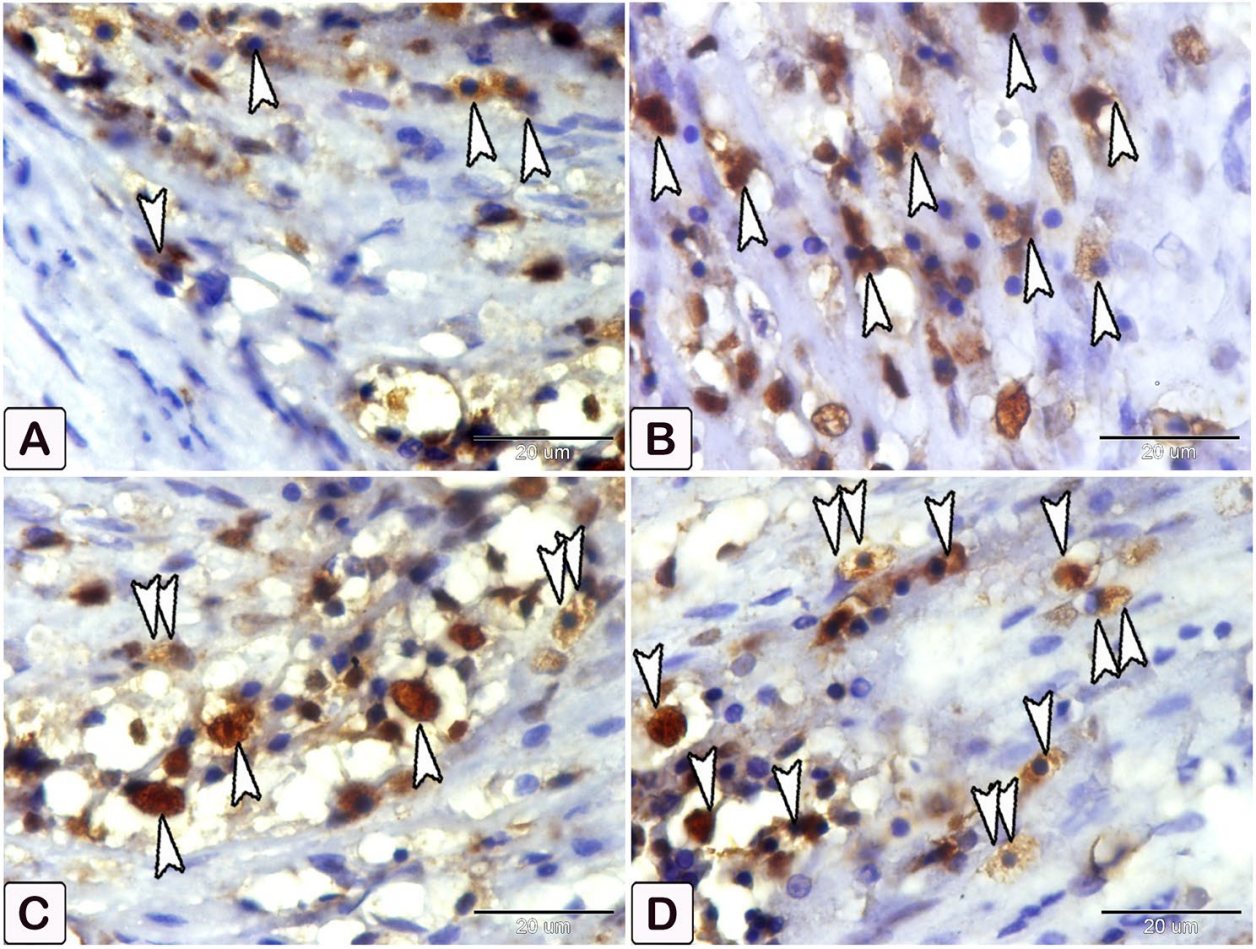
An immunohistochemical staining of anti-PCNA in the intestines of control and infected fish showing the significant difference in the number of mast cells within the blood vessels between the control and infected fish. (**A**) The intestine of control fish shows minimal immunoreactivity of PCNA (arrowheads). (**B, C**, and **D**) The intestine of infected fish shows higher immunoreactivity of PCNA (arrowheads). The degranulation of mast cells is observed (double arrowheads)

**Fig. 12:**
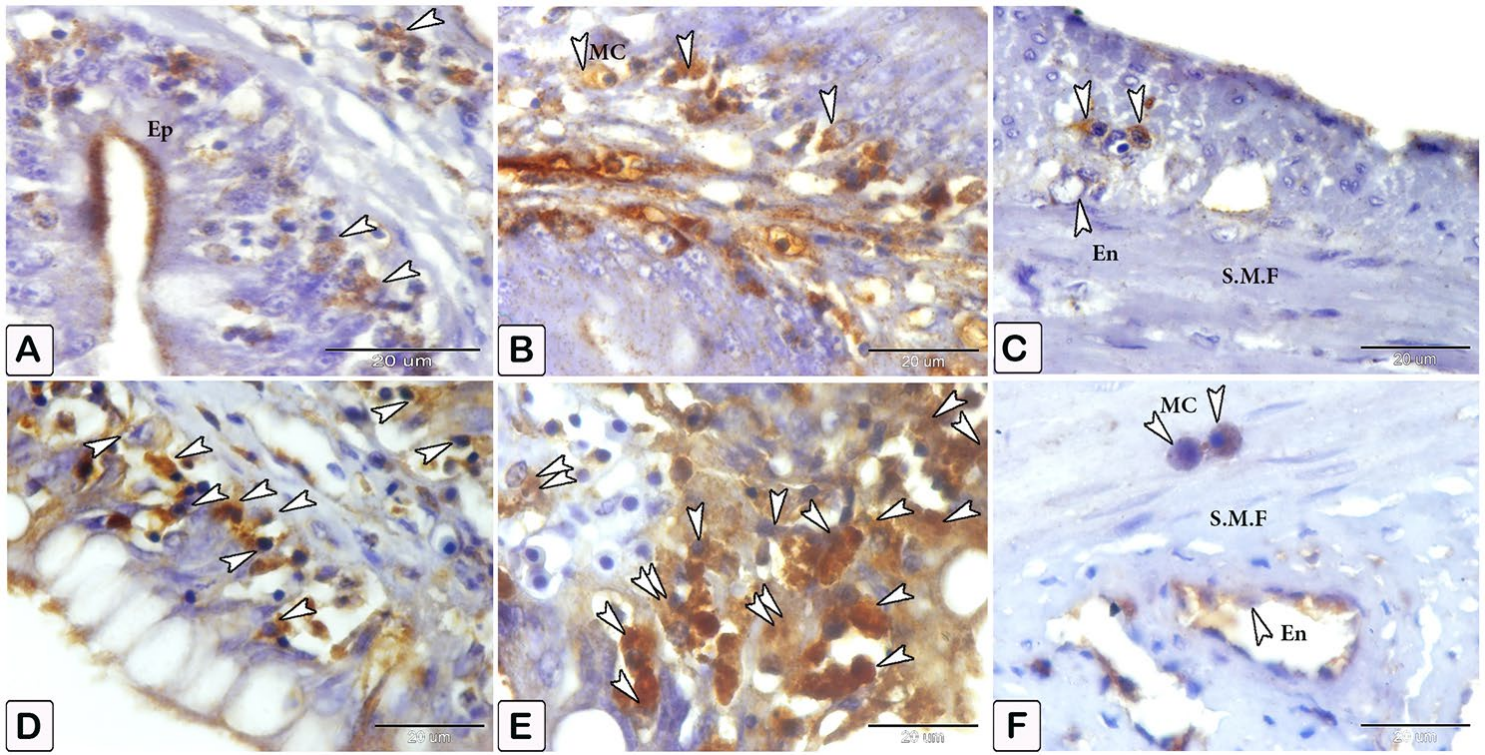
An immunohistochemical staining of anti-VEGF in the intestines of control and infected fish showing the significant difference in the number of mast cells between the control and infected fish. (**A-C**) The intestine of control fish shows minimal immunoreactivity of VEGF (arrowheads), in figure C, specifically the interaction in mast cells between smooth muscle fibers (SMF) and the mild reactivity of the endothelium (En). (**D-F**) The intestine of infected fish shows numerous mast cells (arrowheads). The degranulation of mast cells is observed (double arrowheads), in figure F, the response of mast cells is observed, particularly the significant increase in the size of mast cells between smooth muscle fibers (SMF) and extensive responsiveness in endothelium compared to the control

**Fig. 13:**
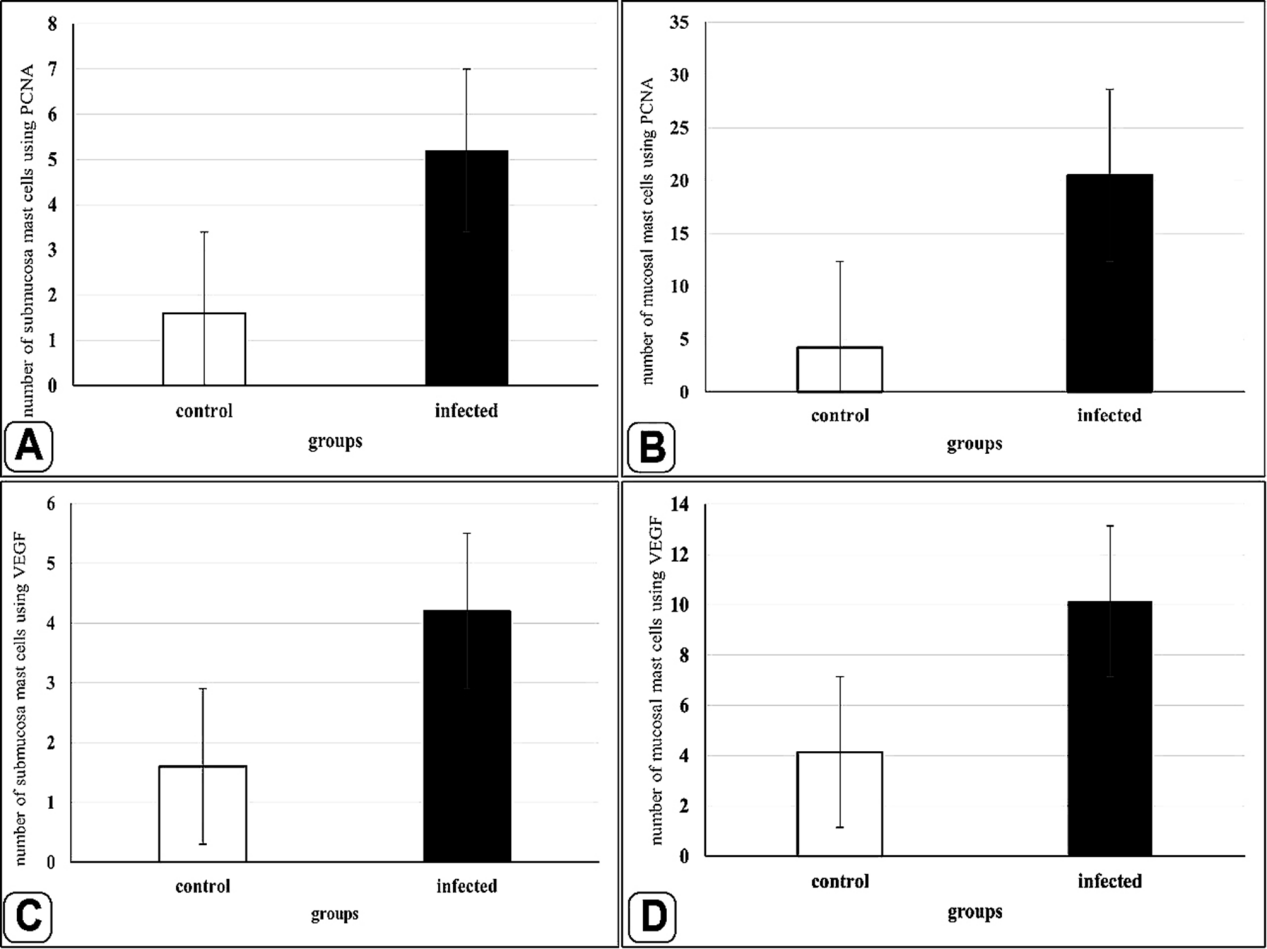
Histogram shows a statistically significant increase in the number of mast cells in the submucosa and mucosa of intestine, immunopositively to PCNA and VEGF in the control and infected fish

Interestingly, the intestinal mast cells were immunopositive to anti-PCNA and anti-VEGF (Figs. 10, 11 and 12). The immunohistochemical results showed a notable elevation in the expression of PCNA-positive cells within the epithelium and blood vessels (Figs. 10 and 11), and VEGF genes (as illustrated in Fig. 12) within the infected fish, compared to the control. A significant increase in cell size was seen in the intestine of infected fish, associated with the degranulation of cells. Additionally, there was a significant increase in the number of mast cells in the submucosa and mucosa layer of infected fish compared to control using anti-PCNA (Fig. 13A and B) and anti-VEGF (Fig. 13C and D).

### Eosinophilic granular cells

**Fig. 14:**
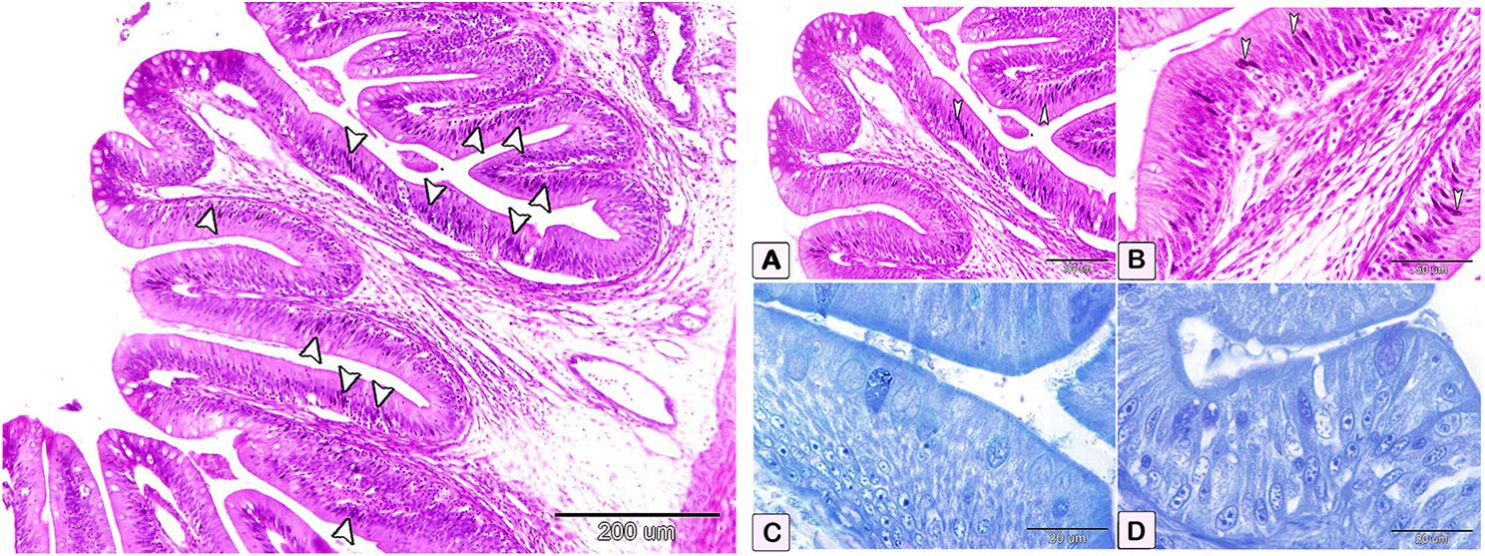
A: A paraffin section of the intestines showing distribution of eosinophile granular cells; B: (**A**) paraffin section of the Intestine stained with hematoxylin and eosin shows eosinophile granular cells at various levels within the epithelium (arrowheads). (**B**) High magnification shows cup-like granular cells having round nucleus, consisting of an apical zone that contains many granules. Granules had a strong positive response to the eosin stain. (**C** and **D**) A semithin section of the intestines staining with toluidine blue shows a significant difference in the quantity of eosinophilic granular cells (arrowheads) between the control (**C**) and infected fish (**D**). The observation of goblet cells (GC) within eosinophilic granular cells is noticeable

**Fig. 15:**
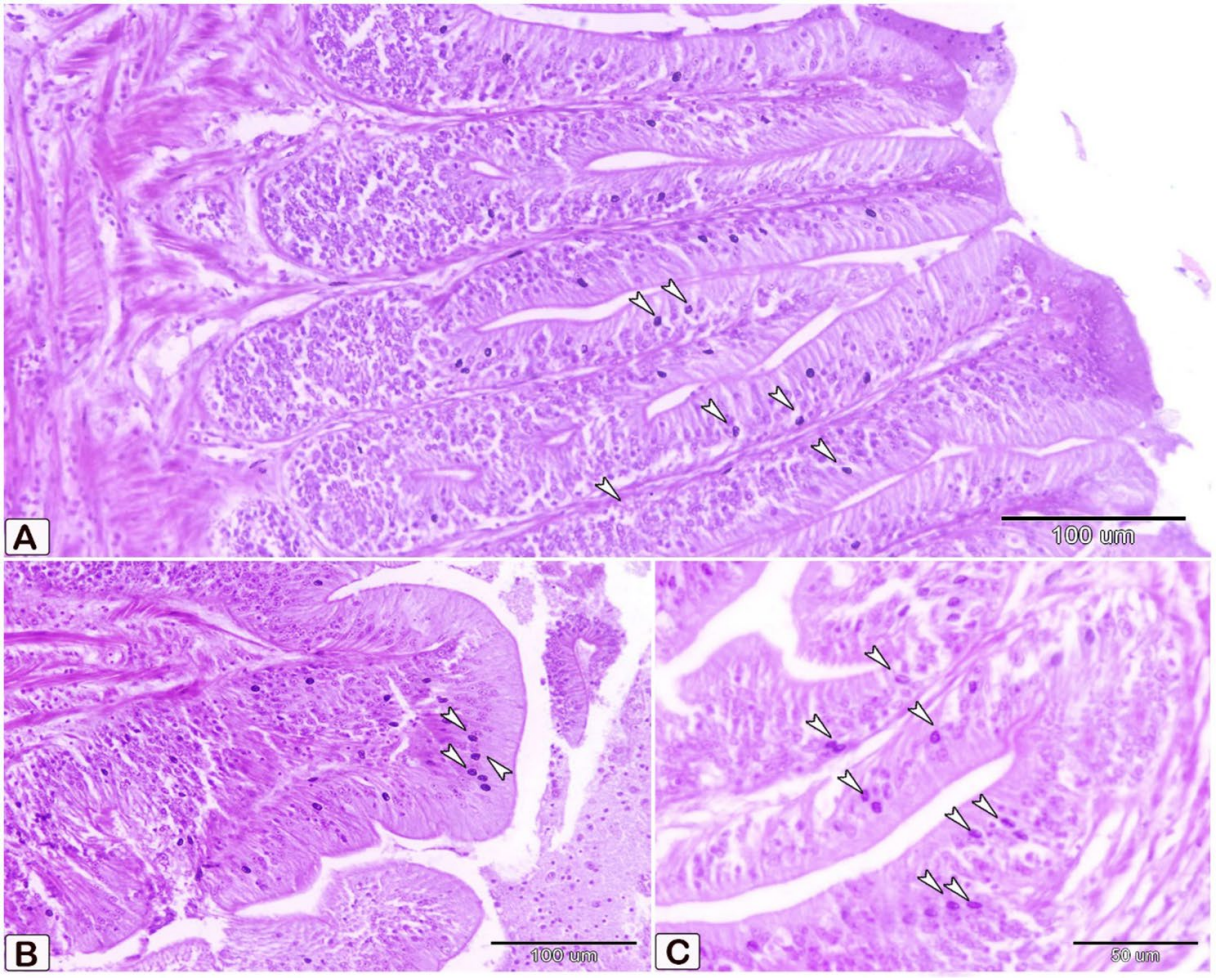
A paraffin section of the intestine of infected fish stained with PAS, revealing the presence of eosinophilic granular cells at various levels within the epithelium and purple in color (arrowheads)

**Fig. 16:**
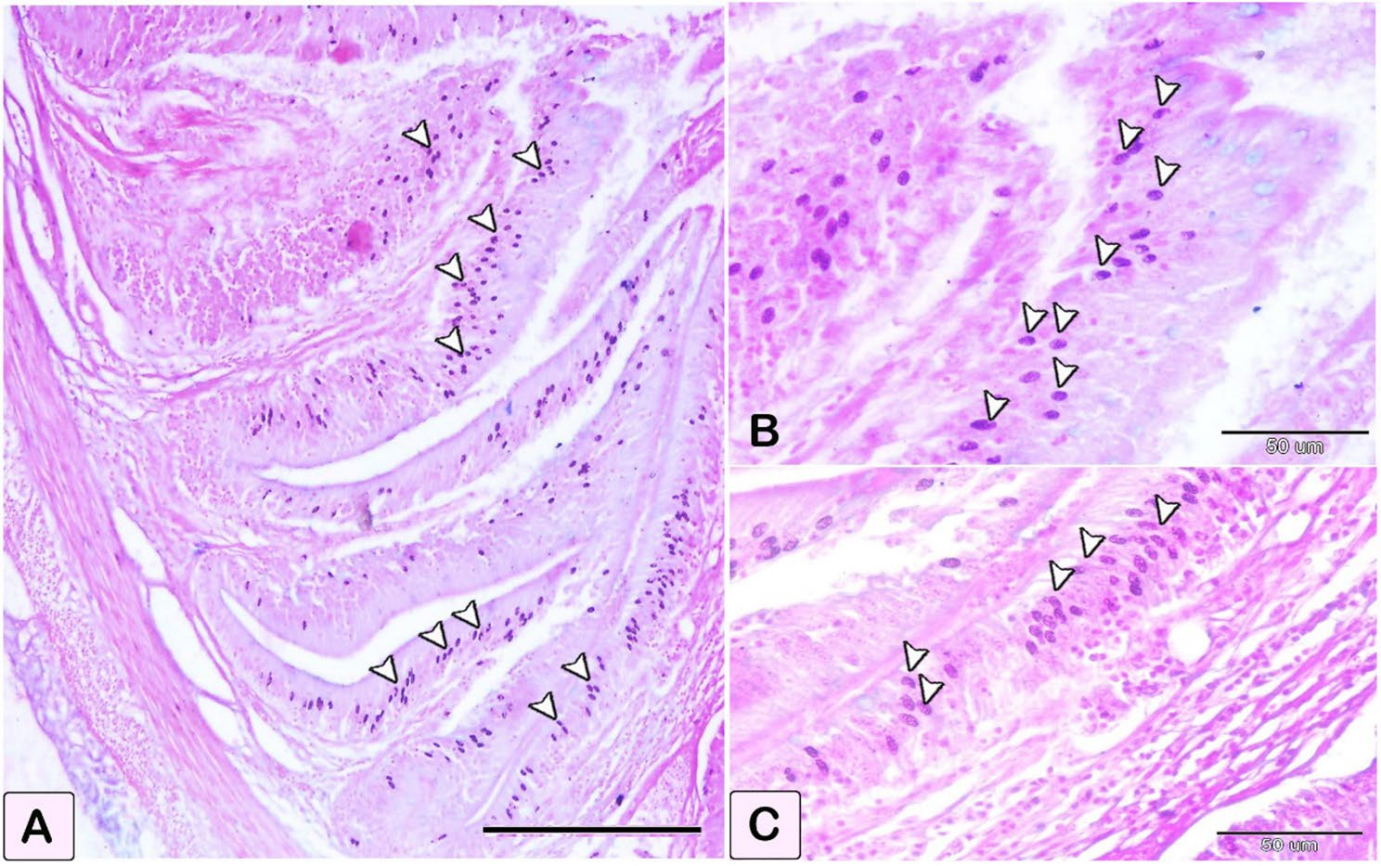
A paraffin section of the intestine of infected fish stained with alcian blue pH2.5 combined with PAS stain, revealing the presence of eosinophilic granular cells at various levels within the epithelium and stained dark purple or violet in color (arrowheads)

**Fig. 17:**
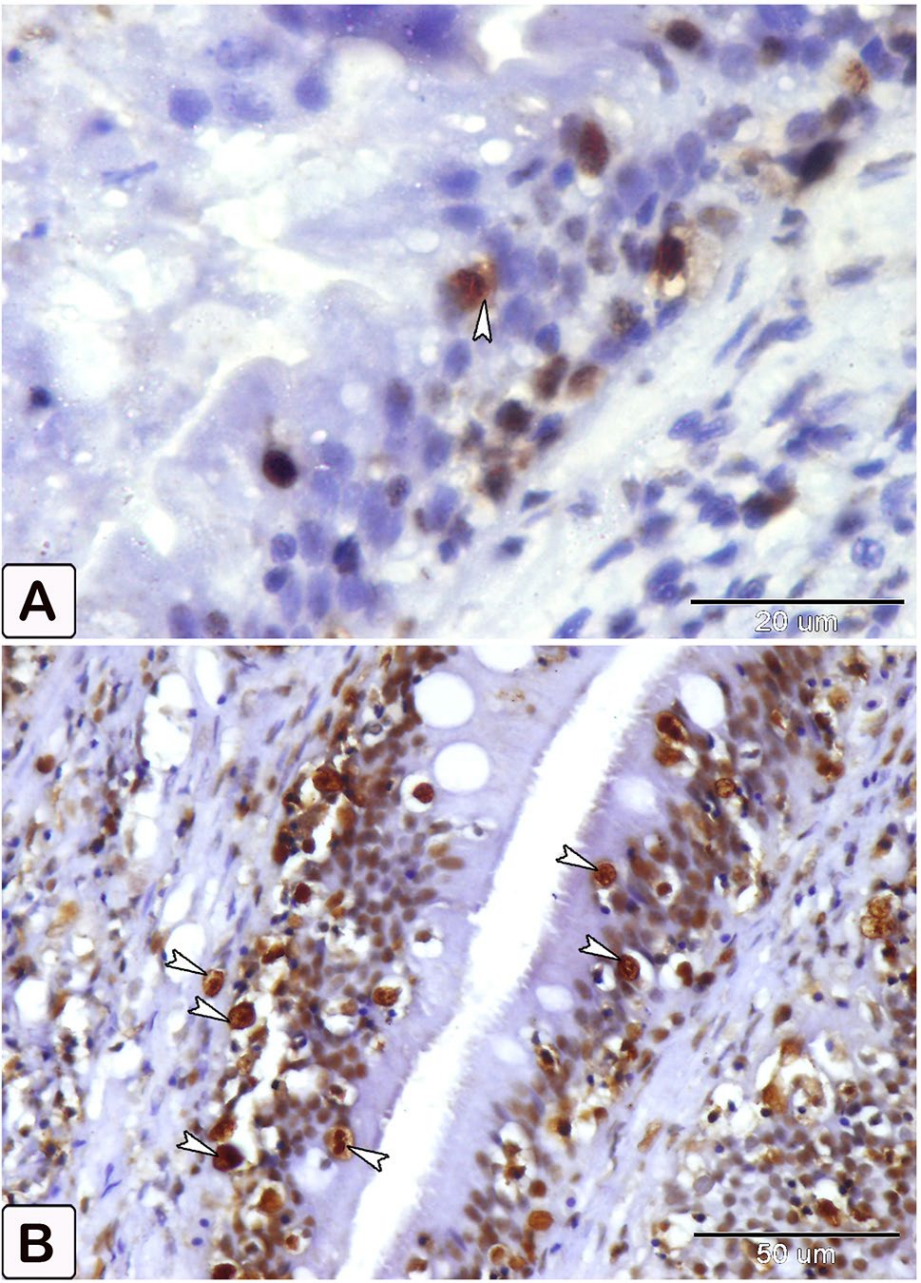
An immunohistochemical of anti-PCNA illustrates a differences in the number of eosinophilic granular cells (arrowheads) within the control (**A**) and infected fish (**B**)

Granular cells presented a cup like apical zone that contains most of the granules, and a basal portion that resembles a stem with an elongated, round nucleus. Granular cells are found on different levels of epithelium (Fig. 14a). Intestinal granular cells were strongly eosinophilic positive (Fig. 14b, A and B). These cells were close to the surrounding mucous and epithelial cells, and y frequently reach the epithelia’s apex, where they release their lumen contents (Fig. 14b, C and D). The granular cells were positive to PAS (Fig. 15), and combined alcian blue and pH 2.5 with PAS positive (Fig. 16). Immunostaining of intestinal sections with PCNA antibody, revealed a positive granular cell immunoreaction; with the parasite group having a higher number of granular cells, when compared to the control (Fig. 17).

### Dendritic reticular cells

**Fig. 18:**
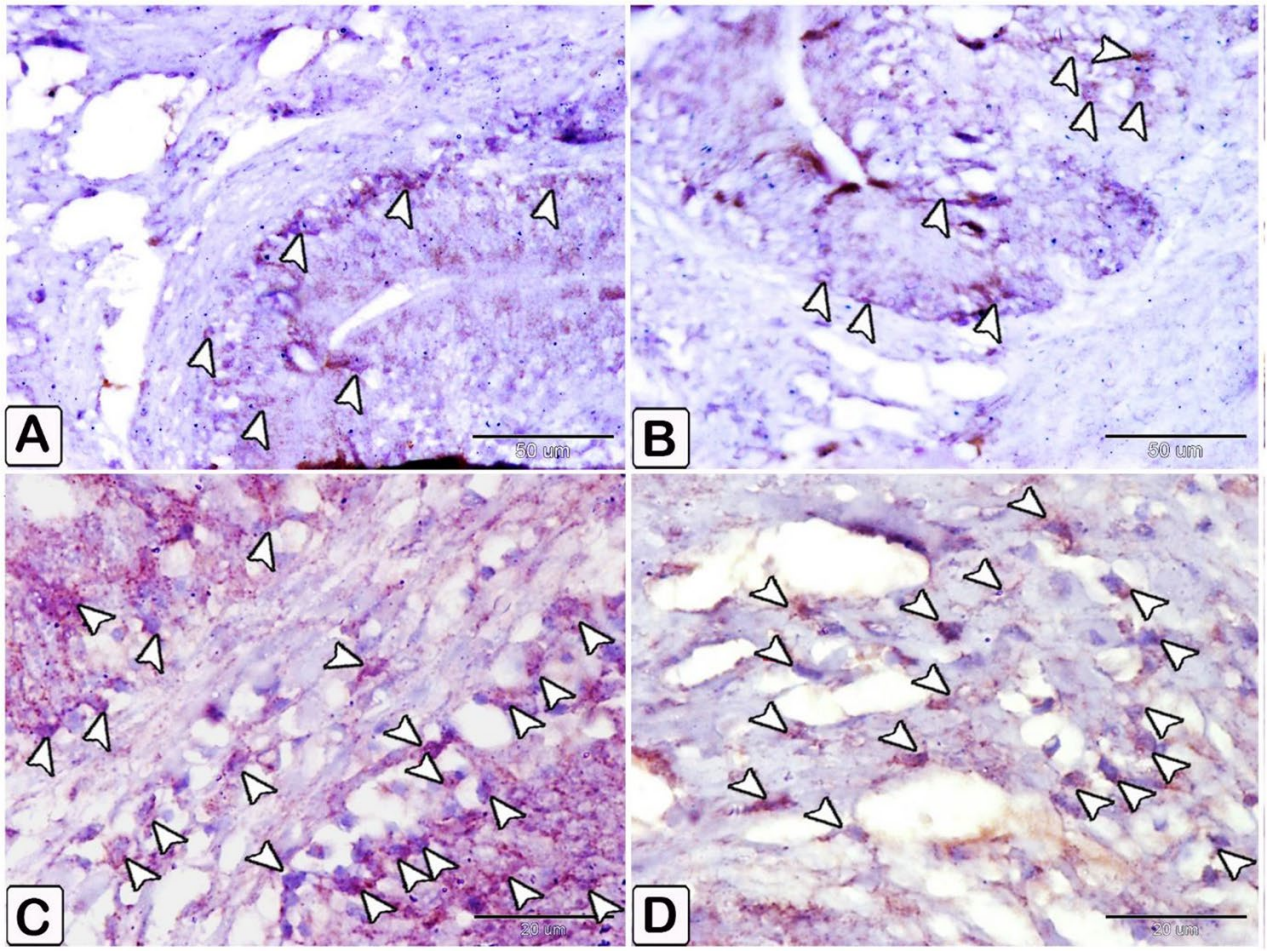
An immunohistochemical staining of anti-CD68 shows (**A** and **B**) A limited distribution of dendritic cells within the epithelium in control fish (arrowheads). (**C** and **D**) An increase in the numbers of dendritic cells in infected fish within the epithelium and lamina propria (arrowheads)

**Fig. 19:**
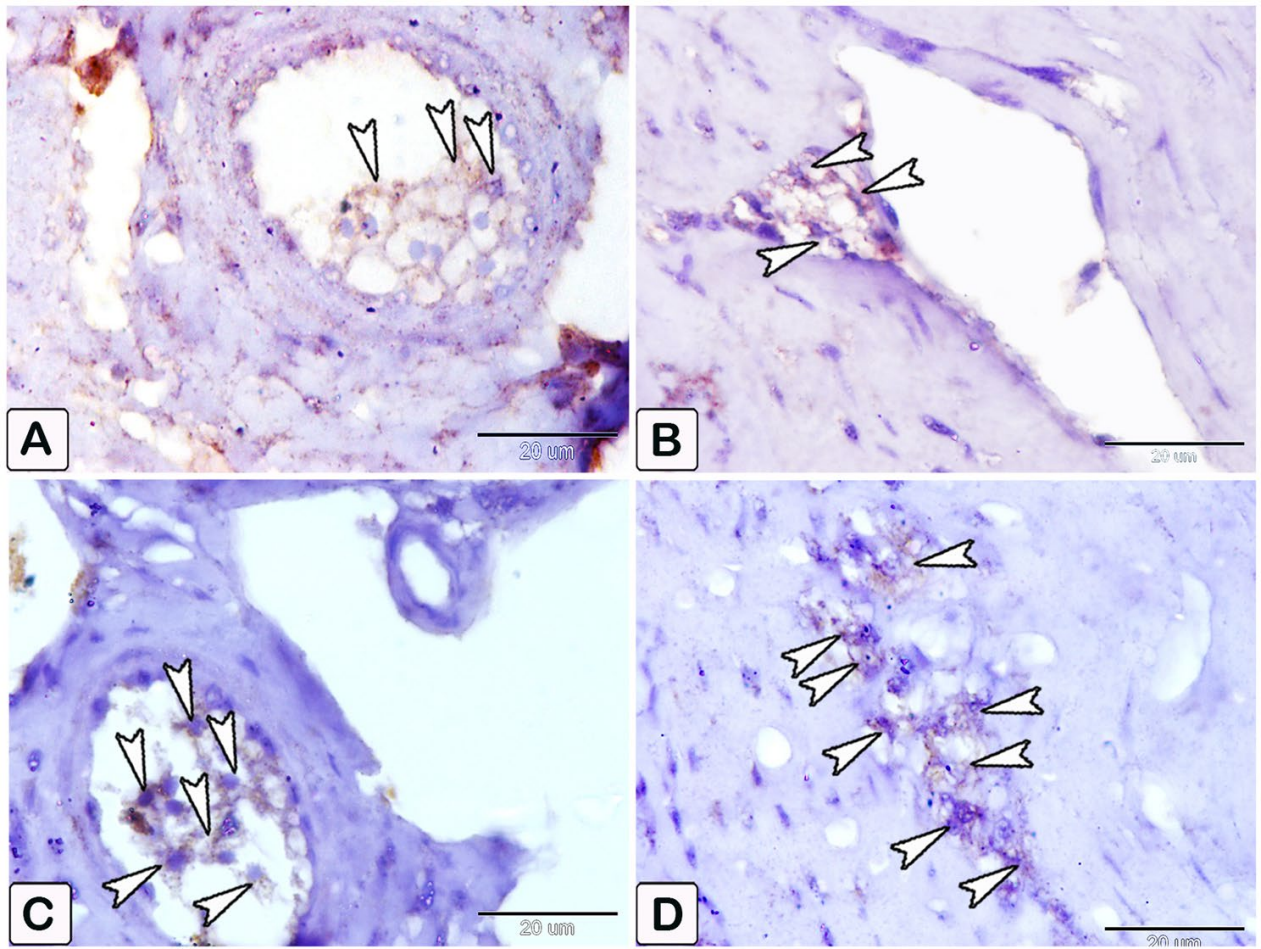
An immunohistochemical staining of anti-CD68 showing (**A** and **B**) A limited distribution of dendritic cells within the blood vessel in control fish (arrowheads). (**C** and **D**) An increase in the numbers and size of dendritic cells in infected fish (arrowheads)

**Fig. 20:**
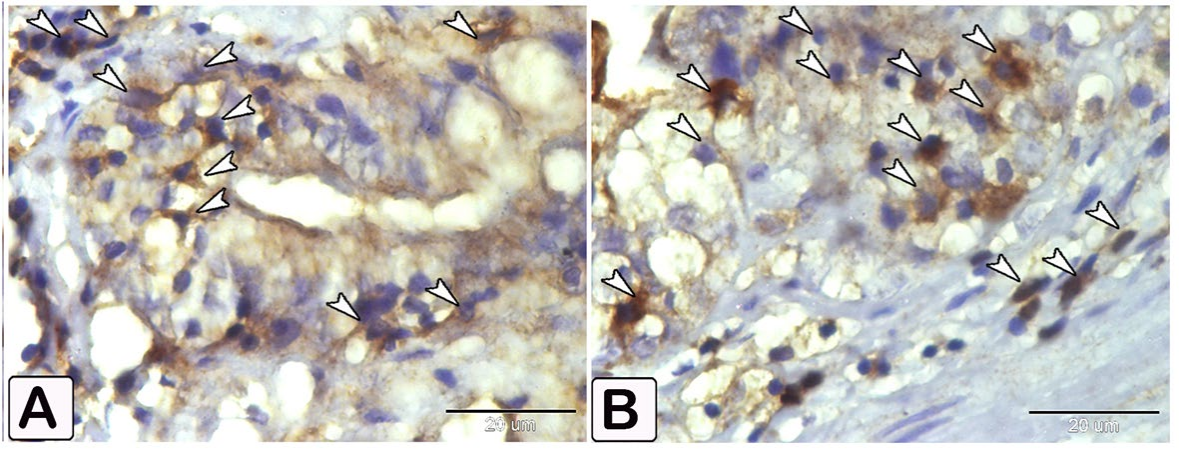
An immunohistochemical of anti-VEGF illustrates a difference in the number of dendritic reticular cells (arrowheads) within the between control (**A**) and infected fish (**B**)

Immunohistochemical labelling with anti-CD68 (Figs. 18 and 19), and VEGF (Fig. 20) demonstrated the difference in cell counts between the control and parasite groups. Cells are more abundant in the parasite group than in the control group. Dendritic cells can be seen in epithelium, blood vessels, connective tissue lamina propria, and between muscles (Figs. 18, 19 and 20). The size of dendritic cells differed between the control and parasite groups.

### Expression of anti PCNA, VEGF, and CD68

**Fig. 21:**
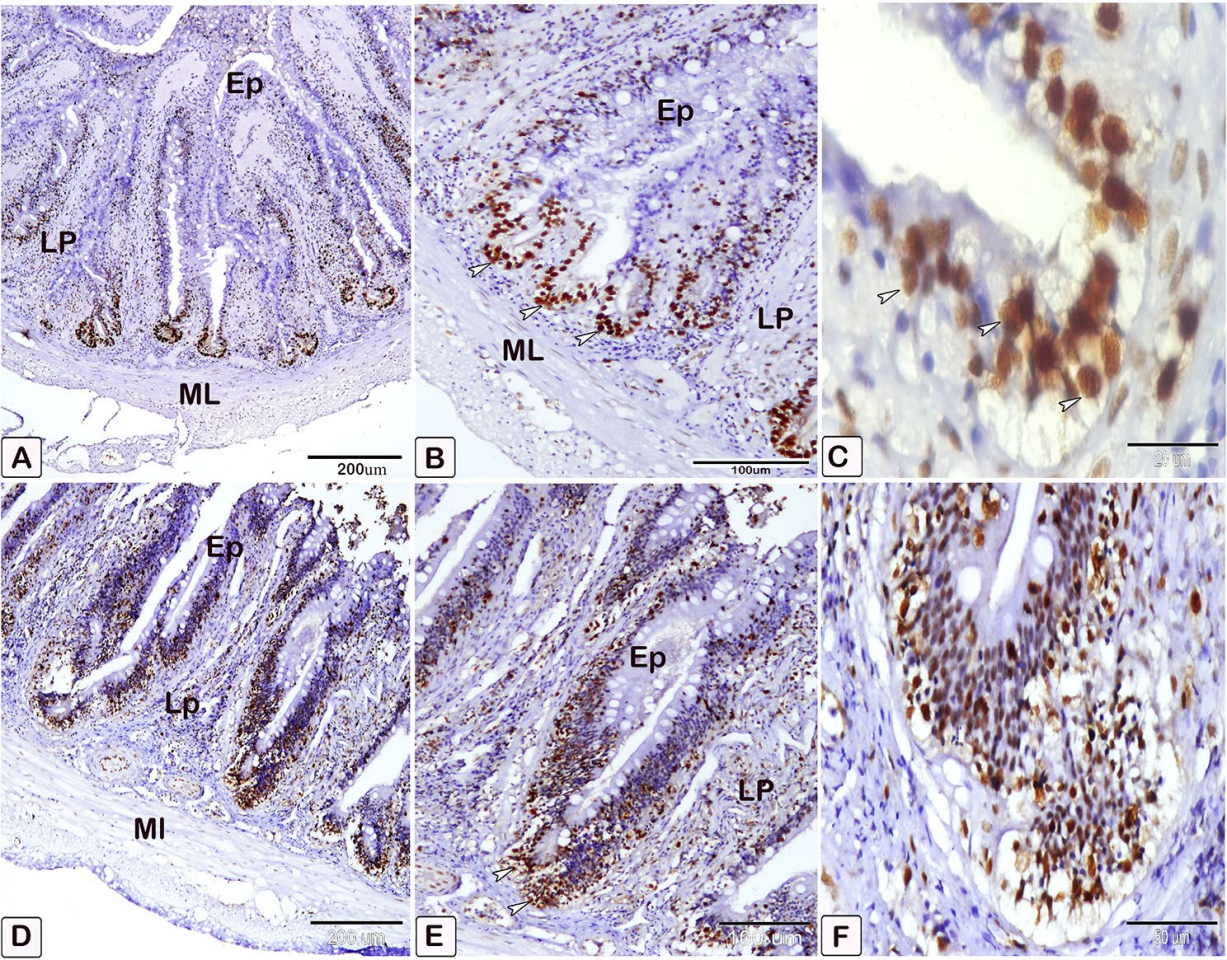
An immunohistochemical staining of anti-PCNA shows (**A-C**) Immunostaining of anti-PCNA marker observed as nuclear staining of cells lining the crypt of the intestine of control fish. (**D-F**) Immunostaining of anti-PCNA in the intestine of infected fish in epithelium (Ep), lamina propria (LP), and muscular layer (ML) (arrowheads)

**Fig. 22:**
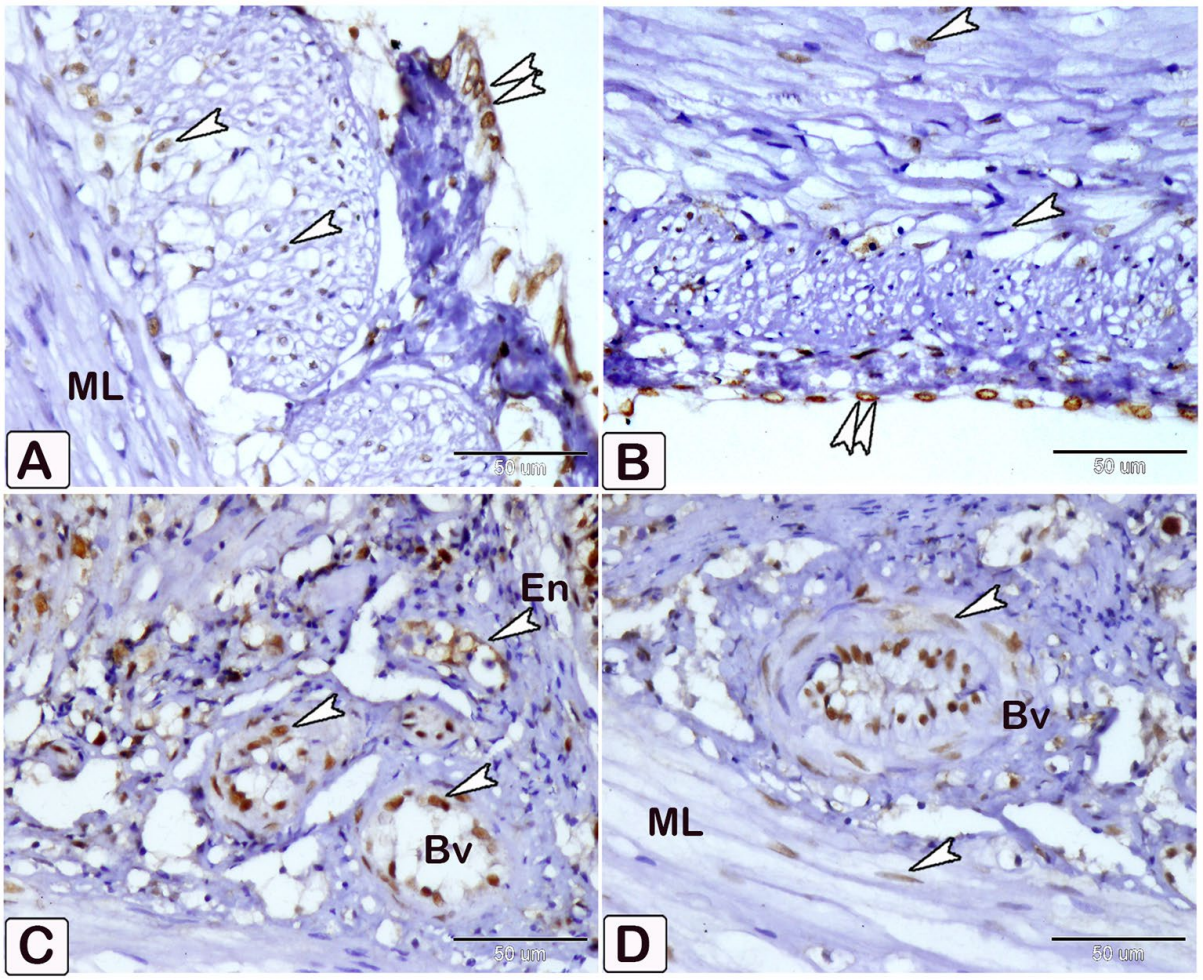
**A-D**: An immunohistochemical staining of anti-PCNA in infected fish observed in smooth muscle fiber of muscular layer (ML) and mesothelium layer, as well as significant staining of smooth muscle cells in blood vessels (Bv) at the tunica media in infected group (arrowheads)

**Fig. 23:**
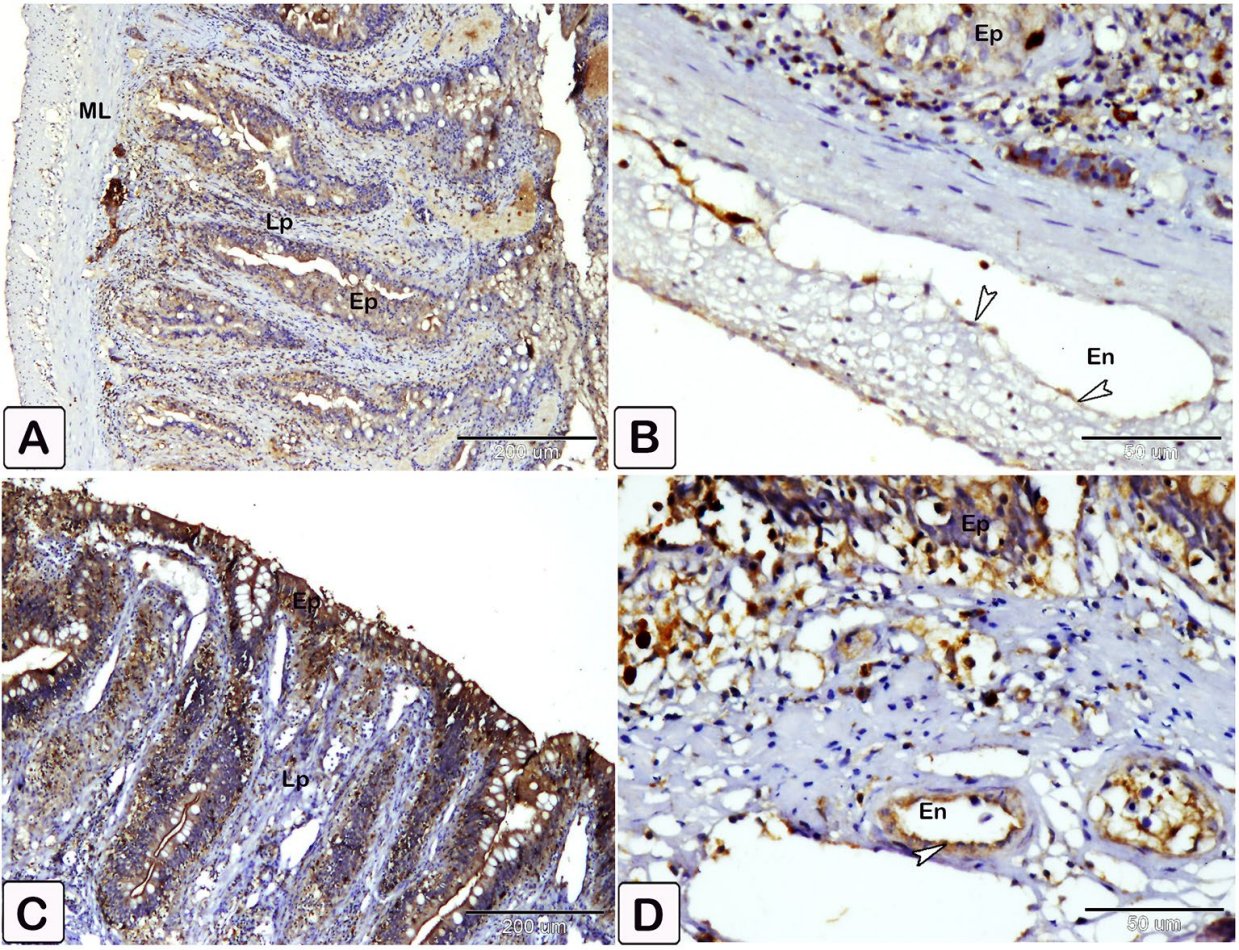
An immunohistochemical staining of the intestinal treated with an anti-VEGF marker. (**A** and **B**) Immunostaining of anti-VEGR marker was observed as staining of epithelial cells lining and the endothelium (En) of blood vessels in the intestine of control fish. (**C** and **D**) Immunostaining of anti-VEGR marker in the intestine of infected fish experienced a stronger reaction compared to control. Note the different layers of intestine are epithelium (Ep), lamina propria (Lp) and muscular layer (ML). reactions pointed by arrowheads

**Fig. 24:**
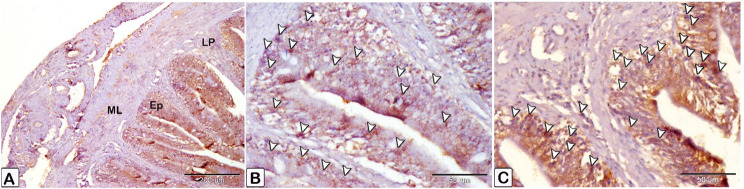
An immunohistochemical staining of the intestinal treated with an anti-CD68 marker. (**A** and **B**) Anti-CD68 marker immunostaining was observed as staining in several layers in the intestine control fish, epithelium cells exhibiting less reactivity than in the infected fish (**C**). Note the different layers of intestine are epithelium (Ep), lamina propria (Lp) and muscular layer (ML). reactions in dendritic cells pointed by arrowheads

**Fig. 25:**
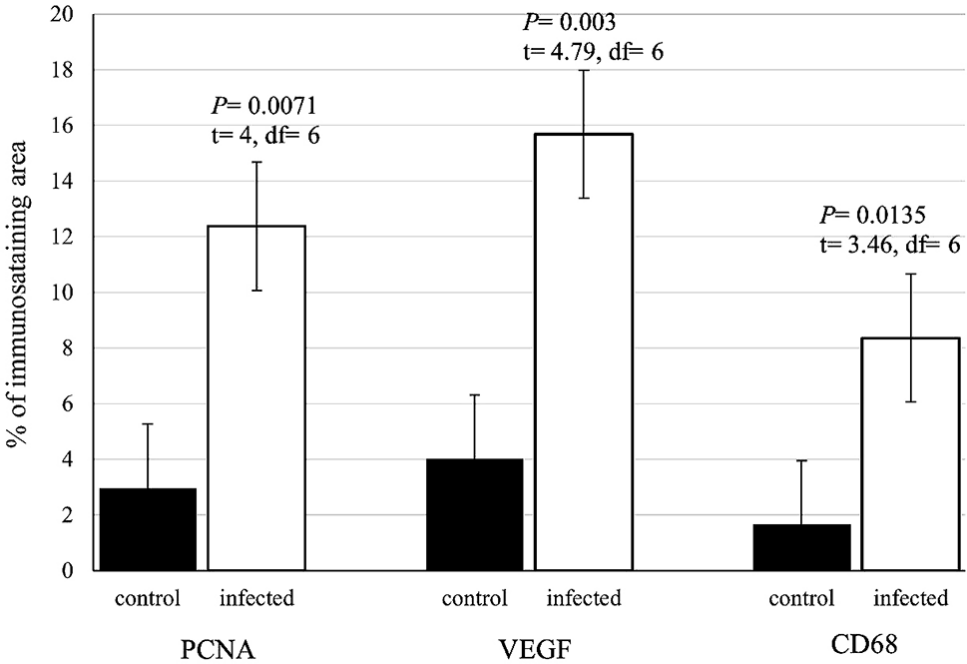
Quantitative analysis of immunostaining markers (PCNA, VEGF, CD68) showed a statistically significant difference in the intensity and percentage of positive area in the infected intestine compared to the control

Immunostaining of anti-PCNA marker was observed as nuclear staining of epithelial cells lining the crypt of the intestine. The infected fish showed a stronger reaction compared to the control (Fig. 21). The reaction was observed in smooth muscle fiber and mesothelium layer cells, as well as intense staining of smooth muscle cells in blood vessels from the tunica media group (Fig. 22). The immunostaining of anti-VEGF marker presented as strong cytoplasmic reaction in epithelium and endothelium of blood vessels in the intestine of infected fish, compared to control fish (Fig. 23). Immunostaining of anti-CD68 marker showed nuclear and cytoplasmic positive reactions in different cells of epithelium, with a stronger reaction in the intestine of infected fish than control (Fig. 24). The immunostaining markers showed a statistically significant difference in the intensity and percentage of positive area in the infected intestine compared to the control (Fig. 25).

### Histochemical reaction

**Fig. 26:**
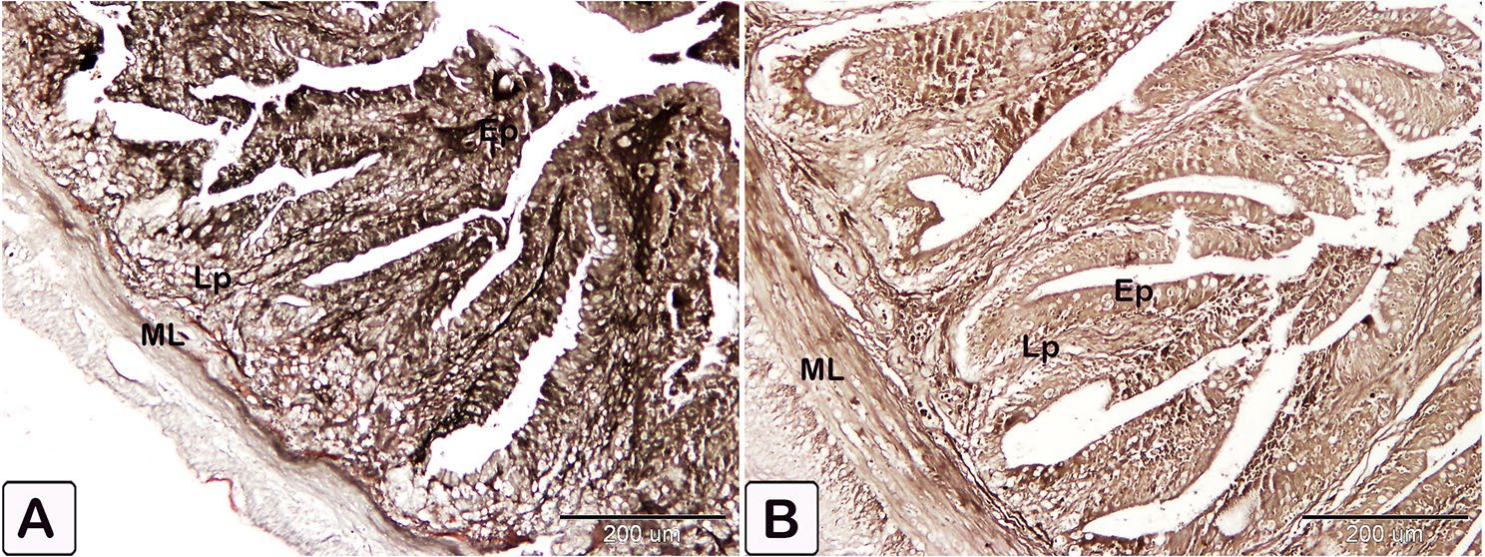
Paraffin-embedded section of intestine stained with Sudan black B demonstrates a more prominent response in the control group (**A**) compared to the infected fish (**B**). Note the different layers of intestine are epithelium (Ep), lamina propria (Lp) and muscular layer (ML)

**Fig. 27:**
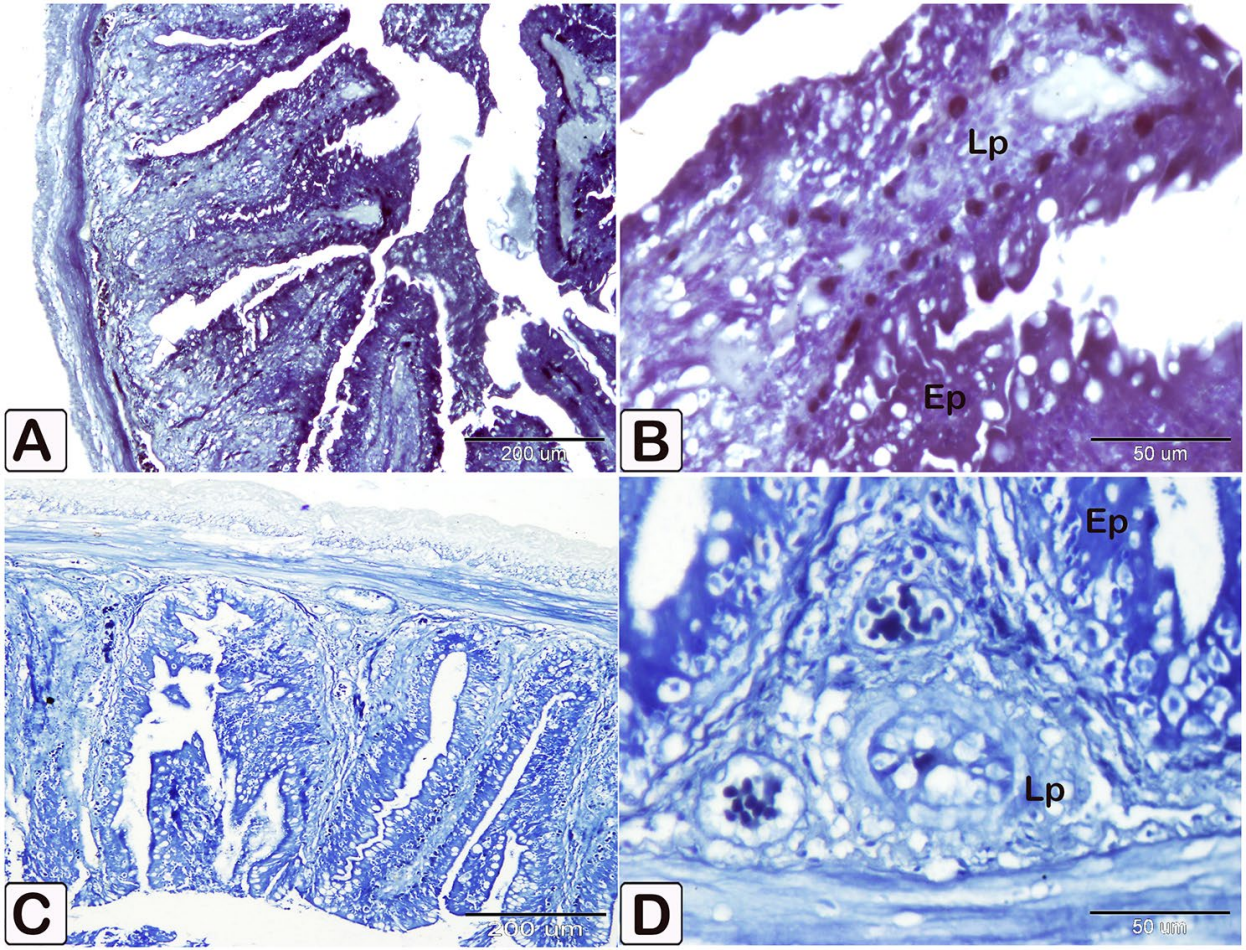
Paraffin-embedded section of intestine stained with bromophenol mercury /blue staining demonstrates a more prominent response in the control group (**A**) compared to the infected fish (**B**). Note the different layers of intestine are epithelium (Ep), lamina propria (Lp)

The evaluation of intestinal reactivity using Sudan black B (Fig. 26) and bromophenol mercury blue (Fig. 27) demonstrated a more prominent response in the infected fish compared to the control fish.

Negative from all the figures were provided in the supplementary material.

## Discussion

Most helminth parasites found in the digestive system of fish are pathogenic, thus harmful to the fish. This harm is primarily caused by the mechanical damage inflicted by the parasites’ attachment to host organs. Three types of parasitic worms were found in the intestines of *C. gariepinus* in the present study: *Polyonchobothirum clarias* and *Tetracampos ciliotheca* as cestodes, and *Procamallanus* spp. as nematodes. The prevalence rates for these parasites were 63.63%, 18%, and 18%, respectively. *Polyonchobothirum clarias* is a species commonly present in several siluroid fish inhabiting freshwater bodies in Africa. It is extensively dispersed and has been identified in Nigerian catfish Clarias lazera (*C. gariepinus*), as documented by Aderounmu and Adeniyi [41]. The tapeworms’ physical resistance to removal from the intestinal mucosa indicates that the suction generated by bothria, and the grip of the apical hooks results in significant pathogenic consequences during a severe infection. *Tetracampos ciliotheca* is a prevalent cestode parasite found in catfish across Africa [42]. The findings of our study indicated a greater susceptibility to infection in female fish (62.5%) compared to male fish (33.3%). The variations in infection rates between males and females, may be attributed to changes in feeding patterns, including variations in the amount and nutritional quality of food consumed, as well as variations in the level of resistance to infection [43].

Fish are the oldest category of vertebrates, having a unique immunological niche that encompasses all the essential elements, required for an adaptive immune response [44]. In this context, we refer to *C. gariepinus* as a model to study the primary cellular immune response against helminth parasite infections, utilizing microscopic and immunohistochemical techniques. An effective defense against parasite infections requires the ability to trigger a targeted immune response that is both sufficient and regulated, to remove the invading pathogen while minimizing injury to healthy tissues.

Mast cells are the main cells responsible for initiating allergy reactions and are believed to influence tissue remodeling, immune response, and at the microenvironment of tumors [45]. In addition, mast cells contributes to increased vascular permeability and angiogenesis in allergic disorders [46] and inflammation [47]. The findings of our study revealed that the mucosal layer presented an increase in size and quantity of mast cells due to chronic inflammation. This suggests a correlation between the level of mast cell differentiation and the use of histochemical dyes. In addition, the intestinal mast cells in the infected fish exhibited a notable rise in both reactivity and quantity when assessed with toluidine blue pH 2.5, safranin O, alcian blue pH 2.5, and silver stain. These results are consistent with the prior discovery that mast cells migrate throughout the different layers of intestinal mucosa during infection [48]. The expression and staining characteristics of mast cells are associated with the quantity and dimensions of cytoplasmic granules, as well as the maturation and differentiation of the cell [49]. The staining properties of the mast cells in the intestine, when treated with toluidine blue, varied between the connective tissue and the cells located between the epithelial layers. According to Simoes and Schoning [49], toluidine blue at pH 2.5 exhibited a strong affinity for mast cells with tiny cytoplasmic granules. Nonetheless, mast cells exhibited a non-specific response to Del Rio Hortega’s silver carbonate linked with avidin in canine mast cell tumors, surpassing the reaction observed with acidified toluidine blue [50]. Our findings showed the degranulation and activation of intestinal mast cells in the infected fish using safranine O stain. These findings aligned with the research conducted by Mokhtar et al. [26], which observed that mast cell granules, exhibit positive staining when treated with bromophenol blue and safranin O. In addition, scientists have noted the process of mast cell degranulation, the release of cytokines, and the resulting inflammatory response in teleost fish because of infection [51]. The efficacy of various histochemical dyes may be attributed to the concentration of heparin and other glycosaminoglycans, which are the main substances that interact with histochemical stains [52]. However, the preservation of intracellular glycosaminoglycans primarily relies on the fixative used, such as 10% buffer formalin [49].

Vascular endothelial growth factor (VEGF) is an evolutionarily conserved protein found in both fish and mammals. It plays crucial functions in the formation of blood vessels in mammals and the pathological expansion of blood vessels in many disorders. VEGF is produced by vascular smooth muscle cells, keratinocytes, macrophages, and other tumor cells [53]. However, alterations in PCNA resulting from cellular response place it in a crucial role in DNA replication, DNA damage, and chromatin structure and function. The present immunohistochemistry investigation revealed an increase in the expression of PCNA and VEGF in all mucosal layers of the intestine in the infected fish, with a notable emphasis on the mucosal epithelium and Lieberkühn’s crypts. The correlation between mast cells and anti-PCNA and anti-VEGF aligns with the findings on inflammatory diseases [54]. These observations can be partially attributed to hypersensitivity reactions caused by parasites, resulting in decreased oxygen and nutrient levels. The physiological regulation of VEGF overexpression is controlled by oxygen tension [55]. Kashiwakura et al. [56] proposed that the generation of IgE had a role in triggering local inflammatory responses. They also found a positive association between elevated levels of IgE and the synthesis of VEGF in mast cells. Furthermore, they observed that the increase in IgE preceded the proliferation of stem cells after infection. These findings were consistent with the research conducted by Jiménez-Andrade et al. [47], which showed that the pleiotropic effects of this cell type are linked to its ability to produce and release various lipid mediators following IgE/antigen activation.

The predominant categories for VEGF-positive stromal cells, were mast cells and macrophages [57]. Notably, the current findings demonstrated a noteworthy immunological response of mast cells in the intestines of infected fish, as indicated by the presence of anti-VEGF and anti-PCNA. This finding was corroborated in prior investigations involving mice and human mast cells [46]. Reite [58] which stated that fish mast cells share similarities with mammalian MCs in terms of their cytochemical and distribution characteristics. The current findings were consistent with these results since they demonstrate the presence of well-differentiated mast cells when exposed to anti-PCNA and anti-VEGF. This suggests that the use of avidin peroxidase linked with antibodies enhances the intensity of mast cells. This phenomenon may be explained by the elevated isoelectric point of the biotin-linked enzymes found in mast cells, or by the presence of sulfated groups in the granules of mast cells, as suggested by Piva et al. [50]. In addition, the secretory granules containing heparin and histamine promote the inflammatory response and cause the contraction of endothelial cells in venules. This contraction facilitates the movement of proteins and cells from the plasma to the connective tissue [48].

The findings from our study revealed an elevation in the expression of dendritic cells in the muscle layer, both within and outside blood vessels, lamina propria, and inter-epithelial cells in the infected fish. Data from scientific study indicates a dearth of knowledge on the immunoreactivity of dendritic cells with CD68 [59]. Additionally, there was a complete absence of information regarding immunoreactivity with VEGF. Our data indicates a significant interaction between intestinal dendritic cells and VEGF and CD68 in *C. gariepinus* when the parasite is present. The findings suggest that specific glycans derived from helminths may affect the activation of dendritic cells through C-type lectins and induce Th2-dominant reactions [7]. The findings of our study showed the activation of intestinal dendritic cells in teleost fish leads to a Th2 immune response. This activation is caused by an increase in mucous production from goblet cells, the stimulation of eosinophil granular cells, an increase in smooth muscle cell contractility mediated by VEGF, and the release of inflammatory mediators by mast cell degranulation. The observed phenomenon can be explained by the direct impact of helminth parasites, and the heightened presence of host-derived mediators known as alarmins. These alarmins are naturally released by the body and can influence the activity of dendritic cells (DCs), leading to the promotion of Th2 polarization during helminth infection [7, 60]. This study provides the initial evidence of the interaction between VEGF, PCNA, and CD68 in the immune responses of freshwater fish intestines infected with helminths.

The cells that secrete mucus had goblet like structure, and their numbers are much higher in the infected bowel compared to the healthy colon. This was determined using toluidine and alcian blue staining. This t essentially elicits a favorable response when protein staining is performed using bromophenol blue, and a comprehensive response when staining for mucopolysaccharide content was done using PAS (64). It can be confirmed in direct connection to lubrication, defensive mechanisms, maintaining osmotic responses, and promoting waste exclusion [61, 62]. Nevertheless, eosinophils have a crucial role in maintaining tissue homeostasis at the point where food interacts with the body. This is demonstrated by their capacity to regulate local immune responses and react to microbial stimuli [63]. Our investigation revealed that the intestinal epithelial granular cells of *C. gariepinus* are characterized by a cup-shaped apical zone and many cytoplasmic electron-dense granules. Accordingly, these findings could broaden our understanding of the interaction between helminth parasitic infection and host immunity, and the role of sentinel cells in the host defense.

## Conclusion

The fish’s immune system encompasses both innate and adaptive responses. The innate response is particularly crucial for promptly tackling pathogens and offering disease resistance by physical barriers, humoral factors, and cellular defenses. Additionally, fish possess a complex network of cytokines, that regulate and activate their immune system, ensuring appropriate protective responses against various pathogens. Understanding the functions of these immune cells in intestinal immunity would provide an informed strategies for controlling and preventing parasitic infections in fish, ultimately improving their health and welfare in aquaculture systems.

### Electronic supplementary material

Below is the link to the electronic supplementary material.

Supplementary Material 1

## Data Availability

All data obtained is included in this manuscript and is available on request from the corresponding authors. There is no sequence data in this manuscript.
