# Supplementary material for: Modulation of the intestinal mucosal and cell-mediated response against natural helminth infection in the African catfish Clarias gariepinus
Source: BMC Vet Res. 2024 Jul 27;20:335. doi: 10.1186/s12917-024-04153-1 (PMC11282724; doi:10.1186/s12917-024-04153-1)
Supplement: Supplementary file 1 — Supplementary Material 1 [file 12917_2024_4153_MOESM1_ESM.docx]

**Modulation of the intestinal mucosal and cell-mediated response against natural helminth infection in the African catfish *Clarias gariepinus***

**Sara Salah Abdel-Hakeem^1*^, Yousef Abdal Jalil Fadladdin^2^_,_ Mohsen A. Khormi^3^_,_ Hanan Hassan Abdel-Hafeez*^4^**

**^1^Sara Salah Abdel-Hakeem (ORCID: https://orcid.org/0000-0003-1069-5806)**

**Parasitology Laboratory, Zoology and Entomology Department, Faculty of Science, Assiut University (71526), Assiut, Egypt**

[**sara_assiut86@aun.edu.eg**](mailto:sara_assiut86@aun.edu.eg)

**Cellphone No: 00201014605359, Fax: ‏+20882342708**

**^2^Yousef Abdal Jalil Fadladdin (ORCID: https://orcid.org/0000-0003-2638-624X)**

**King Abdulaziz University, Faculty of Sciences, Department of Biological Sciences, Jeddah, Saudi Arabia**

[**yfadladdin@kau.edu.sa**](mailto:yfadladdin@kau.edu.sa)

**Cellphone No: +966554593341**

**^3^Mohsen A .Khormi**

**Department of Biology, College of ScienceJazan UniversityJazanKingdom of Saudi Arabia**

**[makhormi@jazanu.edu.sa](mailto:makhormi@jazanu.edu.sa)**

**^4^Hanan Hassan Abdel-Hafeez**

**Professor of cell and tissue, Department of Anatomy and Histology, Faculty of Veterinary Medicine, Assiut University, Assiut (71516), Egypt**

[**hhnnzz91@aun.edu.eg**](mailto:hhnnzz91@aun.edu.eg)

**Corresponding authors:**

**1-Sara Salah Abdel-Hakeem (ORCID: https://orcid.org/0000-0003-1069-5806)**

**Parasitology Laboratory, Zoology and Entomology Department, Faculty of Science, Assiut University (71526), Assiut, Egypt**

**Email address:** [**sara_assiut86@aun.edu.eg**](mailto:sara_assiut86@aun.edu.eg)

**Cellphone No: 00201014605359, Fax: ‏+20882342708**

**2-Hanan Hassan Abdel-Hafeez**

**Professor of cell and tissue, Department of Anatomy and Histology, Faculty of Veterinary Medicine, Assiut University, Assiut (71516), Egypt**

**hhnnzz91@aun.edu.eg**


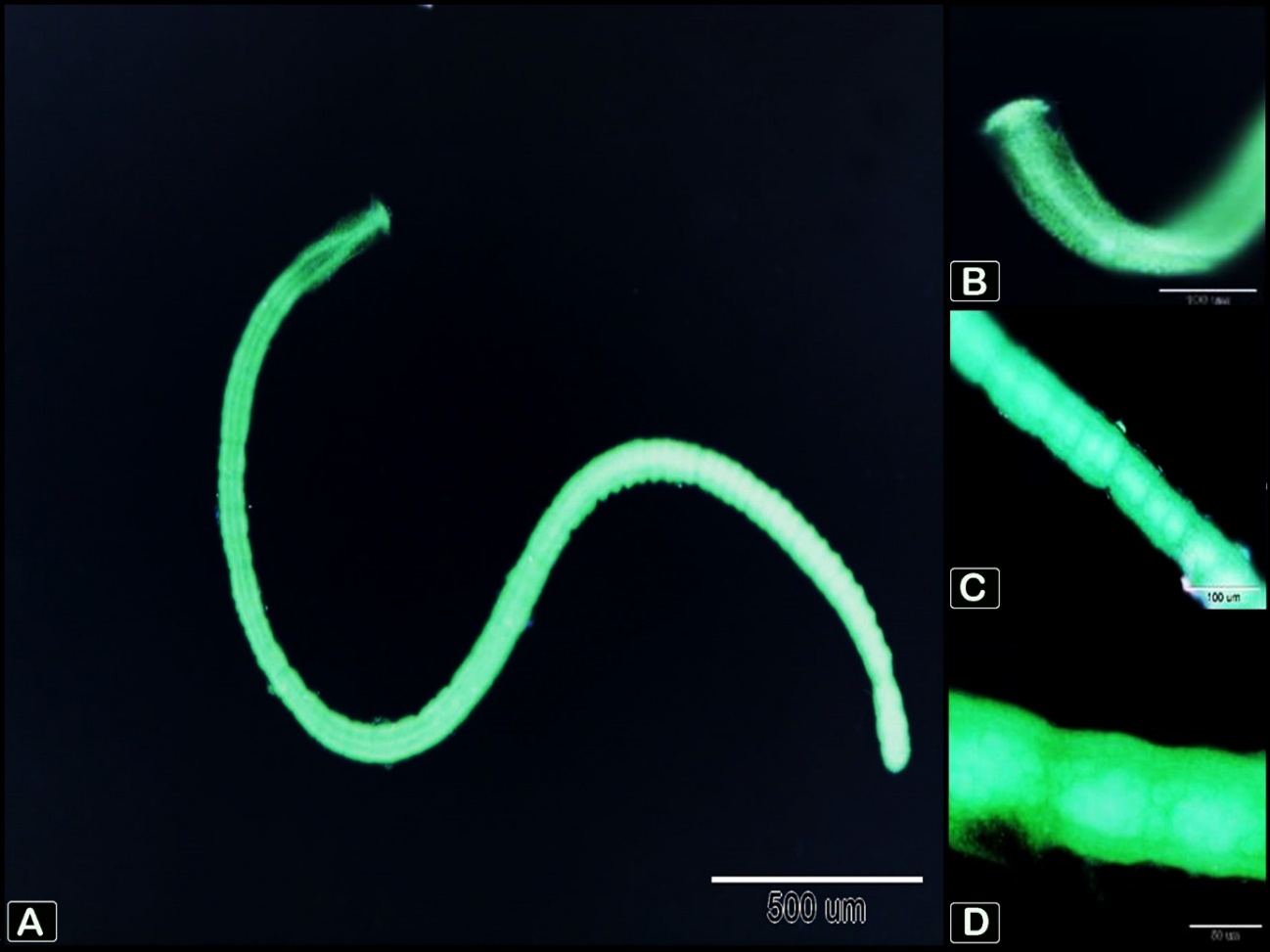


Supplementary figure 1: Negative of figure 1.


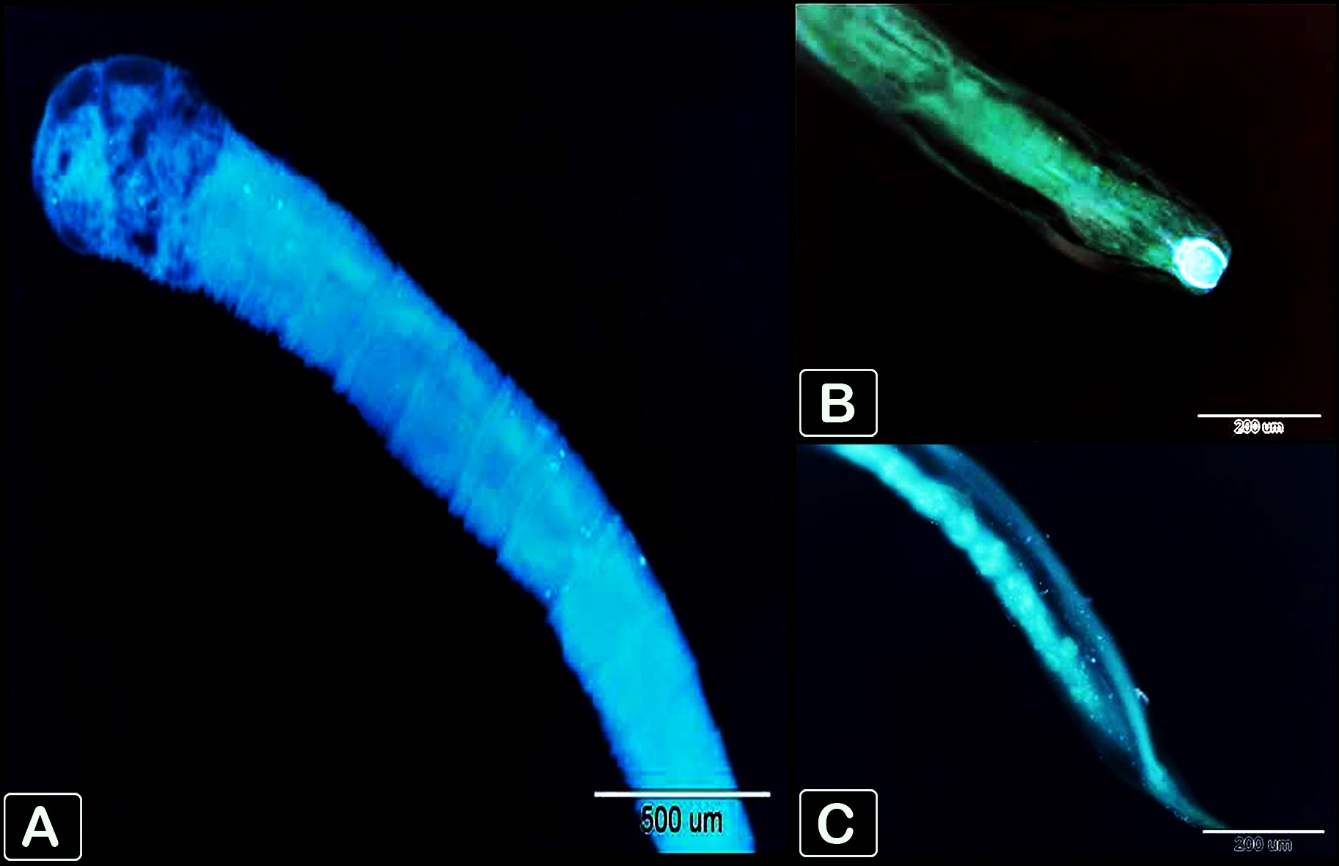


Supplementary figure 2: Negative of figure 2.


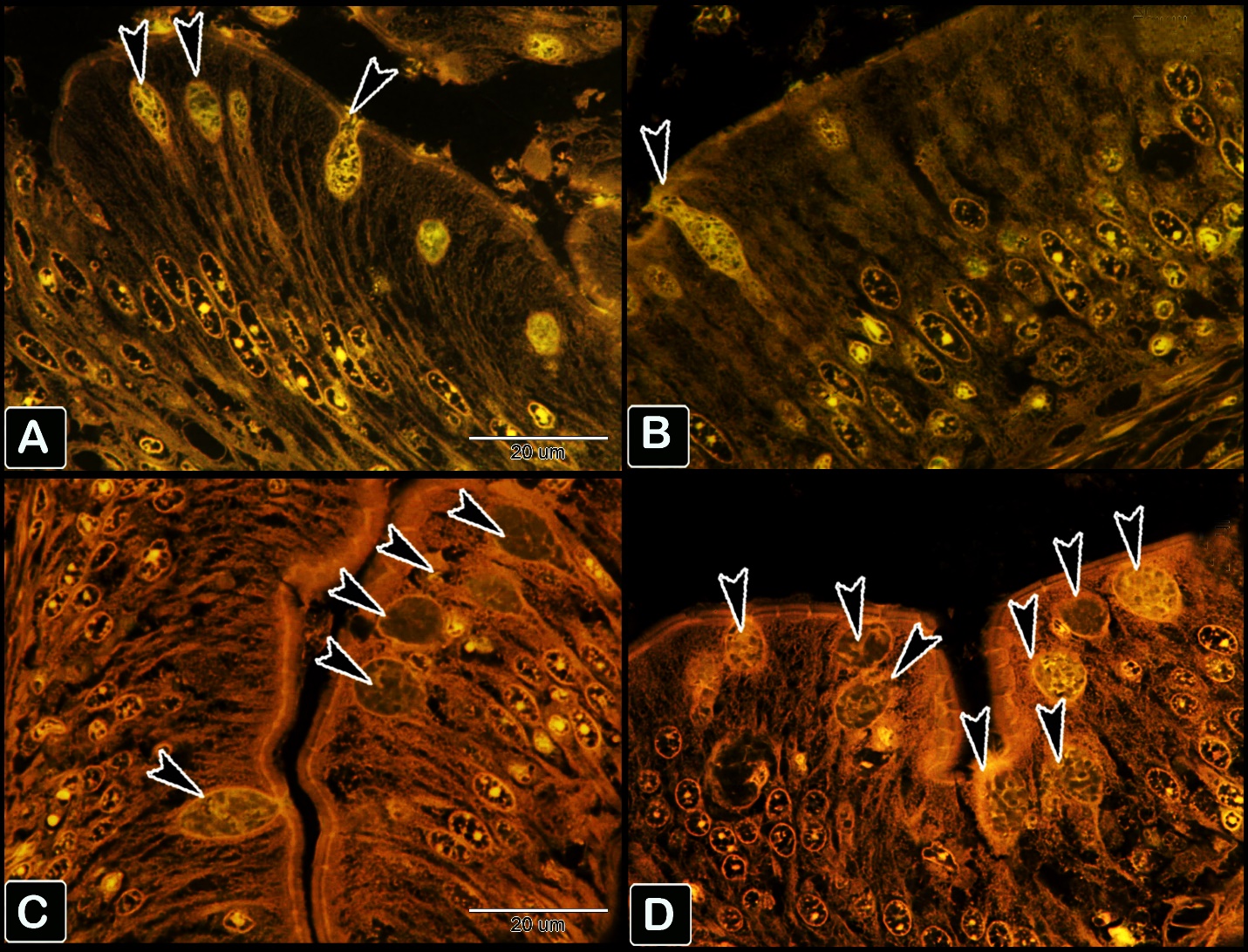


Supplementary figure 3: Negative of figure 3.


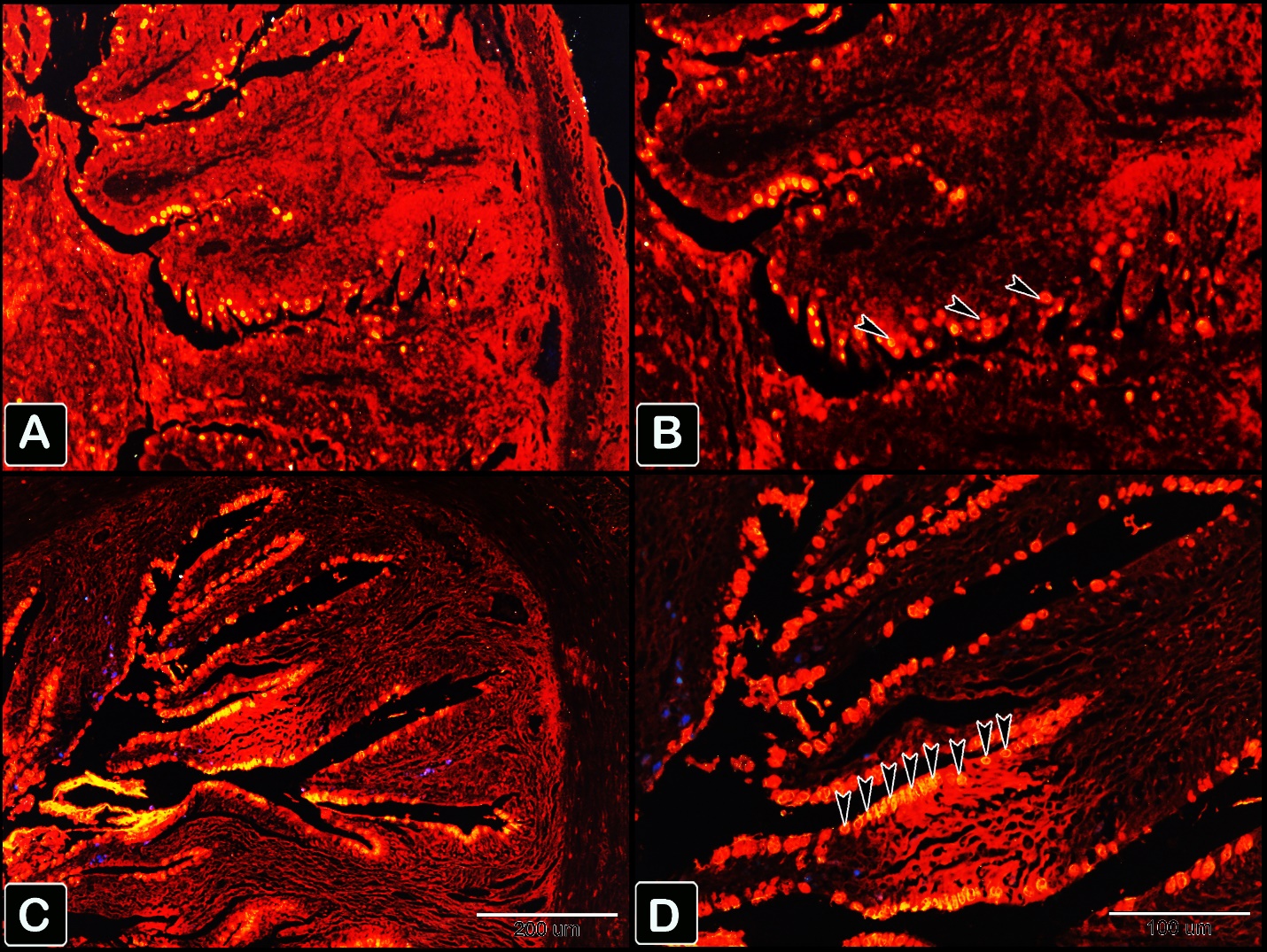


Supplementary figure 4: Negative of figure 4.


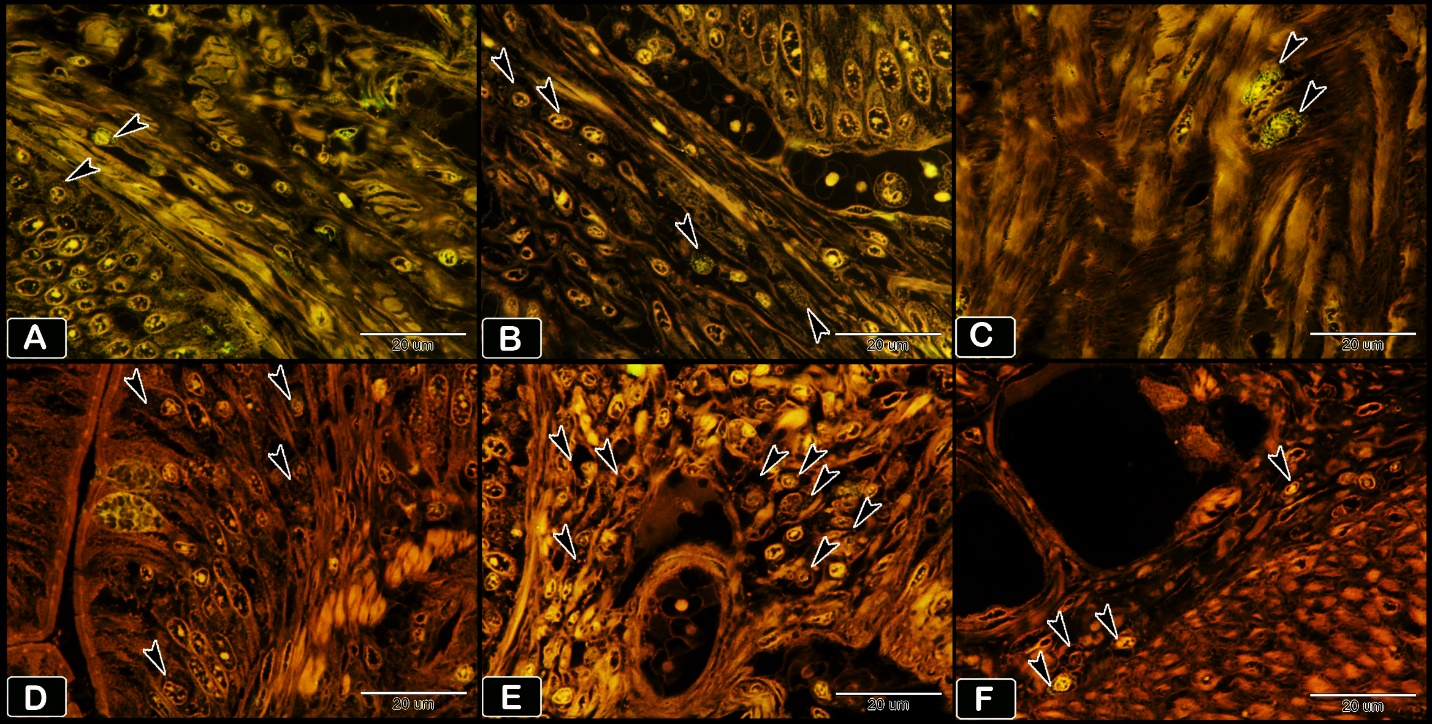


Supplementary figure 5: Negative of figure 6.


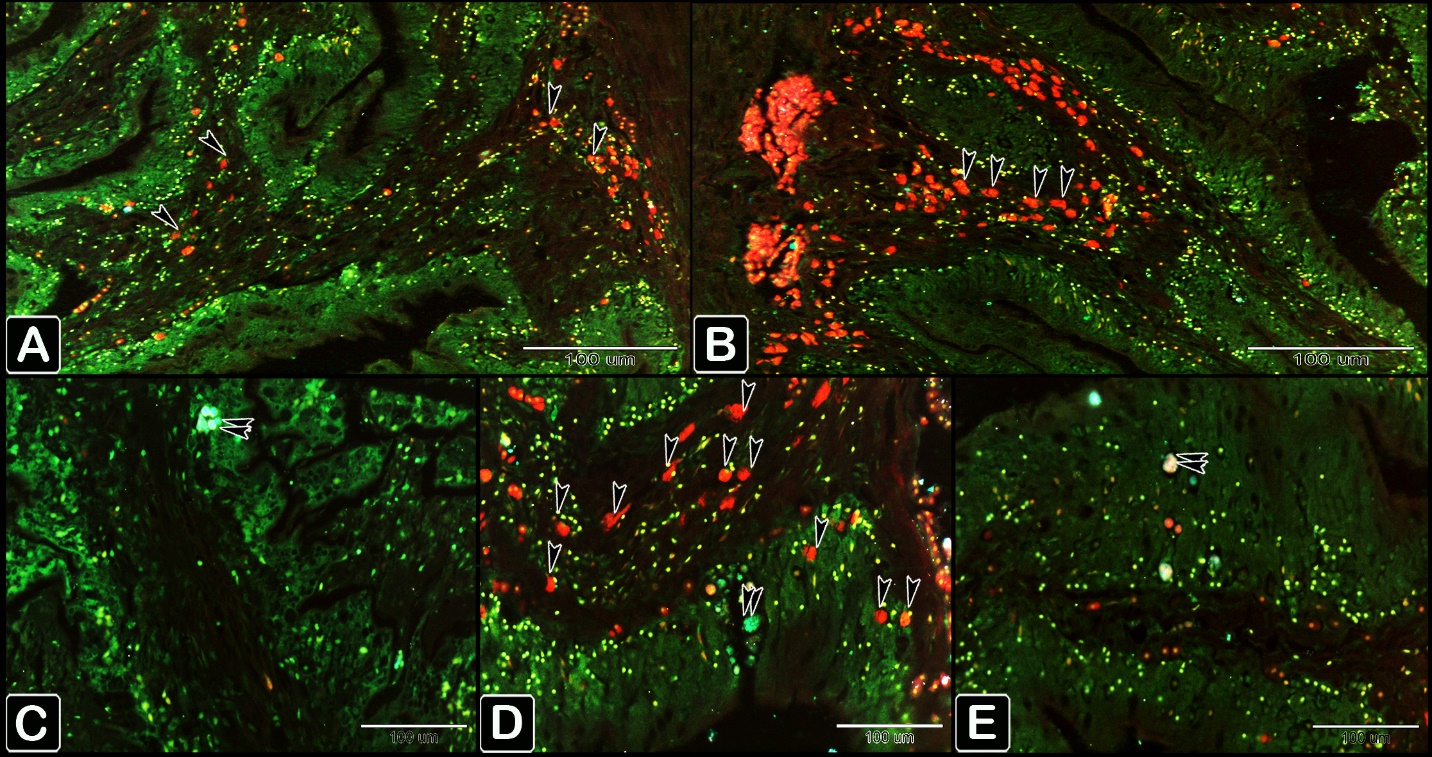


Supplementary figure 6: Negative of figure 7.


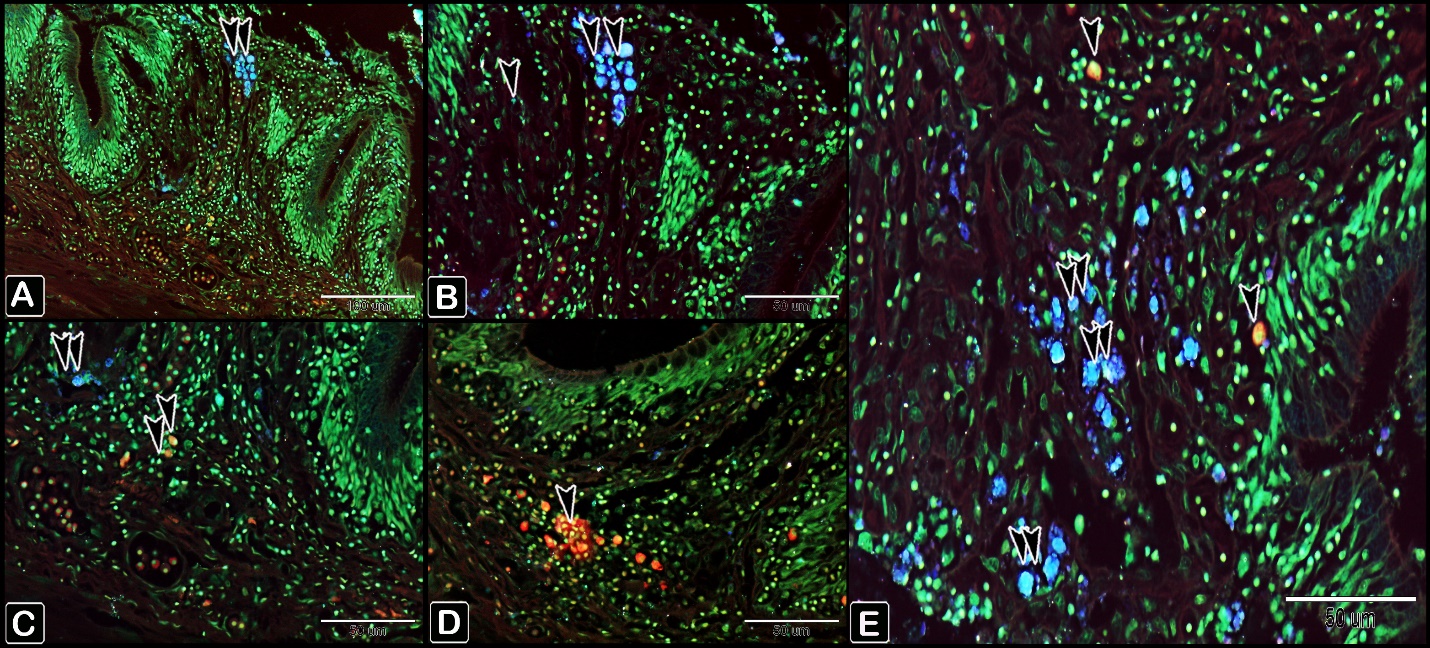


Supplementary figure 7: Negative of figure 8.


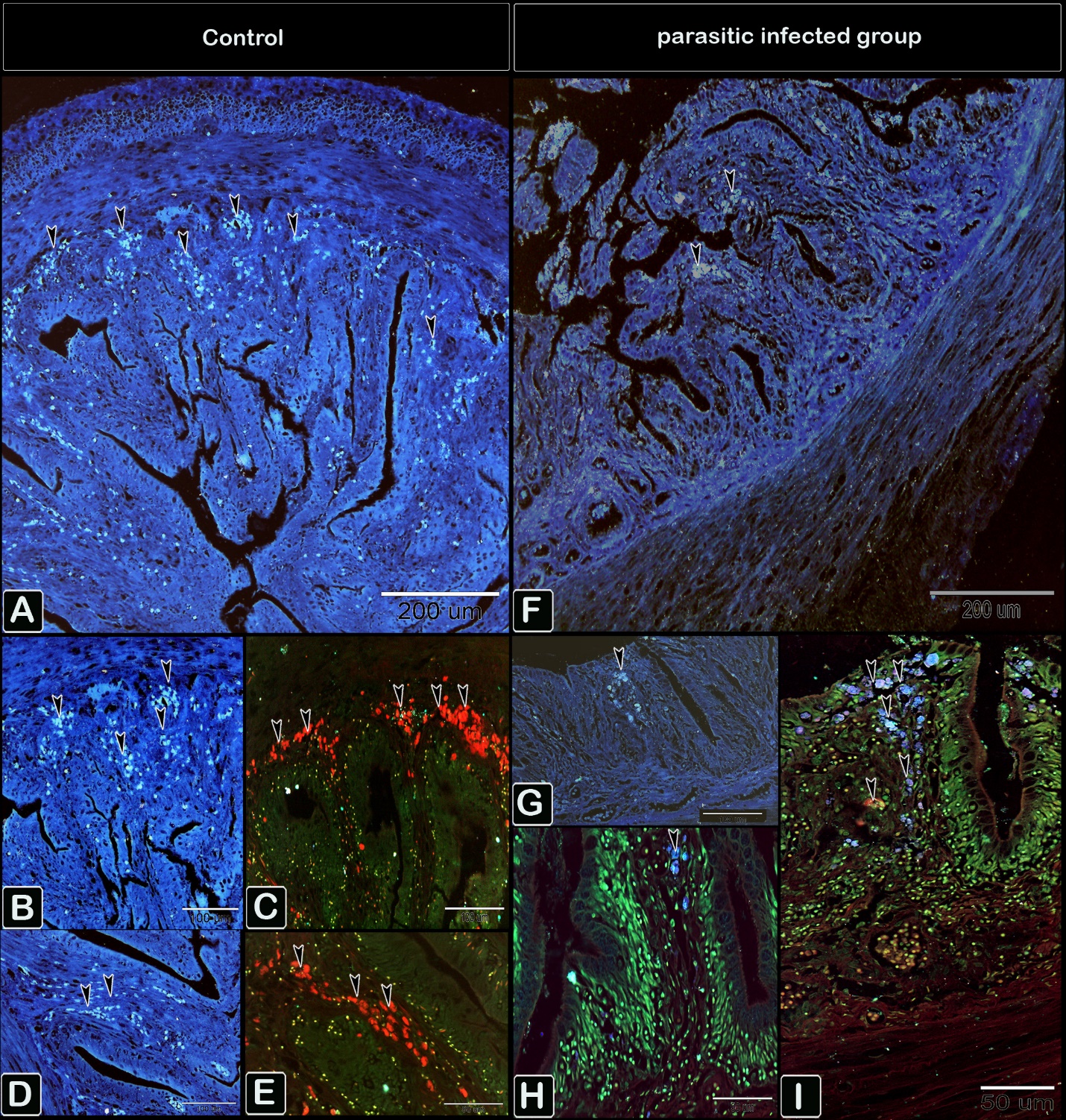


Supplementary figure 8: Negative of figure 9.


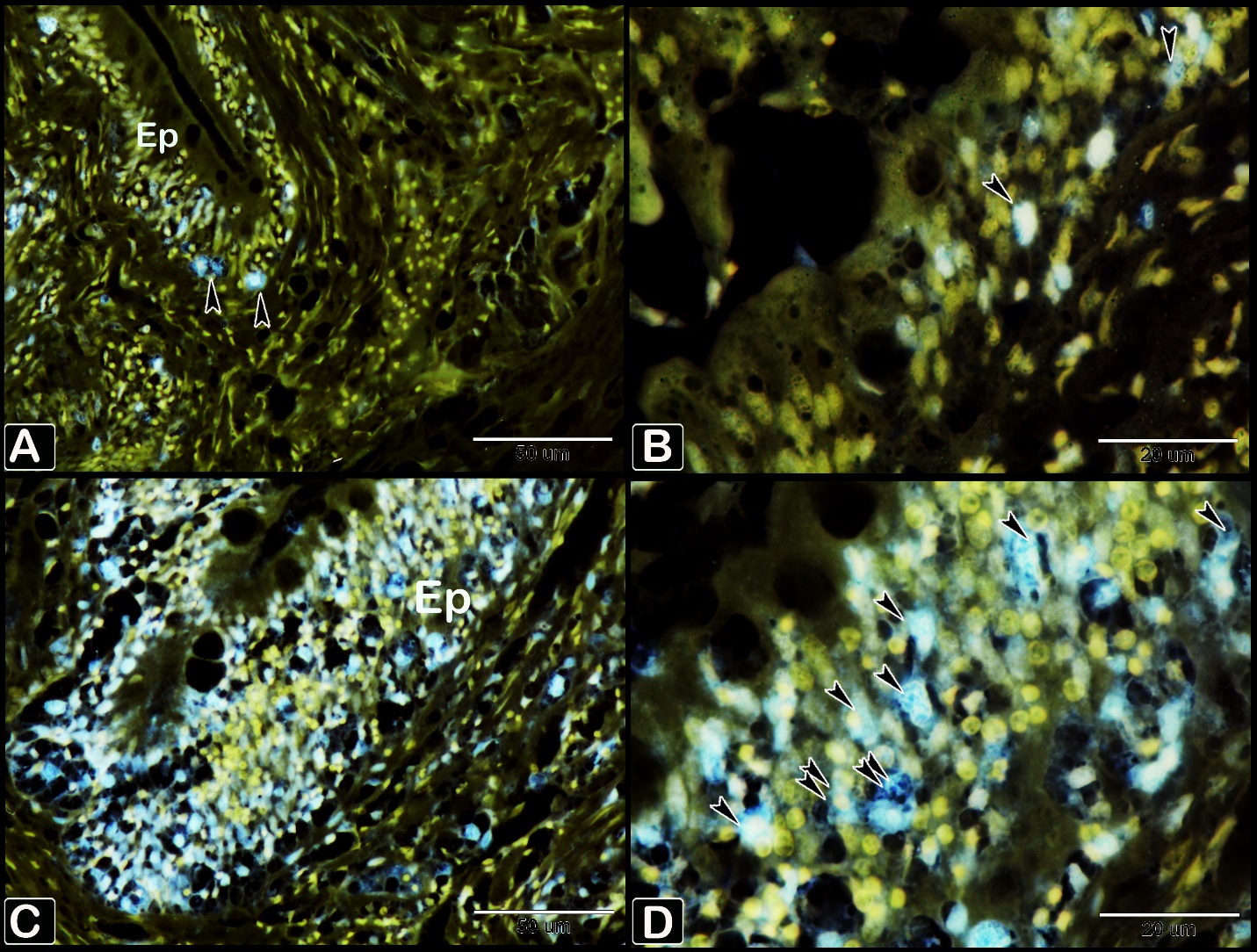


Supplementary figure 9: Negative of figure 10.


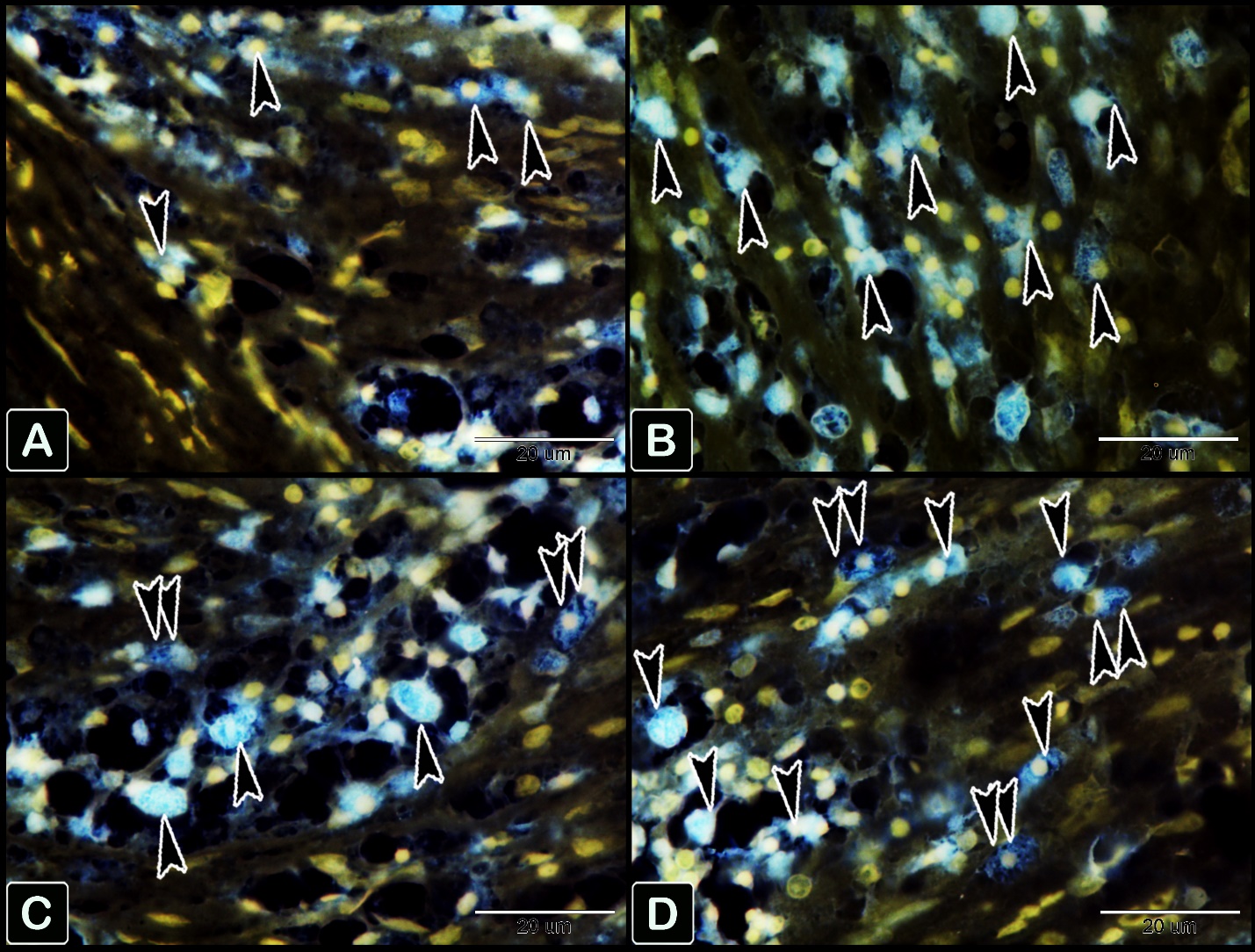


Supplementary figure 10: Negative of figure 11.


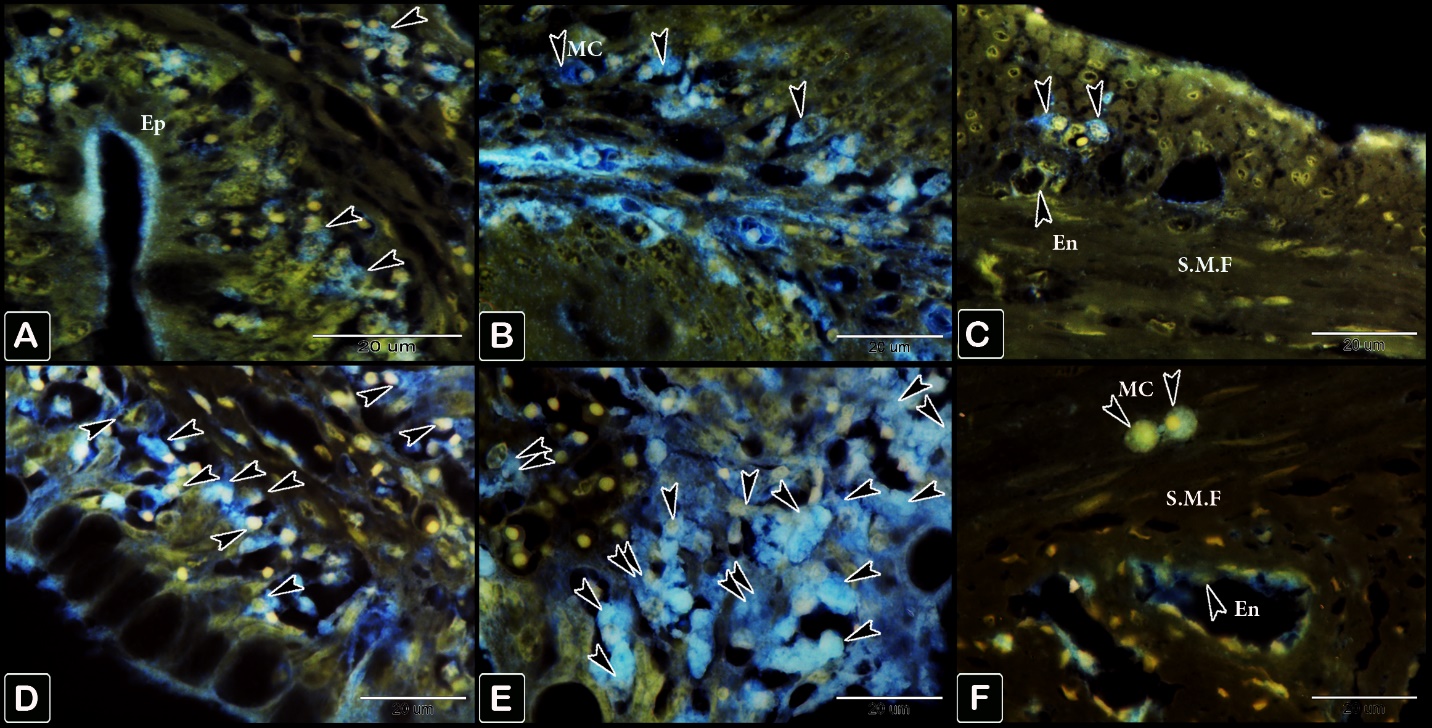


Supplementary figure 11: Negative of figure 12.


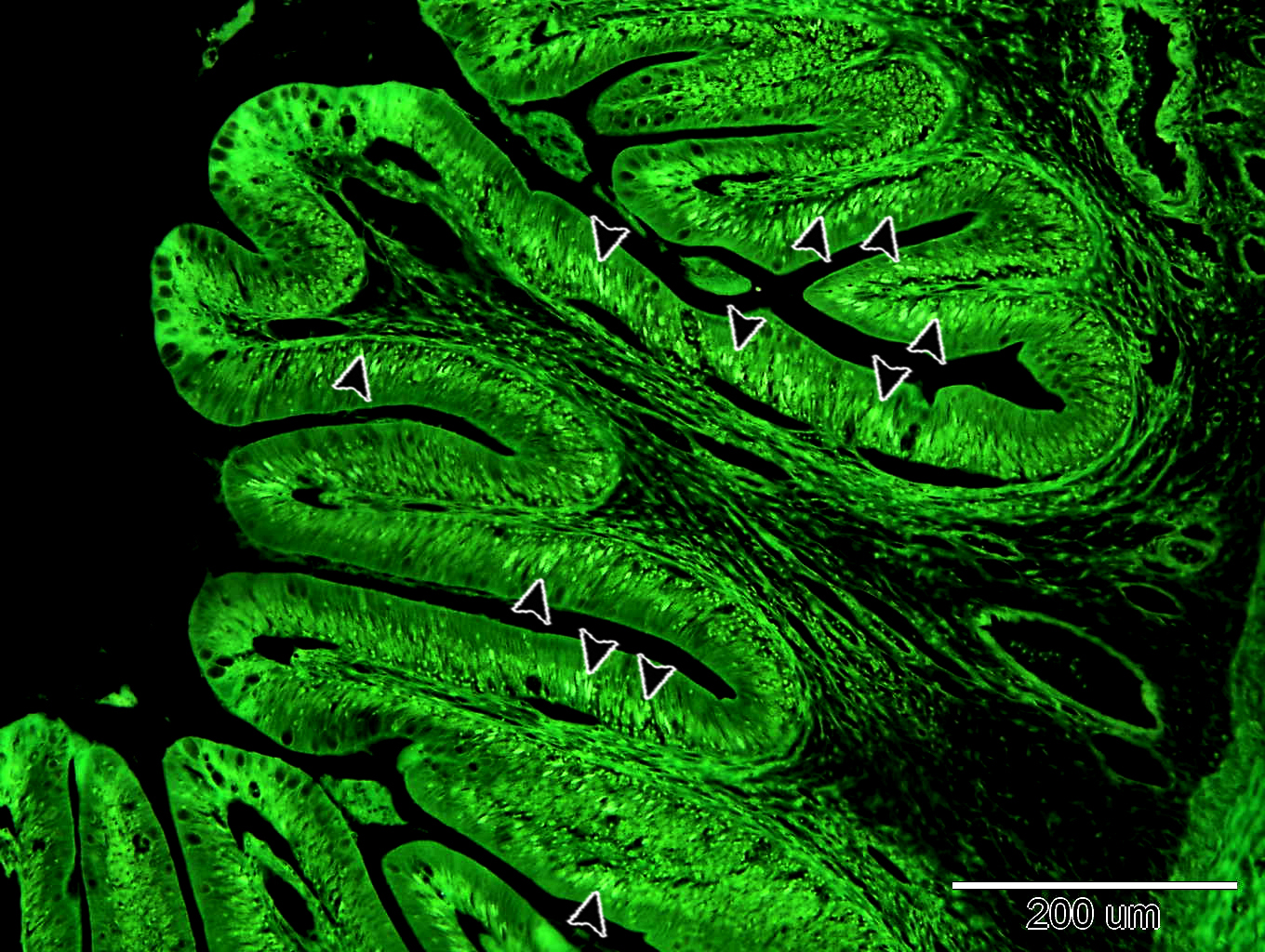


Supplementary figure 12: Negative of figure 14a.


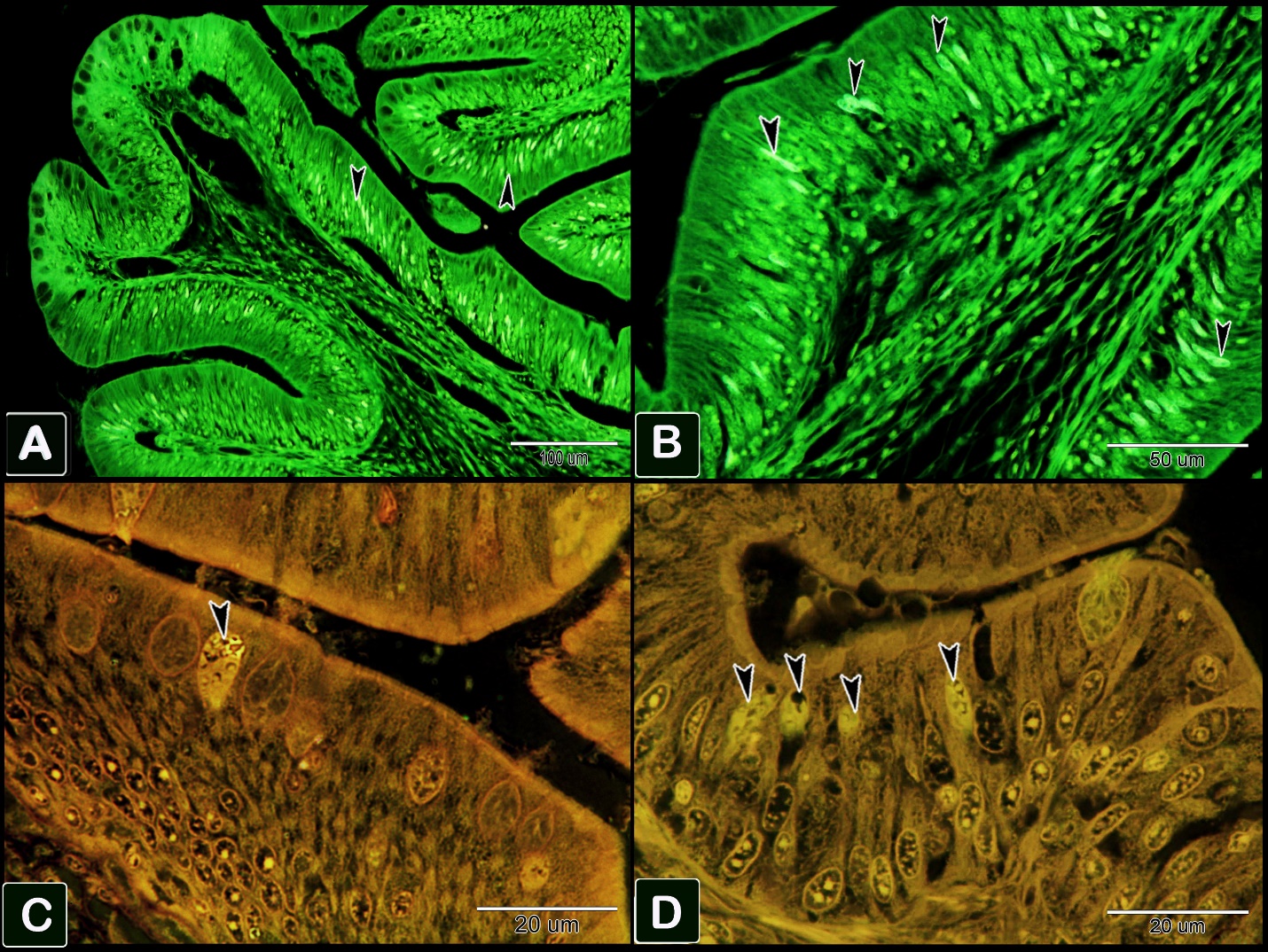


Supplementary figure 13: Negative of figure 14b.


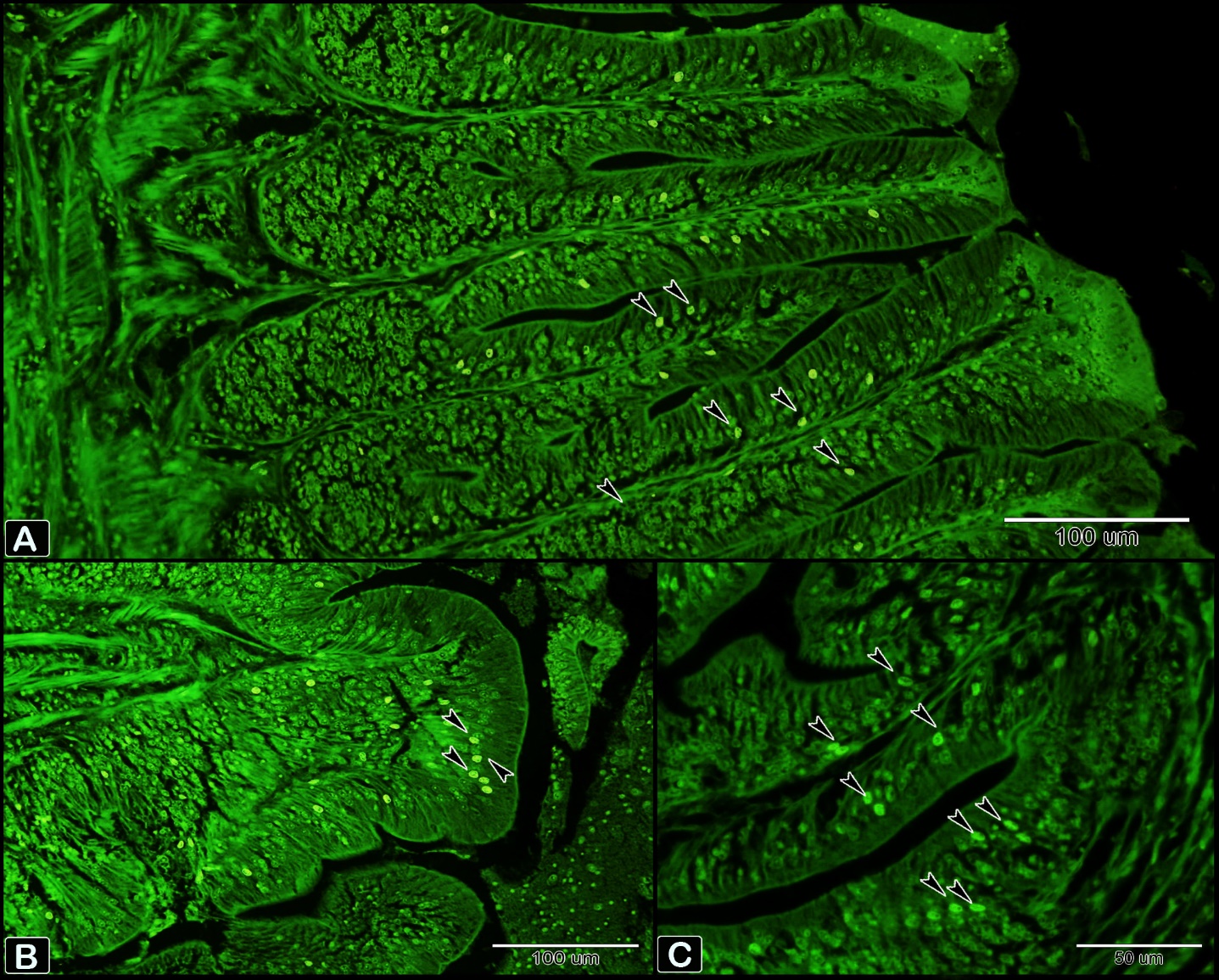


Supplementary figure 14: Negative of figure 15.


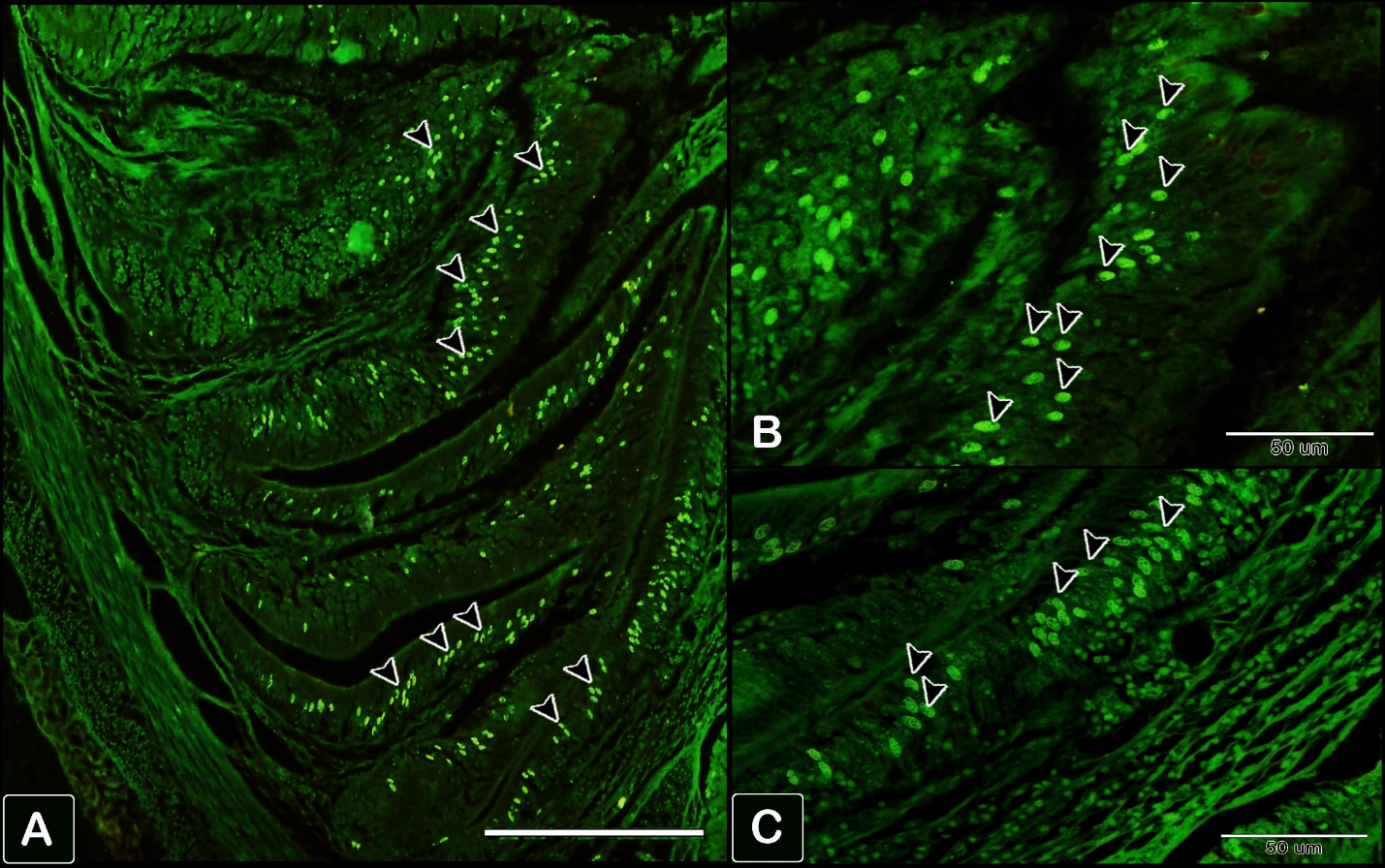


Supplementary figure 15: Negative of figure 15.


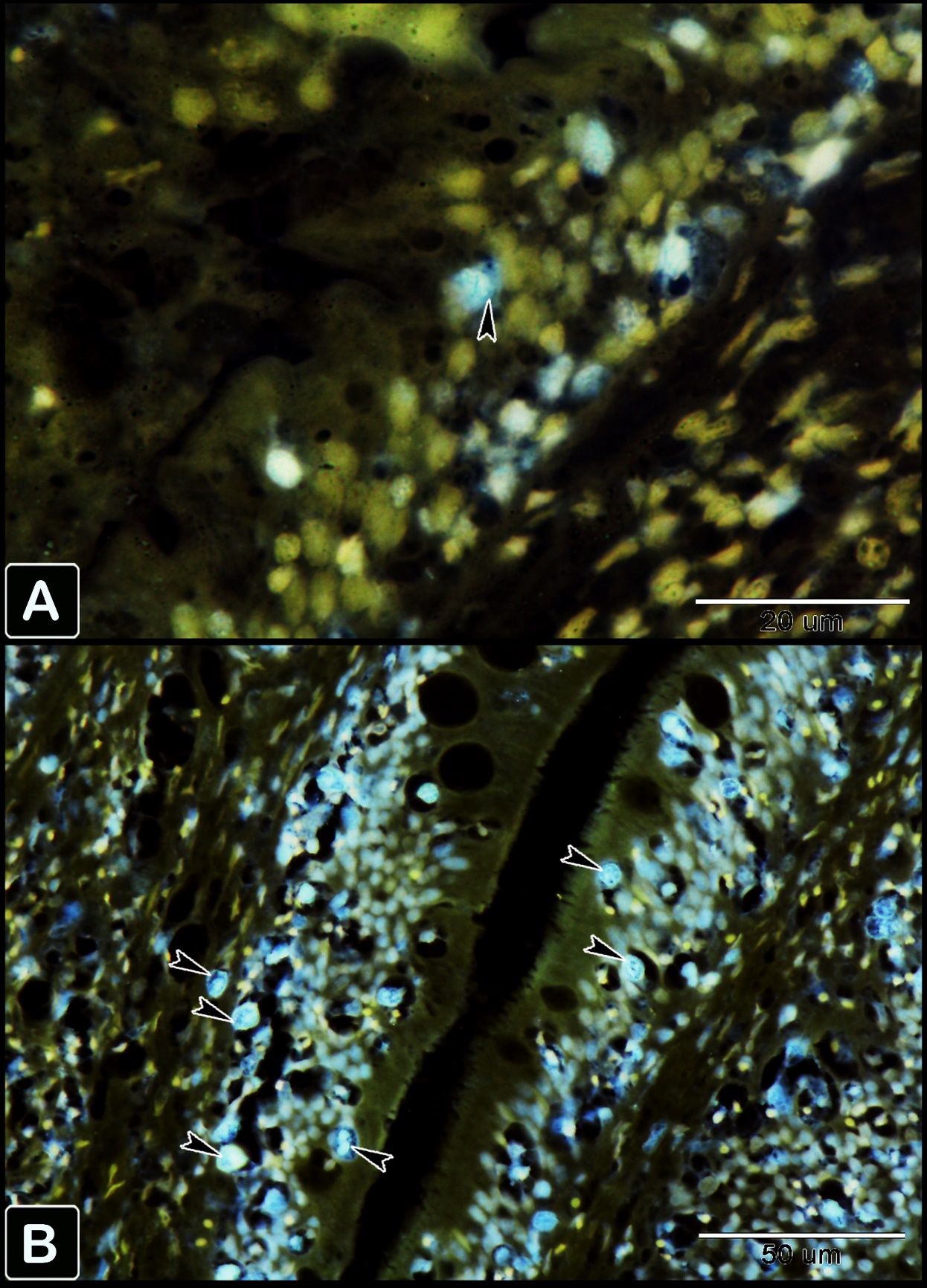


Supplementary figure 16: Negative of figure 17.


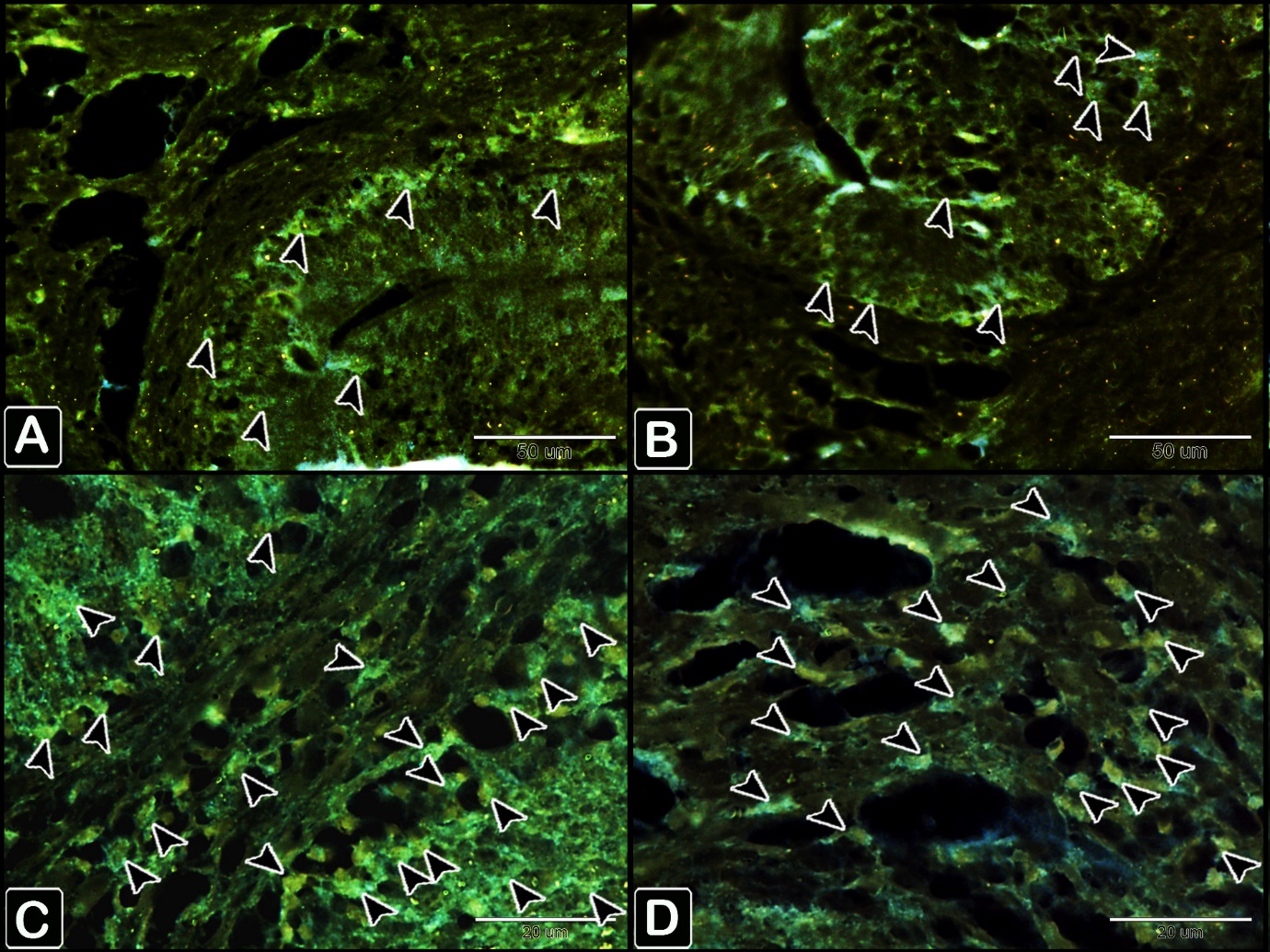


Supplementary figure 17: Negative of figure 18.


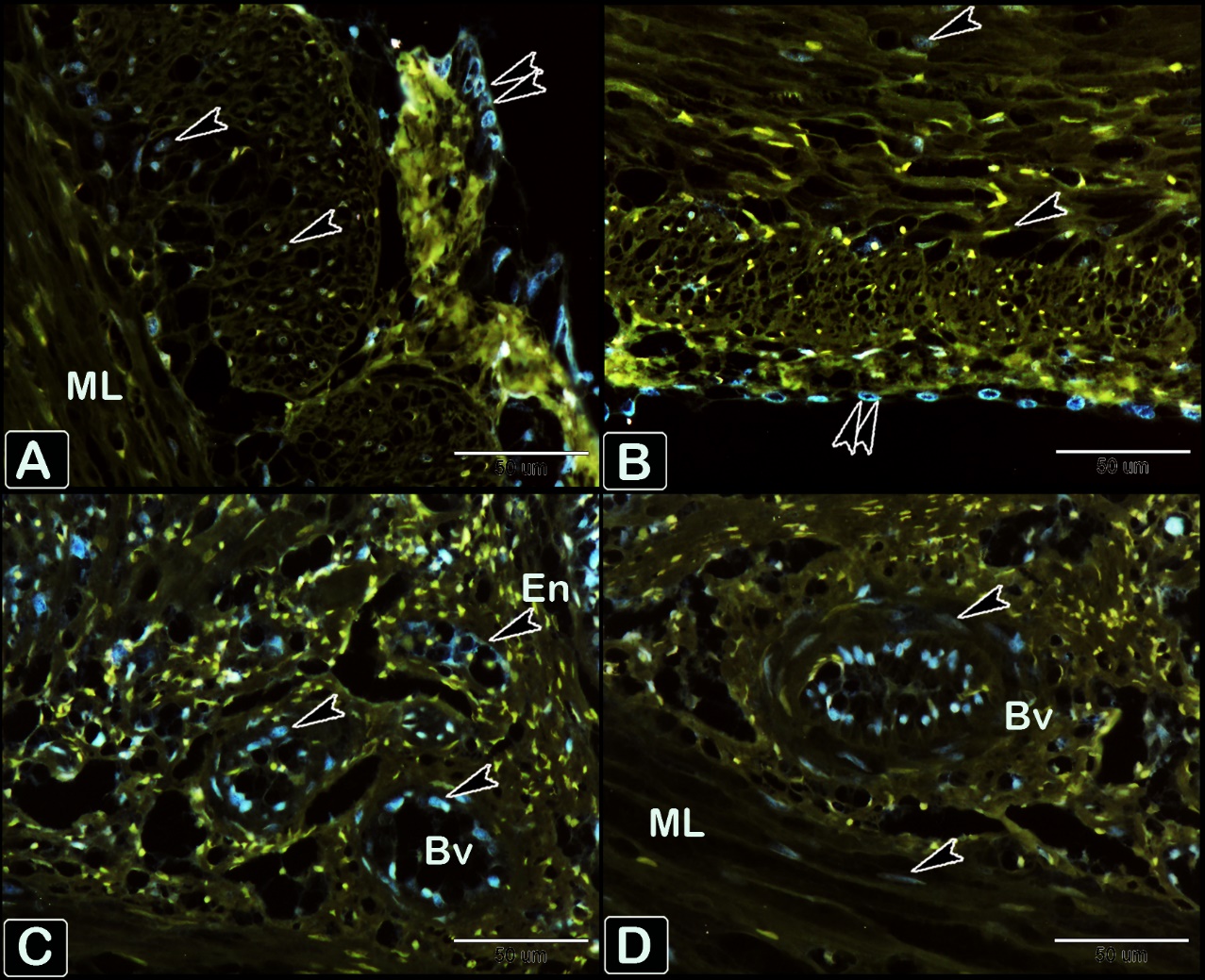


Supplementary figure 18: Negative of figure 19.


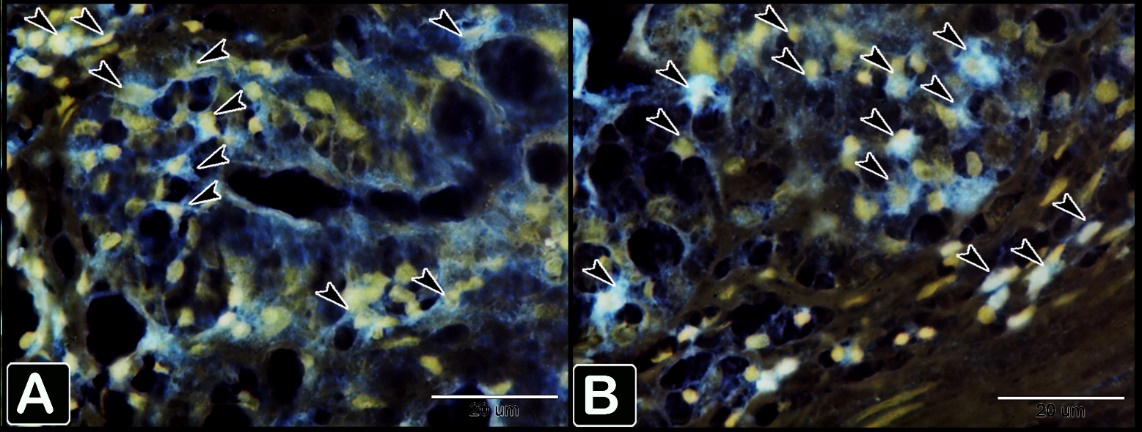


Supplementary figure 18: Negative of figure 20.


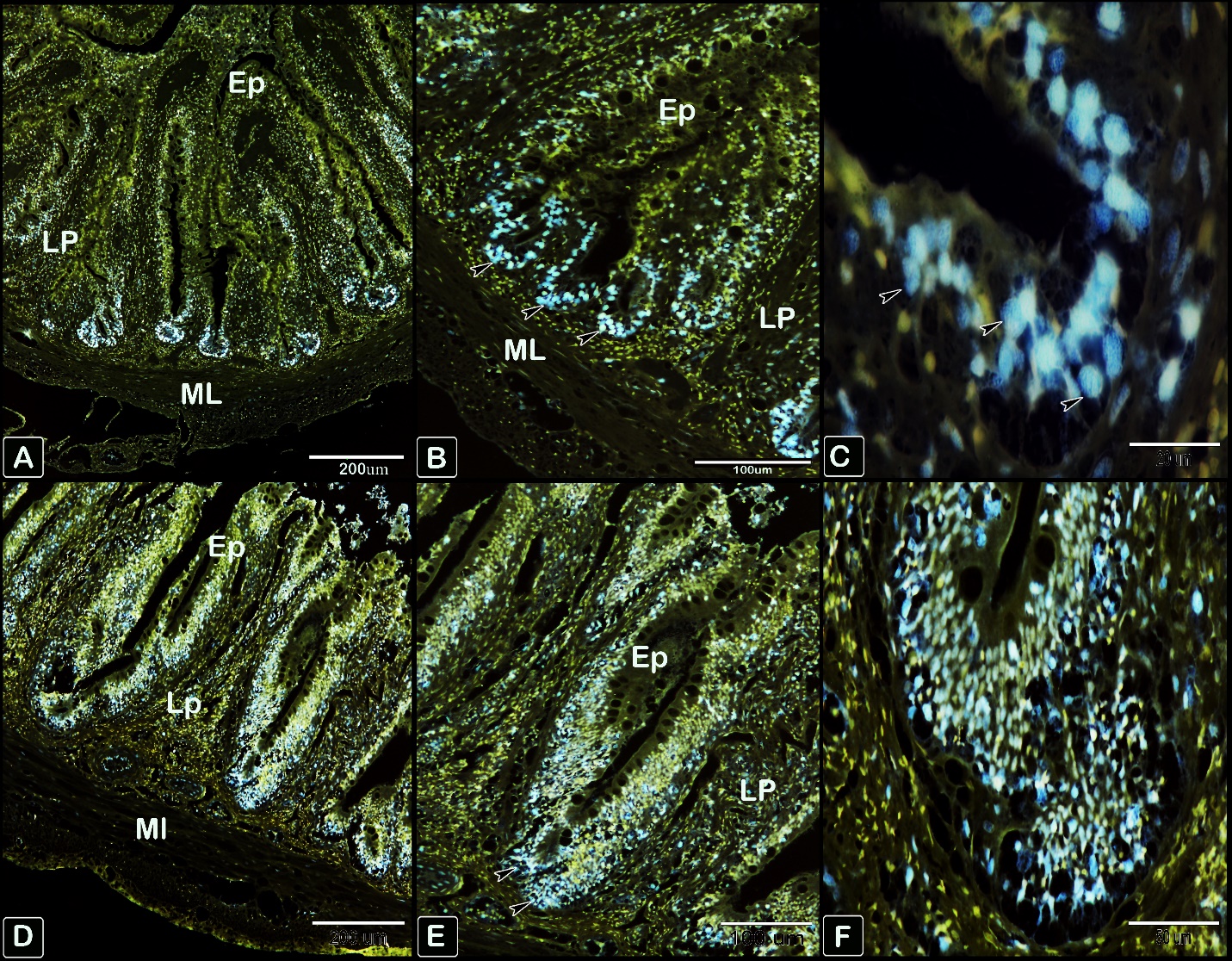


Supplementary figure 19: Negative of figure 21.


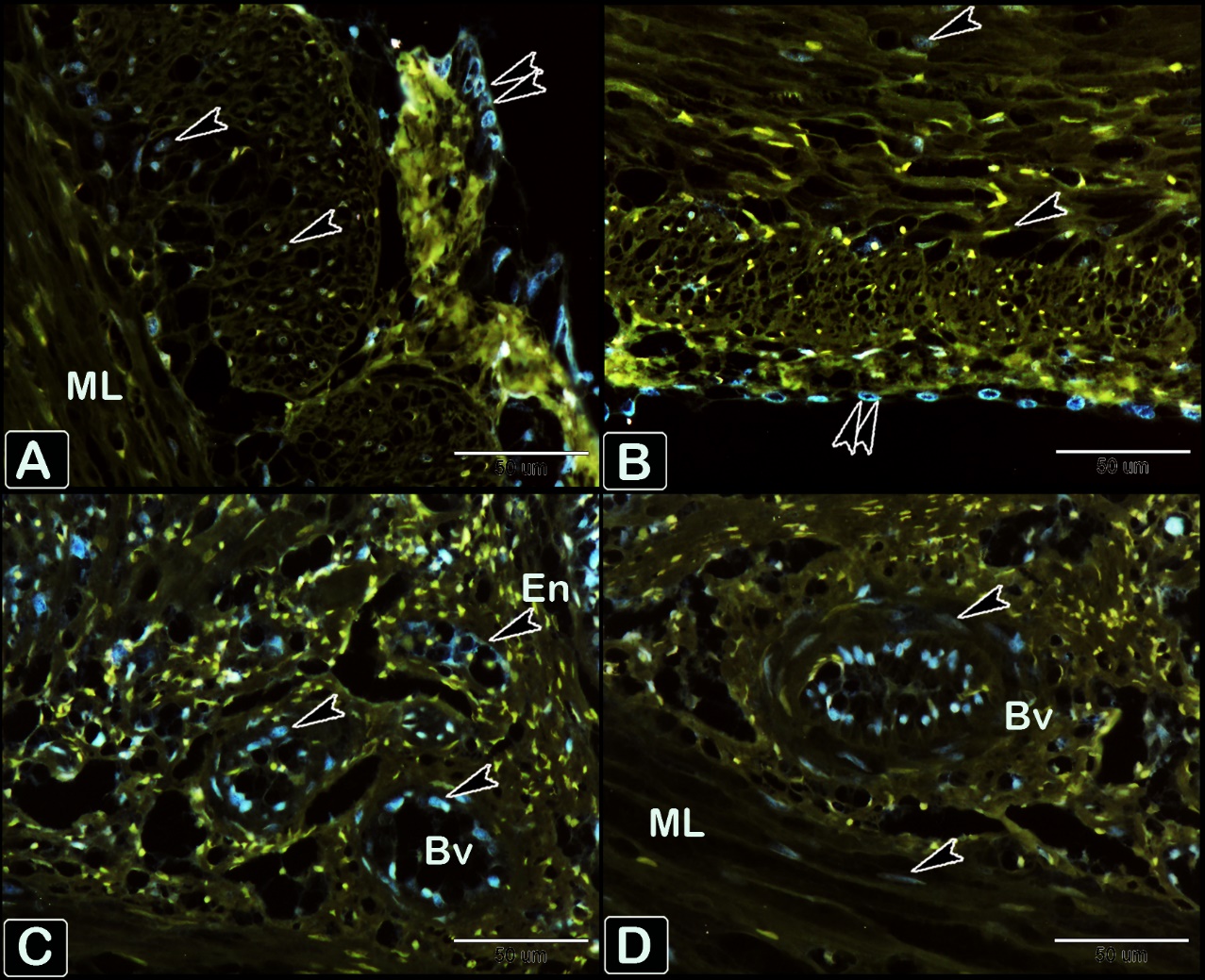


Supplementary figure 19: Negative of figure 22.


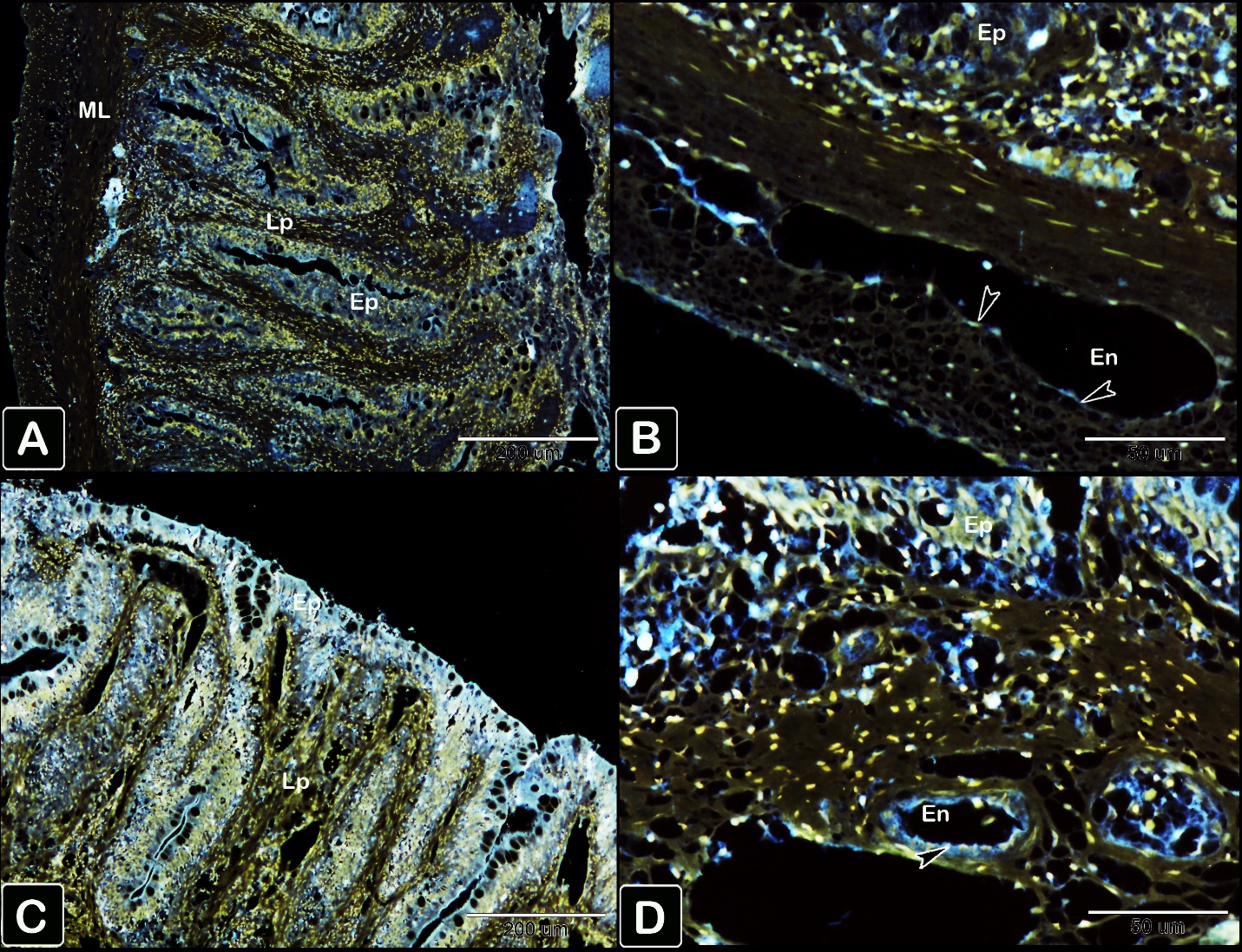


Supplementary figure 19: Negative of figure 23.


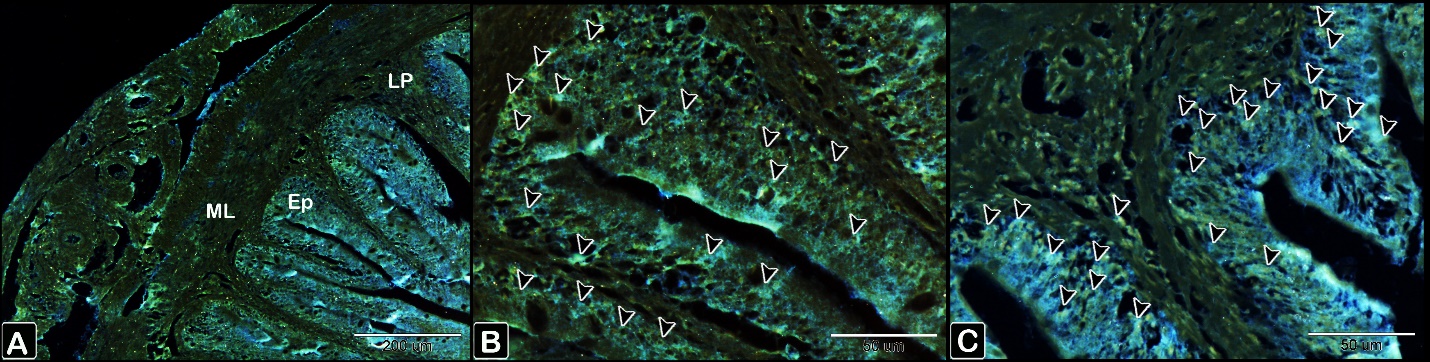


Supplementary figure 19: Negative of figure 24

0
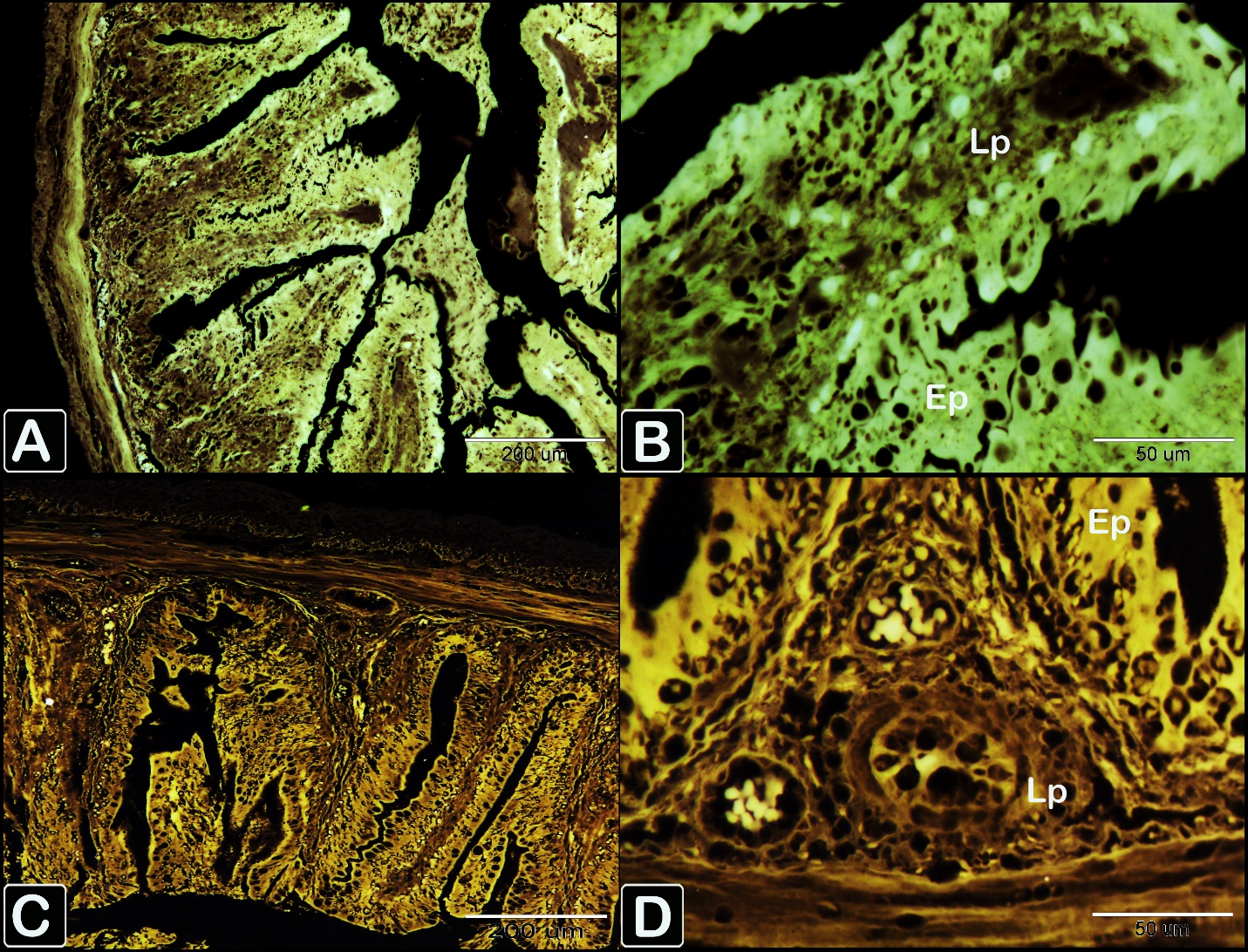


Supplementary figure 19: Negative of figure 25.


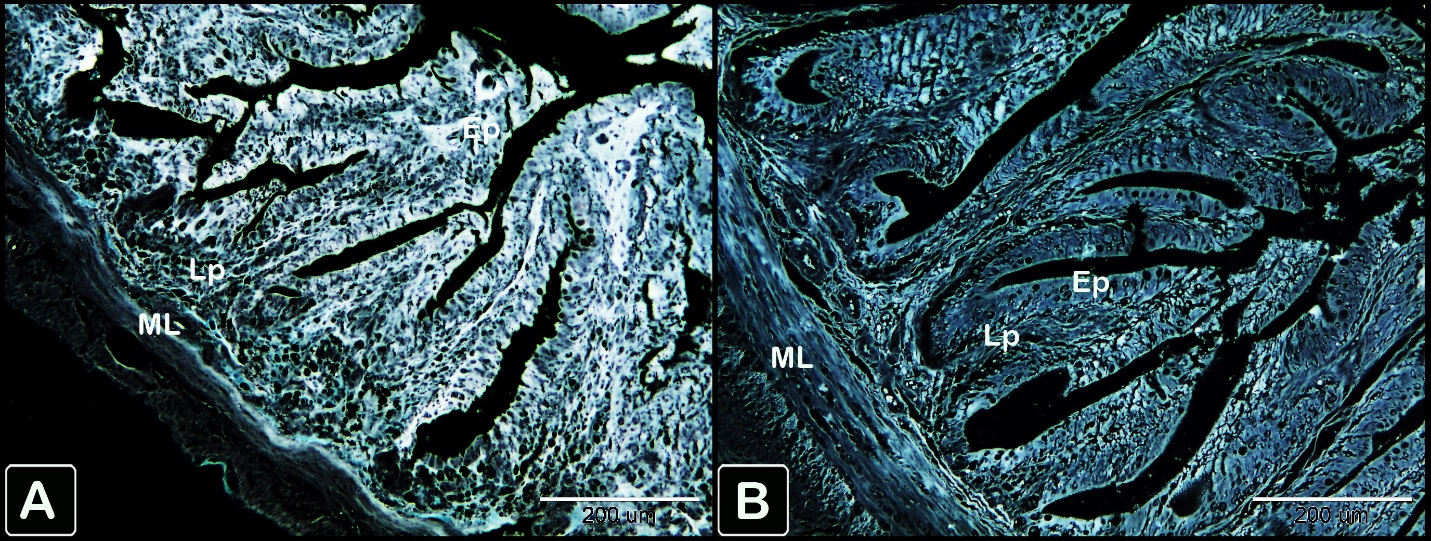


Supplementary figure 19: Negative of figure 26.
